# Therapeutic plasma exchange across multiple organ systems in an Egyptian tertiary center: A seven-year real-world cohort study

**DOI:** 10.1371/journal.pone.0358069

**Published:** 2026-09-11

**Authors:** Mohamed Ahmed El Maghawry, Reham Abd Elkhalek, Fatima Altaher Taha, Amira A. A. Othman, Aliaa Mohamed Abd El Khalik Ahmed, Dina A. Abdelhady, Fatma M. Attia Elsayed

**Affiliations:** 1 Internal Medicine Department, Faculty of Medicine, Zagazig University, Zagazig, Egypt; 2 Rheumatology and Rehabilitation Department, Faculty of Medicine, Zagazig University, Zagazig, Egypt; 3 Internal Medicine Department, Faculty of Medicine, Suez University, Suez, Egypt; 4 Neurology Department, Faculty of Medicine, Zagazig University, Zagazig, Egypt; 5 Clinical Pathology Department, Faculty of Medicine, Zagazig University, Zagazig, Egypt; Abdul Wali Khan University Mardan, PAKISTAN

## Abstract

**Background:**

Real-world evidence on therapeutic plasma exchange (TPE) from low- and middle-income countries remains limited. Egyptian data across multiple organ systems are scarce. This study aimed to evaluate the indications, safety, efficacy, and predictors of outcomes in patients undergoing TPE at a tertiary center in Egypt over 7 years.

**Methods and Findings:**

This retrospective cohort study included 221 consecutive patients who underwent TPE (2016–2022) at Zagazig University Hospitals, Egypt. Patients were stratified into renal, neurologic, hematologic, and metabolic groups. Primary outcomes were clinical response and all-cause mortality. Multivariate logistic regression, Cox proportional hazards models, and Kaplan–Meier survival analysis were performed. Among 221 patients (57.9% male; mean age 36.0 years), the most frequent indications were Guillain–Barré syndrome, thrombotic thrombocytopenic purpura, and myasthenia gravis crisis. ASFA category I indications constituted 77.8% of procedures, with response rates decreasing significantly across categories (*p* = 0.03). Overall response rate was 77.9% (complete remission in 93.0% of responders), with mortality 14.5%. Response rates exceeded 85% in autoimmune hemolytic anemia, hyperviscosity syndrome, and TTP. Neurologic indications achieved 81.6% response; renal indications showed lower response (73.2%) and highest mortality (21.3%). Adverse events occurred in 37.2% of patients, all mild-to-moderate with no session terminations. Independent mortality predictors included mechanical ventilation, creatinine >2.5 mg/dL, renal indication, hemoglobin <8 g/dL, while ASFA category I was protective (all aORs 2.67–5.22). In exploratory analyses, PLASMIC score ≥6 and time to TPE ≤ 2 days were associated with complete remission in TTP, while Hughes score ≥4 and time to TPE > 7 days were associated with poor functional outcome in GBS. These findings require external validation before clinical application. Diffuse alveolar hemorrhage (n = 9) demonstrated 100% mortality despite intervention, whereas SLE patients (n = 23) had 52.2% mortality, with 47.8% achieving complete remission or clinical improvement. A three-tier risk model stratified patients into high, intermediate, and low mortality risk groups. Independent predictors of clinical response included neurologic, hematologic, and metabolic indications compared to renal, ASFA category I, and ≥5 TPE sessions, while hemoglobin <8 g/dL and creatinine >2.5 mg/dL predicted poorer response.

**Conclusions:**

This single-center Egyptian TPE cohort demonstrates high efficacy and safety when aligned with ASFA guidelines. Neurologic and hematologic indications achieve optimal outcomes; renal indications and critical illness markers predict poorer prognosis. The PLASMIC score, treatment urgency in TTP and GBS, and the proposed three-tier model represent exploratory findings that, if prospectively validated, could become actionable prognostic tools. These findings suggest that evidence-based TPE expansion in resource-limited settings may be feasible, though multicenter validation is required.

## Introduction

Therapeutic plasma exchange (TPE) is an extracorporeal blood purification technique that removes circulating high-molecular-weight pathogenic substances, including autoantibodies, immune complexes, cryoglobulins, monoclonal proteins, cytokines, and lipoproteins [1,2]. It separates plasma from cellular blood components and replaces it with donor plasma, albumin, or crystalloid solutions, thereby rapidly modifying the humoral immune environment through a mechanism distinct from conventional immunosuppression [3]. Since its introduction in the mid-twentieth century, TPE has evolved into an established intervention across multiple medical disciplines, with the American Society for Apheresis (ASFA) currently recognizing 166 graded indications in hematology, neurology, nephrology, rheumatology, metabolic disorders, transplant medicine, and critical care [4].

In high-income countries, established apheresis infrastructure, dedicated training, and adherence to ASFA guidelines have yielded robust real-world evidence [4,5]. In contrast, low- and middle-income countries (LMICs) face substantial barriers, including prohibitive equipment costs, shortages of trained personnel, inconsistent replacement-fluid and blood-product availability, and limited national registries [6,7]. These limitations may contribute to delayed or absent access to TPE for patients with ASFA category I indications [6,8].

TPE efficacy varies according to disease pathophysiology. In thrombotic thrombocytopenic purpura (TTP), TPE removes inhibitory anti-ADAMTS13 autoantibodies while replenishing ADAMTS13 through fresh frozen plasma, restoring the cleavage of von Willebrand factor multimers and resolving microangiopathic hemolysis [9]. In Guillain–Barré syndrome (GBS) and myasthenia gravis, TPE removes pathogenic autoantibodies targeting peripheral nerve gangliosides and neuromuscular-junction acetylcholine receptors, respectively, with clinical improvement paralleling declines in antibody titers [10,11]. In hyperviscosity syndrome secondary to Waldenström’s macroglobulinemia or multiple myeloma, TPE reduces circulating monoclonal IgM or IgA, alleviating sludging and end-organ hypoperfusion [4]. In systemic lupus erythematosus (SLE), TPE removes immune complexes and autoantibodies implicated in diffuse alveolar hemorrhage, lupus cerebritis, and catastrophic antiphospholipid syndrome, although efficacy may be limited after irreversible tissue injury [12]. In diffuse alveolar hemorrhage, where underlying vasculitis may drive progressive tissue destruction, antibody removal alone may be insufficient to reverse established organ damage, potentially explaining the poor outcomes observed in this subgroup despite TPE.

Egypt, a lower-middle-income country, has witnessed progressive expansion of apheresis services, yet published data on adult TPE outcomes remain scarce. Only one adult multi-system cohort (n = 64, 2016) and one renal-focused series (n = 308, 2018) have been reported [13,14]. Pediatric experiences have also been documented but are not directly generalizable to adults [15,16]. No large-scale Egyptian study has systematically characterized TPE practice across multiple organ systems or identified independent predictors of treatment response and mortality. The only previous adult multi-system cohort is nearly a decade old and predates recent ASFA updates and advances in apheresis technology [13].

Without region-specific evidence, Egyptian clinicians must extrapolate from North American, European, or Asian cohorts, which differ in disease spectra, healthcare delivery models, and resource availability. Resource-constrained settings may require adaptive practices, including modified replacement-fluid protocols, extended inter-session intervals, alternative vascular access strategies, and triage based on anticipated benefit [7,17]. Whether such adaptations compromise efficacy or safety remains unknown. The absence of baseline epidemiological data also limits benchmarking against international standards, quality improvement, and evidence-based resource allocation [18].

The present study was conceived to address this evidence gap. We leveraged a seven-year consecutive cohort (01/01/2016–01/12/2022) from Zagazig University Hospitals, a major tertiary referral center in the Nile Delta region serving a catchment population of approximately ten million, to conduct the first comprehensive analysis of TPE practice in the Nile Delta region of Egypt. Our specific objectives were categorized as follows: ***Primary Objectives***: (1) To quantify treatment efficacy (clinical response rates, complete and partial remission patterns) and safety (adverse event frequency and severity) across the full cohort and by diagnostic group; (2) To identify independent predictors of 90-day all-cause mortality and clinical response through multivariate logistic regression and time-to-event (Cox proportional hazards) analyses. ***Secondary Objectives***: (1) To delineate the demographic characteristics, clinical indications, and ASFA category distribution among patients undergoing TPE across renal, neurologic, hematologic, and metabolic diagnostic groups; (2) To evaluate pre-specified disease-specific outcomes for five subgroups (TTP, GBS, MG crisis, SLE, and DAH); (3) To assess the association of procedural variables (replacement fluid type, vascular access) with outcomes using propensity score matching. ***Exploratory Objectives***: (1) To develop a hypothesis-generating three-tier risk stratification model based on synthesized multivariate and survival data; (2) To evaluate the PLASMIC score and treatment urgency as predictors of complete remission in TTP; (3) To identify predictors of poor functional outcome in GBS; (4) To explore the utility of NGAL as a biomarker in thrombotic microangiopathies (substudy).

We hypothesized that TPE, when deployed in accordance with ASFA guidelines, would demonstrate efficacy and safety profiles comparable to those reported in international cohorts, but that resource-sensitive adaptations, such as preferential use of temporary catheter over permanent fistula access and limited availability of fresh frozen plasma, might influence complication rates and outcomes. We further hypothesized that disease-specific response hierarchies would emerge, with certain conditions (thrombotic thrombocytopenic purpura, autoimmune hemolytic anemia, hyperviscosity syndrome, myasthenia gravis) exhibiting superior outcomes, while others (diffuse alveolar hemorrhage, advanced renal indications, severe Guillain–Barré syndrome with respiratory failure) would portend poorer prognosis. By systematically testing these hypotheses, we aimed not merely to describe local practice but to generate actionable evidence, identifying which patients benefit most, which complications are most preventable, and which practice modifications warrant prospective evaluation.

## Subjects and methods

### Study population and design

This retrospective cohort study was conducted at the Nephrology and Apheresis Unit of Zagazig University Hospitals, a major tertiary referral center in the Nile Delta region of Egypt serving a catchment population of approximately ten million inhabitants. Data were derived from a prospectively maintained institutional database (from which data were extracted retrospectively for this analysis) with clinical information, including adverse events, recorded in real-time at the point of care and subsequently extracted for analysis. The study period spanned seven consecutive years, from 01/01/2016 to 01/12/2022, coinciding with the establishment of a dedicated apheresis unit at Zagazig University Hospitals and the adoption of standardized TPE protocols based on the 2016 ASFA guidelines [4]. Data were accessed for research purposes between 01/01/2023 and 31/03/2023. All data were fully anonymized before analysis. The primary clinical follow-up endpoint was 90 days post-TPE initiation, consistent with the primary outcome of 90-day mortality. The study protocol was approved retrospectively by the Zagazig University IRB on 27/03/2022 (ZU-IRB #9357), as no prospective patient contact or intervention was involved (see Ethical Considerations section). The study was designed and reported in accordance with the Strengthening the Reporting of Observational Studies in Epidemiology (STROBE) guidelines for cohort studies [19].

A total of 244 consecutive patients underwent TPE during the study period. After applying exclusion criteria, 221 patients (90.6%) were included in the final analysis. Patients were stratified into four diagnostic groups based on the primary indication for TPE, as outlined in the ASFA categorization framework in effect during the study period (2016–2022, primarily 7th and 8th Editions) [4,6]. This classification approach was deliberately chosen because the primary aim of this study was to characterize the real-world spectrum of TPE practice at a tertiary referral center rather than to evaluate a single disease indication. While pooling across diagnostic groups introduces clinical heterogeneity, this approach reflects actual practice where TPE is used across multiple disciplines. We addressed this heterogeneity through: (1) stratification by diagnostic group with outcomes reported separately **(Tables 3 and 6)**; (2) disease-specific response criteria; (3) multivariate adjustment for diagnostic group as a covariate in regression models **(Table 10)**; and (4) pre-specified subgroup analyses for major disease-specific indications **(TTP, GBS, MG crisis, SLE, and DAH)**. For consistency, the 2023 ASFA framework [4] was used for reference, with the following clarification: severe hypertriglyceridemia was categorized as Category III during the study period (7th/8th Editions) and has since been upgraded to Category II in the 9th Edition [4]. Group I comprised 89 patients (40.3%) with renal indications, including antibody-mediated rejection, pre-transplant desensitization, ANCA-associated vasculitis, anti-glomerular basement membrane disease, IgA nephropathy, and recurrent focal segmental glomerulosclerosis. Group II comprised 13 patients (5.9%) with hematologic indications, including thrombotic thrombocytopenic purpura, autoimmune hemolytic anemia, hyperviscosity syndrome secondary to Waldenström’s macroglobulinemia or multiple myeloma, and other plasma cell dyscrasias. Group III comprised 114 patients (51.6%) with neurologic indications, including Guillain–Barré syndrome, myasthenia gravis crisis, chronic inflammatory demyelinating polyneuropathy, neuromyelitis optica, acute disseminated encephalomyelitis, and transverse myelitis. Group IV comprised 5 patients (2.2%) with metabolic indications, specifically severe hypertriglyceridemia with triglyceride levels exceeding 2000 mg/dL and familial hyperchylomicronemia syndrome.

**Classification of patients with multi-system diseases (TTP and SLE):** Thrombotic thrombocytopenic purpura (TTP) and systemic lupus erythematosus (SLE) are multi-system diseases that can present with overlapping renal, hematologic, and neurologic manifestations. To avoid double-counting and to ensure clinically meaningful group assignment, we classified each patient into **one** diagnostic group based on their predominant clinical presentation and primary management pathway at the time of TPE initiation, as follows:

- **TTP patients (n = 54 total):** 41 patients (75.9%) with predominant renal involvement (acute kidney injury requiring dialysis, kidney transplantation, or lupus nephritis) were classified under renal indications (Group I). The remaining 13 patients (24.1%) with predominant hematologic manifestations (isolated thrombocytopenia and microangiopathic hemolytic anemia without significant renal impairment) were classified under hematologic indications (Group II). This explains why the hematologic group (n = 13) contains exactly the number of TTP patients without renal predominance, while the renal group (n = 89) includes 41 TTP patients plus 48 other renal diagnoses.
- **SLE patients (n = 23 total):** These were distributed across diagnostic groups based on their predominant manifestation: catastrophic antiphospholipid syndrome and lupus nephritis (renal group), lupus cerebritis and Guillain-Barré syndrome (neurologic group), and TTP (hematologic group). For disease-specific SLE subgroup analysis (Table 7), all 23 patients were analyzed together regardless of their diagnostic group assignment.

This classification approach preserves the integrity of between-group comparisons (renal vs. neurologic vs. hematologic) while allowing disease-specific subgroup analyses (TTP, SLE, GBS, MG) to be performed on unified cohorts across groups. We acknowledge that this classification may introduce some misclassification bias, as both TTP and SLE are multi-system diseases. However, this approach reflects real-world practice where patients are managed primarily by the specialty responsible for their predominant manifestation, and it preserves the integrity of between-group comparisons while allowing disease-specific subgroup analyses on unified cohorts. Table 2 provides the complete breakdown of specific indications within each diagnostic group.

For disease-specific subgroup analyses (e.g., thrombotic thrombocytopenic purpura, Guillain–Barré syndrome, systemic lupus erythematosus), patients with these conditions were identified across diagnostic groups and analyzed as unified cohorts without duplication. This approach allows for between-group comparison based on the primary management pathway while preserving disease-specific outcome analysis.

Pre-specified subgroup analyses were conducted for patients with systemic lupus erythematosus (n = 23), diffuse alveolar hemorrhage (n = 9), thrombotic thrombocytopenic purpura (n = 54), Guillain–Barré syndrome (n = 54), and myasthenia gravis crisis (n = 30). Myasthenia gravis preoperative preparation cases (n = 4) were analyzed separately and not included in the myasthenia gravis crisis subgroup, as these represent a distinct clinical scenario with different ASFA categorization and outcomes. These five subgroups were selected before data analysis based on clinical relevance, adequate sample size (n ≥ 9), and identified evidence gaps in the Egyptian literature. Systemic lupus erythematosus was selected due to heterogeneous TPE indications, limited Egyptian outcome data, and the need for phenotype-specific prognostic stratification. Diffuse alveolar hemorrhage was selected due to its universally poor prognosis in published series and the need to evaluate TPE efficacy in the Egyptian context. Thrombotic thrombocytopenic purpura, the largest diagnostic subgroup, was selected to validate the PLASMIC score in the Egyptian population and to identify predictors of complete remission. Guillain–Barré syndrome, the largest neurologic subgroup, was selected to evaluate the impact of TPE timing on functional recovery in a resource-limited setting. Myasthenia gravis crisis was selected due to its high prevalence and the need to define optimal TPE regimens in Egyptian intensive care units. All subgroup analyses were specified before data extraction and analysis.

No formal sample size calculation was performed a priori, as this was a comprehensive cohort study including all eligible patients over a fixed seven-year period. The final sample of 221 patients represents, to our knowledge, the largest adult TPE cohort reported from Egypt to date. The multivariate mortality model included 32 events and 5 independent predictors, yielding an events-per-variable ratio of 6.4, which is within acceptable limits for exploratory clinical research [20]. For the TTP complete remission model (27 events, 7 variables, EPV = 3.9) and GBS poor outcome model (13 events, 6 variables, EPV ≈ 2.2), results should be interpreted as exploratory and hypothesis-generating due to the lower EPV ratios. For missing data, complete-case analysis was employed for the primary analysis. Sensitivity analysis using multiple imputation by chained equations with 20 imputed datasets was performed to assess the robustness of findings under missing-at-random assumptions; results were consistent across both approaches, and complete-case analysis is presented herein.

### Ethical considerations

This retrospective cohort study was approved by the Institutional Review Board of Zagazig University Faculty of Medicine (ZU-IRB #9357) on 27/03/2022 (initial approval; expired 27/03/2023), with renewal approved on 08/02/2026 (expires 08/02/2027). Data collection and analysis were completed before the initial approval expired on 27/03/2023; the 2026 renewal was obtained solely for manuscript preparation and journal submission requirements, as the study involved no ongoing patient contact or new data collection after the initial approval period. IRB approval was obtained retrospectively, as data from patients treated between 01/01/2016 and 01/12/2022 were extracted from existing medical records created during routine clinical care. The IRB permitted this under Egyptian national regulations (Ministerial Decree 296/2021) for minimal-risk retrospective research involving no direct patient contact.

Informed consent: Because this study involved only the analysis of de-identified, pre-existing medical records, with no direct patient contact or intervention, the requirement for written informed consent was waived by the Zagazig University IRB. No research consent was obtained from any patient or legally authorized representative. The reason written consent could not be obtained was the retrospective nature of the study: contacting all 221 patients (including 32 who died during follow-up) was not feasible without prospective patient contact, which this retrospective design specifically excluded. This determination is consistent with Egyptian national regulations (Ministerial Decree 296/2021, Article 18) and the Declaration of Helsinki (2013 revision), which permit waiver of consent for retrospective research using existing medical records when the research involves no more than minimal risk and cannot practicably be carried out without the waiver.

Data anonymization and confidentiality: Patient data were extracted from electronic medical records and paper-based apheresis logs. Immediately after extraction, data were pseudonymized using unique study identification numbers. Direct identifiers (names, national identification numbers, exact dates of birth) were removed before analysis. The linking file connecting identifiers to study numbers was stored on a password-protected, encrypted hospital server accessible only to the principal investigator.

Confirmation of ethical compliance: The authors confirm that all research was performed in accordance with the ethical principles of the Belmont Report, the Declaration of Helsinki (2013 revision), and Egyptian national regulations for biomedical research.

Human Participants Checklist: The completed PLOS Human Participants Research Checklist is submitted as Supporting Information (S1 Checklist).

### Eligibility criteria

All adult patients aged 18 years or older who underwent at least one complete session of therapeutic plasma exchange at Zagazig University Hospitals during the study period were eligible for inclusion. All patients were confirmed to be aged ≥18 years at the time of TPE initiation through verification of national identification cards. The reported age range reflects the minimum and maximum ages in the final cohort after application of all exclusion criteria. Patients were required to have complete medical records available for review, including demographic data, baseline clinical and laboratory assessment before the first TPE session, procedural details (number of sessions, replacement fluid type, vascular access, anticoagulation), and documentation of outcomes, including response assessment, complications, and mortality status. Both male and female patients were included.

Exclusion criteria were applied sequentially. Patients younger than 18 years were excluded, as pediatric TPE follows distinct protocols and ASFA indications, and pediatric data from this institution have been reported separately [15]. Patients with incomplete medical records, defined as missing 20% or more of key variables, were excluded; this included missing baseline creatinine (n = 3), missing outcome assessment (n = 5), and missing number of sessions (n = 4). Patients who received fewer than 50% of prescribed TPE sessions due to refusal or loss to follow-up were excluded, as treatment adequacy could not be ascertained. Pregnancy was an exclusion criterion, as physiological changes in pregnancy alter plasma volume calculations and replacement fluid requirements, representing a distinct clinical entity. Patients with multiple admissions during the study period were counted once, using data from their first TPE episode. Patients transferred from other hospitals mid-treatment were included only if complete pre-transfer records were available and they received at least 50% of prescribed sessions at our institution; otherwise, they were excluded due to the inability to ascertain treatment adequacy and outcome data. No patients were excluded based on ASFA category IV indications, as no such indications were identified during the study period, nor were any patients excluded due to concurrent enrollment in interventional clinical trials.

### Data source and completeness

Data were abstracted from three complementary sources: (1) the electronic medical record system (Zagazig University Hospital Information System), (2) paper-based apheresis procedure logs completed in real-time by treating apheresis nurses, and (3) departmental quality assurance databases. Cross-referencing these sources ensured complete identification of all patients undergoing TPE during the study period.

Quality control measures included: (1) double data extraction by two independent investigators with reconciliation by a third senior investigator; (2) validation of a random 10% sample against original source documents (>95% concordance required); (3) systematic querying for missing data elements with return to source records; and (4) documentation of missingness rates for all key variables (reported in Statistical Analysis).

### Clinical and anthropometric assessment

At enrollment, all patients underwent a comprehensive clinical evaluation performed by board-certified nephrologists and, where indicated, neurologists and hematologists. Baseline assessments included detailed medication reconciliation with particular attention to prior immunosuppressive therapy, corticosteroid exposure, anticoagulant use, and nephrotoxic agents. Thorough physical examination was performed with systematic documentation of vital signs, volume status, neurological deficits using the Hughes disability scale for Guillain–Barré syndrome, and the Myasthenia Gravis Foundation of America clinical classification for myasthenia gravis, and bleeding manifestations in thrombotic microangiopathy patients.

Body weight was measured using a calibrated bed scale, with subtraction of estimated ascites volume in patients with nephrotic syndrome or liver disease, for use in plasma volume calculation via Kaplan’s equation [2]. Blood pressure measurements followed American Heart Association guidelines using automated cuffs with appropriate cuff sizing, taken after 10 minutes of rest in the supine position. All findings were recorded on standardized case report forms adapted from the American Society for Apheresis registry protocols.

For patients with thrombotic thrombocytopenic purpura, the PLASMIC score was calculated from clinical and laboratory data that had been recorded prospectively as part of routine care, but the research use of these scores was retrospective. The score was calculated by two independent reviewers using the seven-component scoring system: platelet count <30 × 10^3^/μL (1 point), evidence of hemolysis (reticulocyte count >2.5%, undetectable haptoglobin, or indirect bilirubin >2.0 mg/dL; 1 point), active cancer within one year (1 point), history of solid organ or stem cell transplant (1 point), mean corpuscular volume <90 fL (1 point), international normalized ratio <1.5 (1 point), and serum creatinine <2.0 mg/dL (1 point). Patients were categorized as high-risk (score 6–7), intermediate-risk (score 5), or low-risk (score 0–4) according to validated thresholds [9,21].

For patients with Guillain–Barré syndrome, the Hughes functional grading scale was assessed at admission, weekly during hospitalization, and at discharge by board-certified neurologists. The scale ranges from 0 (healthy) to 6 (death), with scores of 4 (bedridden) and 5 (mechanical ventilation) indicating severe disability [22]. Change in Hughes score from admission to discharge (ΔHughes) was calculated as a continuous measure of functional recovery.

For patients with myasthenia gravis crisis, the Myasthenia Gravis Foundation of America post-intervention status was assessed at discharge and categorized as improved, unchanged, worse, exacerbation, or death [10]. Quantitative Myasthenia Gravis scores were recorded when available.

Volume status assessment was performed systematically in all patients with renal indications. Daily weight measurements were obtained using calibrated digital scales, and strict fluid balance documentation was maintained throughout the hospitalization. Point-of-care ultrasound of the inferior vena cava and lung fields was performed when clinically indicated using a portable ultrasound system (GE Vscan Extend, Chicago, IL, USA). Inferior vena cava collapsibility index was measured during quiet respiration, with values <40% suggestive of hypervolemia and >60% indicating hypovolemia. A bilateral lung ultrasound was performed to detect B-lines (≥3 per field) suggestive of pulmonary congestion. These findings supported fluid management decisions and replacement fluid selection.

For patients with systemic lupus erythematosus, disease activity was quantified using the Systemic Lupus Erythematosus Disease Activity Index 2000 (SLEDAI-2K) at baseline and at discharge [11]. Scores were calculated by two independent rheumatologists, with adjudication of discrepancies by consensus.

Medication history within the 30 days preceding TPE initiation was systematically reviewed. Immunosuppressive agents, including corticosteroids, cyclophosphamide, rituximab, mycophenolate mofetil, azathioprine, and calcineurin inhibitors, were recorded with dose, route, and duration. Prior exposure to intravenous immunoglobulin was documented. Nephrotoxic and neurotoxic agents were recorded and categorized by drug class. Infection status at TPE initiation was documented using Sepsis-3 criteria; microbiological cultures, inflammatory markers, including C-reactive protein and procalcitonin when available, and chest imaging findings were recorded. Sequential Organ Failure Assessment scores were calculated at admission for critically ill patients.

### Laboratory investigations

Blood samples were collected at enrollment via venipuncture of antecubital or forearm veins using 21-gauge safety needles (BD Vacutainer, Franklin Lakes, NJ, USA) into appropriate collection tubes. For all patients, samples were obtained within 24 hours before the first TPE session to establish accurate baseline parameters. For patients transferred from other departments or referring hospitals, repeat baseline sampling was performed immediately upon admission to the apheresis unit to ensure standardization across the cohort.

Complete blood count was performed using a Sysmex XN-9000 automated hematology analyzer (Sysmex Corporation, Kobe, Japan). Parameters recorded included hemoglobin concentration (g/dL), white blood cell count (×10^3^/μL), and platelet count (×10^3^/μL). The analyzer underwent daily internal quality control and participated in external quality assurance programs through the College of American Pathologists. Routine biochemistry profiles, including serum creatinine, blood urea nitrogen, and lactate dehydrogenase, were measured using a Cobas 8000 modular analyzer (Roche Diagnostics, Basel, Switzerland) with enzymatic methods. Serum creatinine was measured using an enzymatic method calibrated to isotope dilution mass spectrometry reference standards.

For patients with suspected thrombotic thrombocytopenic purpura, ADAMTS13 activity and inhibitor titers were measured when available. Plasma was collected in 3.2% sodium citrate tubes, centrifuged at 2500 × g for 15 minutes at 4°C, stored at −80°C, and analyzed in batches using a fluorescence resonance energy transfer assay (Technozym ADAMTS-13 Activity ELISA, Technoclone, Vienna, Austria). Severe ADAMTS13 deficiency was defined as activity <10%. In the metabolic group, fasting triglycerides, total cholesterol, LDL cholesterol, and HDL cholesterol were measured by enzymatic colorimetric methods on the Cobas 8000 analyzer. In the biomarker substudy, serum NGAL and cystatin C were measured within 24 hours of TPE initiation in 34 consenting patients with thrombotic microangiopathies. Samples were centrifuged at 2500 rpm for 15 minutes at 4°C, aliquoted, and stored at −80°C until batch analysis. NGAL and cystatin C were measured using commercial ELISA kits (Sunred Biological Technology Co., Ltd., Shanghai, China) according to manufacturer protocols validated per CLSI EP15-A3 guidelines. Absorbance was measured using a BioTek ELx808 microplate reader (Agilent Technologies, Santa Clara, CA, USA). Intra- and inter-assay coefficients of variation were <10% for both assays.

All laboratory measurements were performed by trained laboratory technicians blinded to clinical data and patient outcomes. Results were automatically transmitted to the electronic medical record system and manually verified by study investigators.

### TPE procedure and devices

Therapeutic plasma exchange was performed using two devices. Membrane plasma exchange was conducted using a plasma separator (Ngl/xjc2000, Nigale Company, China) and the COM.TEC Fresenius Kabi system (Germany) with hollow-fiber plasma filters. Centrifugal plasma exchange was performed using the COM.TEC Fresenius Kabi system with centrifugal separation. Membrane TPE was the primary modality utilized in the nephrology unit; centrifugal TPE was employed when membrane filtration was contraindicated or technically challenging, such as in cases of severe hemoconcentration or difficulty achieving adequate blood flow.

Vascular access was achieved primarily through temporary non-tunneled dialysis catheters of the Mahurkar type (11.5–12 Fr, double-lumen) inserted into the internal jugular or femoral vein under sterile conditions with ultrasound guidance, which was employed in 210 patients (95.0%). The remaining 11 patients (5.0%) underwent TPE via pre-existing arteriovenous fistulas or grafts placed for chronic hemodialysis.

Anticoagulation was achieved using either unfractionated heparin or regional citrate anticoagulation. Unfractionated heparin was administered as a 50–100 IU/kg bolus followed by 500–1500 IU/hour continuous infusion, titrated to maintain activated clotting time between 180 and 220 seconds; this was the preferred method. Regional citrate anticoagulation with ACD-A at a ratio of 1:16–1:24, accompanied by calcium gluconate infusion to maintain ionized calcium above 0.9 mmol/L, was used in patients at high risk of bleeding.

Replacement fluid selection was determined by the underlying etiology in accordance with ASFA guidelines [4]. Fresh frozen plasma at a volume of 1.0 to 1.5 times the estimated plasma volume was used for thrombotic thrombocytopenic purpura, hemolytic uremic syndrome, antibody-mediated rejection, and diffuse alveolar hemorrhage. Five percent albumin at the same volume was used for Guillain–Barré syndrome, myasthenia gravis, chronic inflammatory demyelinating polyneuropathy, acute disseminated encephalomyelitis, neuromyelitis optica, hyperviscosity syndrome, cryoglobulinemia, ANCA-associated vasculitis, anti-glomerular basement membrane disease, and severe hypertriglyceridemia.

The choice between fresh frozen plasma and albumin as replacement fluid was guided by the specific pathogenic mechanism and clinical context of each indication. Fresh frozen plasma was preferentially used when replacement of deficient plasma components, such as ADAMTS13 in thrombotic thrombocytopenic purpura, coagulation factors in active bleeding, or complement regulators in hemolytic uremic syndrome, was therapeutically essential. Conversely, 5% albumin was selected for conditions where the primary goal was antibody or immune complex removal without the need for factor replacement, thereby avoiding the risks of transfusion-transmitted infection, allergic reactions, and volume overload associated with FFP. This approach aligns with the 2023 ASFA guidelines, which specify replacement fluid based on disease-specific rationale rather than institutional preference or availability [4].

Estimated plasma volume was calculated using Kaplan’s equation: estimated plasma volume in liters equals 0.07 multiplied by weight in kilograms multiplied by one minus hematocrit, where hematocrit is expressed as a decimal fraction [2]. The target exchange volume was 1.0 to 1.5 times the estimated plasma volume per session. Blood flow rate was maintained at 100–150 mL/min for membrane TPE and 50–80 mL/min for centrifugal TPE. Treatment frequency was daily or alternate-day, depending on disease acuity. Transmembrane pressure was maintained below 500 mmHg to prevent hemolysis. Mean session duration was 120 ± 35 minutes.

### Outcome measures

The primary outcomes were clinical response and all-cause mortality. Clinical response was assessed at 30 days post-TPE initiation or at hospital discharge, whichever occurred first, and was categorized as complete remission, partial remission, or no response. A composite response variable (complete or partial remission vs. no response) was created to enable multivariate modeling across the heterogeneous cohort. To preserve clinical interpretability, disease-specific response criteria were defined a priori for each indication (detailed below), disease-specific outcomes are reported separately in **Table 5**, and the composite variable was used only for pooled multivariate analyses **(Table 10).** Complete remission was defined as resolution of all clinical signs and symptoms attributable to the underlying condition with normalization or return to baseline of relevant laboratory parameters. For thrombotic thrombocytopenic purpura, this required platelet count exceeding 150 × 10^3^/μL; for autoimmune hemolytic anemia, hemoglobin stabilization without transfusion; for hypertriglyceridemia, triglyceride level below 500 mg/dL; for Guillain–Barré syndrome, improvement of at least two points on the Hughes disability scale; and for myasthenia gravis, Myasthenia Gravis Foundation of America post-intervention status of “improved” or “minimal manifestations.” Partial remission was defined as substantial clinical improvement with at least 50% reduction in disease-specific severity score or laboratory abnormality without meeting complete remission criteria. No response was defined as the absence of clinical improvement, disease progression, or requirement for rescue therapy, including intravenous immunoglobulin, rituximab, or cyclophosphamide. All-cause mortality was assessed at 30 days, 90 days, and at the end of follow-up, defined as hospital discharge or December 31, 2022.

Secondary outcomes included adverse events, ASFA guideline adherence rate, length of hospital stay, intensive care unit admission rate, duration of mechanical ventilation, and disease-specific outcome measures. Adverse events occurring during or between TPE sessions were recorded in real-time in the institutional database and classified according to the Common Terminology Criteria for Adverse Events (CTCAE) version 5.0 and the World Apheresis Association registry classification [21]. Complication rates are reported as the proportion of patients experiencing at least one event of a given type during their treatment course. For intra-procedural events (hypotension, allergic reactions, muscle cramps), the proportion of total sessions affected is also reported to provide procedural safety context. Events recorded included hypotension defined as systolic blood pressure below 90 mmHg or a decrease exceeding 40 mmHg requiring intervention, allergic reactions ranging from urticaria to anaphylaxis, muscle cramps, catheter-related complications including infection, thrombosis, bleeding, and hematoma, citrate toxicity manifesting as hypocalcemia, paresthesia, or tetany, transfusion-related acute lung injury, transfusion-associated circulatory overload, and coagulopathy defined as fibrinogen below 150 mg/dL post-TPE. ASFA guideline adherence rate was calculated as the proportion of TPE procedures performed for ASFA category I or II indications. Disease-specific outcome measures included time to platelet count normalization and PLASMIC score correlation for thrombotic thrombocytopenic purpura, change in Hughes disability scale from admission to discharge for Guillain–Barré syndrome, Myasthenia Gravis Foundation of America post-intervention status for myasthenia gravis, SLEDAI-2K score change for systemic lupus erythematosus, and dialysis independence at discharge for renal indications.

Disease-specific outcome measures were defined as follows. For thrombotic thrombocytopenic purpura, time to platelet count normalization was defined as the number of days from the first TPE session to the first of two consecutive platelet counts >150 × 10^3^/μL, reported as median with interquartile range and compared between PLASMIC score strata using the log-rank test; PLASMIC score correlation with clinical outcomes was also assessed. For Guillain–Barré syndrome, the change in Hughes disability scale from admission to discharge (ΔHughes) was calculated as a continuous measure of functional recovery. For myasthenia gravis, the Myasthenia Gravis Foundation of America post-intervention status was recorded at discharge. For systemic lupus erythematosus, SLEDAI-2K score change was assessed in patients with paired assessments at baseline and discharge, with the mean reduction reported with 95% confidence intervals. For renal indications, dialysis independence at discharge was defined as freedom from renal replacement therapy for ≥7 consecutive days before hospital discharge, reported as a proportion among patients requiring dialysis at TPE initiation.

For the NGAL and cystatin C biomarker substudy, serum samples from 34 consenting patients with thrombotic microangiopathies were analyzed in duplicate; the association between biomarker concentrations and time to platelet recovery was assessed using Cox regression, with optimal cut-off values identified by receiver operating characteristic curve analysis.

### Quality assurance and bias mitigation

Data extraction was performed independently by two investigators using a standardized electronic case report form. Discrepancies were resolved by consensus with a third senior investigator. Clinical response and adverse event classification were adjudicated by a blinded committee comprising one nephrologist, one hematologist, and one neurologist, none of whom were involved in the patients’ clinical care. Selection bias was minimized by the consecutive enrollment of all eligible patients over the seven years. Information bias was reduced by the use of real-time documented standardized apheresis logs and laboratory data obtained from automated analyzers with regular calibration.

### Disease-specific response criteria definition

Clinical response was assessed using pre-specified, disease-specific criteria defined a priori for each indication. Complete remission required: (1) for thrombotic thrombocytopenic purpura: platelet count >150 × 10^3^/μL with resolution of microangiopathic hemolytic anemia (normal LDH, schistocytes <1%) and no new end-organ damage; (2) for autoimmune hemolytic anemia: hemoglobin stabilization ≥10 g/dL without transfusion for 7 days with normalization of LDH; (3) for hyperviscosity syndrome: resolution of mucocutaneous bleeding, visual disturbances, and neurological symptoms with serum viscosity <3.0 cP; (4) for Guillain–Barré syndrome: Hughes disability score ≤2 (able to walk independently) with improvement in at least two functional grades; (5) for myasthenia gravis crisis: Myasthenia Gravis Foundation of America post-intervention status of “improved” or “minimal manifestations” with successful extubation; (6) for acute disseminated encephalomyelitis: complete neurological recovery with normal or stable/resolving MRI findings; (7) for neuromyelitis optica: complete visual and motor recovery with no relapses during hospitalization; (8) for chronic inflammatory demyelinating polyneuropathy: Hughes score ≤2 with return to baseline functional status; (9) for transverse myelitis: complete motor and sensory recovery with independent ambulation; (10) for progressive multifocal leukoencephalopathy: neurological stabilization with stable/improving MRI lesions; (11) for antibody-mediated rejection: return to baseline creatinine with no further increase in donor-specific antibodies; (12) for pre-kidney transplant desensitization: successful transplantation with negative crossmatch or ≥50% reduction in panel reactive antibody titers; (13) for catastrophic antiphospholipid syndrome: resolution of microangiopathy with no new thrombotic events; (14) for SLE with neurological involvement: SLEDAI-2K score ≤4 with resolution of neuropsychiatric symptoms; (15) for diffuse alveolar hemorrhage: resolution of hemoptysis, stable/improving oxygenation, and radiographic clearance; and (16) for severe hypertriglyceridemia: triglycerides <500 mg/dL. Partial remission was defined as ≥50% improvement in the respective disease-specific parameter without achieving complete remission criteria. No response was defined as the absence of clinical improvement, disease progression, or requirement for rescue immunosuppressive therapy.

### PLASMIC score protocol and risk stratification

For all patients with suspected thrombotic thrombocytopenic purpura, the PLASMIC score was calculated by two independent investigators using the validated seven-component scoring system: platelet count <30 × 10^3^/μL (1 point), evidence of hemolysis (reticulocyte count >2.5%, undetectable haptoglobin, or indirect bilirubin >2.0 mg/dL; 1 point), active cancer within one year (1 point), history of solid organ or stem cell transplant (1 point), mean corpuscular volume <90 fL (1 point), international normalized ratio <1.5 (1 point), and serum creatinine <2.0 mg/dL (1 point). Total scores were stratified into high-risk (6–7 points), intermediate-risk (5 points), and low-risk (0–4 points) categories according to published validated thresholds [22]. Due to resource limitations and the retrospective study period (the PLASMIC score was calculated from existing medical records), ADAMTS13 activity testing was available in only 34 of 54 TTP patients (63.0%); samples were collected in 3.2% sodium citrate tubes, centrifuged at 2500 × g for 15 minutes at 4°C, stored at −80°C, and analyzed in batches using a fluorescence resonance energy transfer assay (Technozym ADAMTS-13 Activity ELISA, Technoclone, Vienna, Austria). Severe deficiency was defined as activity <10%.

### Hughes scale and poor outcome definition

Neurological disability in Guillain–Barré syndrome patients was serially assessed using the Hughes functional grading scale: grade 0 (healthy), grade 1 (minor symptoms, able to run), grade 2 (able to walk 5 meters independently but unable to run), grade 3 (able to walk 5 meters with aid), grade 4 (bedridden or chair-bound), grade 5 (requiring mechanical ventilation), and grade 6 (death). Poor functional outcome was defined a priori as Hughes disability score ≥4 at discharge (bedridden or requiring mechanical ventilation). This stricter definition was chosen to avoid circularity with the predictor (admission Hughes ≥4) and to capture patients who failed to achieve meaningful functional recovery.

### Adverse event classification and grading

Adverse events occurring during or between TPE sessions were prospectively recorded and classified according to the Common Terminology Criteria for Adverse Events (CTCAE) version 5.0. Events were graded on a 1–5 scale: grade 1 (mild; asymptomatic or mild symptoms, intervention not indicated), grade 2 (moderate; minimal, local, or non-invasive intervention indicated), grade 3 (severe or medically significant but not immediately life-threatening; hospitalization or prolongation of hospitalization indicated), grade 4 (life-threatening consequences; urgent intervention indicated), and grade 5 (death related to adverse event).

Events were further categorized by timing: intra-procedural events occurring during the TPE session, and inter-session events occurring between sessions during the same hospitalization. Events were also classified as procedure-related (directly attributable to vascular access, anticoagulation, or the apheresis procedure itself), disease-related (attributable to the underlying condition or its complications), or transfusion-related (attributable to replacement fluid, either FFP or albumin).

For analysis, clinically significant adverse events were defined as CTCAE grade ≥2. Grade 1 events were recorded but not included in complication rate analyses unless they required intervention or prolonged hospitalization. This classification system follows World Apheresis Association registry standards [21].

### Statistical analysis

All statistical analyses were performed using IBM SPSS Statistics for Windows, Version 27.0 (IBM Corp., Armonk, NY) and R statistical software, Version 4.2.2 (R Foundation for Statistical Computing, Vienna, Austria) with the following packages: tidyverse, survival, survminer, rms, mice, and pROC.

Descriptive statistics were computed for all variables. Continuous variables were tested for normality using the Shapiro–Wilk test and Q-Q plots. Normally distributed variables were reported as mean ± standard deviation and compared using an independent samples t-test for two-group comparisons and one-way analysis of variance with post-hoc Tukey honest significant difference test for comparisons among three or more groups. Non-normally distributed variables were reported as median with interquartile range and compared using the Mann–Whitney U test for two-group comparisons and the Kruskal–Wallis test with post-hoc Dunn’s test for comparisons among three or more groups. Categorical variables were reported as frequencies and percentages and compared using Pearson’s chi-square test or Fisher’s exact test when the expected cell counts were less than five. Between-group comparisons among the four diagnostic groups (renal, hematologic, neurologic, and metabolic) were performed using analysis of variance or the Kruskal–Wallis test for continuous variables and the chi-square test for categorical variables. Pairwise post-hoc comparisons were adjusted using Bonferroni correction for multiple testing.

Survival analysis was performed using the Kaplan–Meier method to estimate overall survival. Comparison of survival distributions between groups was conducted using the log-rank test. Survival curves were truncated at 90 days of follow-up to minimize confounding from non-TPE-related late mortality. Univariate logistic regression was performed to identify candidate predictors of mortality and clinical response. The complete list of candidate variables entered into univariate logistic regression for mortality prediction included: age (continuous and >60 years), sex, diagnostic group (renal, hematologic, neurologic, metabolic), ASFA category (I, II, III), mechanical ventilation requirement (yes/no), baseline hemoglobin (continuous and <8 g/dL), baseline platelet count (continuous and <50 × 10^3^/μL), baseline serum creatinine (continuous and >2.5 mg/dL), infection during TPE course (yes/no), hypotension during TPE session (yes/no), number of TPE sessions (continuous and <5), replacement fluid type (FFP vs. albumin), and vascular access type (catheter vs. fistula). Variables with *p* < 0.10 in univariate analysis were entered into multivariate logistic regression models using the enter method (forced entry of all candidate variables), as this approach preserves the pre-specified analytic framework and avoids data-driven variable selection [20]. Backward stepwise selection was not used for the primary mortality model. Multicollinearity among predictors was assessed using variance inflation factors (VIF); all VIF values were <2, indicating no concerning collinearity. Model fit was assessed using the Hosmer-Lemeshow goodness-of-fit test, with p > 0.05 indicating adequate fit, and Nagelkerke R^2^. The mortality model demonstrated adequate calibration (Hosmer-Lemeshow *p* = 0.62; Nagelkerke R^2^ = 0.41), and the response model demonstrated adequate calibration (Hosmer-Lemeshow *p* = 0.34; Nagelkerke R^2^ = 0.38). To identify independent predictors of clinical response (complete/partial remission vs. no response), the same multivariate approach was applied using the identical candidate variable set, with clinical response as the outcome. Results were reported as adjusted odds ratios with 95% confidence intervals and corresponding p-values. Multicollinearity among predictors was assessed using variance inflation factors (VIF); all VIF values were <2, indicating no concerning collinearity. Model fit was assessed using the Hosmer–Lemeshow goodness-of-fit test, with *p* > 0.05 indicating adequate fit, and Nagelkerke R^2^.

Several advanced analyses were pre-specified as hypothesis-generating. To visualize indication-specific mortality risk, univariate logistic regression was performed with pre-kidney transplant desensitization selected as the reference category due to its observed 0% mortality and stable clinical course. Due to the presence of zero mortality in the reference category and several comparison groups, Firth penalized likelihood logistic regression was used to obtain finite odds ratio estimates and confidence intervals, thereby reducing small-sample bias [23,24]. Odds ratios with 95% confidence intervals were calculated for each indication with ≥3 patients and plotted on a logarithmic scale using the forestplot package in R. No adjustment for multiple comparisons was performed, as this analysis was explicitly hypothesis-generating and descriptive rather than confirmatory. Cox proportional hazards regression was performed for time-to-event analysis of mortality. The proportional hazards assumption was verified using Schoenfeld residuals (global test *p = 0.18*) and visual inspection of log-minus-log plots, confirming that the proportional hazards assumption was not violated. Variables entered into the Cox model included age, sex, diagnostic group, ASFA category, baseline hemoglobin, platelet count, serum creatinine, mechanical ventilation requirement, and number of TPE sessions. Results were reported as hazard ratios with 95% confidence intervals. Survival time was calculated from the date of the first TPE session to the date of death or last follow-up, with censoring at 90 days for patients alive beyond this time point, consistent with the primary 90-day mortality endpoint. Patients alive at the end of follow-up were censored.

Propensity score matching was employed to adjust for confounding in two comparisons: (1) fresh frozen plasma versus albumin as replacement fluid on mortality and response, and (2) temporary catheter versus arteriovenous fistula/shunt access on complication rates. One-to-one nearest neighbor matching was performed with a caliper width of 0.2 times the standard deviation of the logit propensity score. For the catheter versus fistula comparison, propensity scores were estimated using logistic regression with the following covariates: age, sex, diagnostic group, ASFA category, number of TPE sessions, and baseline serum creatinine. Standardized differences were calculated to assess covariate balance before and after matching, with values <0.10 indicating adequate balance. In addition to propensity score matching, inverse probability of treatment weighting (IPTW) was employed as a sensitivity analysis for the comparison of replacement fluid types. IPTW uses propensity scores as weights to create a pseudo-population in which covariates are balanced between groups, preserving the full sample size while adjusting for confounding. The same propensity score model (age, sex, diagnostic group, ASFA category, hemoglobin, platelet count, and creatinine) was used to generate weights. Balance after weighting was assessed using standardized differences, with values <0.10 indicating adequate balance. Outcomes compared included overall complication rates, catheter-related bloodstream infections, insertion-site bleeding, access thrombosis, allergic reactions, and hypotension. Subgroup and interaction analyses were conducted for pre-specified subgroups defined by diagnostic group, age stratified at 60 years, and ASFA category. Tests for interaction were performed using likelihood ratio tests comparing models with and without interaction terms. Receiver operating characteristic curve analysis was performed to identify optimal cut-off values for continuous predictors associated with mortality, including hemoglobin, creatinine, and platelet count. Area under the curve was reported with 95% confidence intervals, and Youden’s index was used for cut-point selection. To assess internal validity and correct for optimism bias, bootstrap resampling with 1,000 replicates was performed for each ROC analysis, yielding optimism-corrected AUCs.

Disease-specific multivariate models were constructed using the enter method (all variables entered simultaneously). Candidate variables were selected based on three criteria: (1) published literature and established prognostic factors; (2) clinical plausibility and biological rationale; and (3) univariate screening with *p* < 0.20. For the thrombotic thrombocytopenic purpura complete remission model, candidate variables included: PLASMIC score ≥6, time to TPE initiation ≤2 days, ADAMTS13 activity <10%, platelet count <20 × 10^3^/μL, serum creatinine >2.0 mg/dL, age > 60 years, and female sex. For the Guillain–Barré syndrome poor functional outcome model (defined as Hughes disability score ≥4 at discharge, i.e., bedridden or requiring mechanical ventilation), candidate variables included: Hughes score at admission ≥4, time to TPE initiation >7 days, age > 50 years, axonal electrophysiologic variant, male sex, and mechanical ventilation requirement. This stricter outcome definition was chosen to avoid circularity with the predictor (admission Hughes ≥4) and to capture patients who failed to achieve meaningful functional recovery (independent ambulation or ventilator liberation). A sensitivity analysis using the original threshold of ≥3 yielded consistent results (data not shown), confirming robustness. For the systemic lupus erythematosus mortality model, candidate variables included: presence of diffuse alveolar hemorrhage, catastrophic antiphospholipid syndrome, lupus cerebritis, mechanical ventilation requirement, serum creatinine >2.5 mg/dL, and hemoglobin <8 g/dL.The three-tier mortality risk stratification model was developed post-hoc following completion of primary data analysis. This model represents a hypothesis-generating synthesis of findings from multivariate logistic regression (independent predictors), Kaplan–Meier survival analysis (90-day survival probabilities), receiver operating characteristic curve analysis (optimal cut-off values), and the indication-specific forest plot (unadjusted odds ratios). The model is presented as an exploratory framework requiring prospective validation and should not be interpreted as a validated prediction tool. All primary analyses (multivariate logistic regression using the enter method, Cox proportional hazards models, and Kaplan–Meier survival analysis) were pre-specified before data extraction and analysis. To assess the robustness of predictor selection and address overfitting concerns given the events-per-variable ratio of 6.4, a sensitivity analysis using least absolute shrinkage and selection operator (LASSO) regression with 10-fold cross-validation was performed. LASSO selects predictors by shrinking coefficients and setting some to zero, reducing overfitting risk in exploratory analyses. All candidate variables entered into the LASSO regression were binary (presence/absence or above/below a pre-specified threshold), making coefficients directly comparable without standardization. Variables selected by LASSO were compared with those identified in the enter method logistic regression. The concordance between LASSO and the primary enter method logistic regression supports the stability of the five-predictor mortality model in this dataset.

Overall, missingness in the dataset was 3.7%, ranging from 0 to 12% per variable. Variables with greater than 5% missingness included components of the PLASMIC score (12%), ADAMTS13 activity (11%), and lipid profile in the metabolic group (9%). Mortality status was complete for all 221 patients, including 32 patients who died during follow-up; no missingness occurred for the primary outcome. Multiple imputation by chained equations was performed with 20 imputed datasets and 10 iterations, using predictive mean matching for continuous variables and logistic regression for categorical variables. Pooled estimates from imputed datasets were calculated using Rubin’s rules. Complete-case analysis is presented as the primary analysis; sensitivity analyses confirmed robustness of findings across both approaches. Complication rates are reported both at the patient level (proportion of patients experiencing at least one event of a given type during their treatment course) and at the session level (events per total number of TPE sessions performed). Patient-level rates are used for baseline comparisons, subgroup analyses, and risk factor modeling, as they reflect the clinically relevant outcome of whether a patient experienced any complication. Session-level rates are provided to contextualize procedural safety and allow comparison with per-procedure complication rates reported in the literature. All percentages are clearly labeled in the text and tables to indicate the denominator used.

A two-tailed p-value ≤0.05 was considered statistically significant, and *p* ≤ 0.01 was considered highly significant. All p-values were reported exactly except when *p* < 0.001. Post-hoc power analysis was performed using G*Power software (version 3.1). With 221 patients and 32 mortality events (14.5% event rate), the study had 80% power at α = 0.05 to detect predictors with an odds ratio ≥2.0 for predictors present in at least 20% of the cohort, indicating adequate sample size for the primary multivariable analyses.

## Results

**1. Cohort Demographics and Baseline Clinical Profile**

A total of 221 patients who underwent therapeutic plasma exchange at Zagazig University Hospitals between January 2016 and December 2022 were included in the final analysis. Among these, 32 patients (14.5%) died during the 90-day follow-up period. These deceased patients were included in the analysis, and no data were excluded due to death (consistent with the IRB-approved waiver of consent). **Table 1** summarizes the baseline demographic and clinical characteristics of the entire cohort.

**Table 1:** Baseline Demographic and Clinical Characteristics of 221 Patients Undergoing TPE.

Characteristic	Value
Age, years	36.0 ± 13.6
Sex, n (%)	
Male	128 (57.9)
Female	93 (42.1)
Body mass index, kg/m²	26.1 ± 3.3
Comorbidities, n (%)	
None	142 (65.1)
Hypertension	45 (20.6)
Diabetes mellitus	15 (6.8)
Chronic kidney disease	12 (5.5)
Hypothyroidism	1 (0.4)
Hyperthyroidism	1 (0.4)
Multiple myeloma	1 (0.4)
B-cell lymphoma	1 (0.4)
Laboratory parameters	
Hemoglobin, g/dL	10.8 ± 4.2
White blood cell count, × 10³/μL	10.9 ± 5.5
Platelet count, × 10³/μL	182.3 ± 117.7
Serum creatinine, mg/dL	1.8 ± 2.3
Blood urea nitrogen, mg/dL	31.3 ± 30.1

Data are presented as mean ± standard deviation unless otherwise indicated.

The mean age was 36.0 ± 13.6 years (range 18–76 years), with a male predominance (57.9%, n = 128). The mean body mass index was 26.1 ± 3.3 kg/m^2^, placing the average patient in the overweight category. Regarding comorbid conditions, 65.1% of patients (n = 142) had no documented comorbidities. Among those with comorbidities, hypertension was the most prevalent (20.6%, n = 45), followed by diabetes mellitus (6.8%, n = 15) and chronic kidney disease (5.5%, n = 12). Other comorbid conditions, including thyroid disorders, multiple myeloma, and B-cell lymphoma, each accounted for less than 1% of the cohort.

Baseline laboratory parameters demonstrated considerable heterogeneity across the cohort, reflecting the diverse underlying pathologies. The mean hemoglobin concentration was 10.8 ± 4.2 g/dL, with a median of 10.5 g/dL, indicating a substantial burden of anemia at presentation. Platelet count showed marked variability (mean 182.3 ± 117.7 × 10^3^/μL; median 200 × 10^3^/μL), with the lowest values observed in patients with thrombotic thrombocytopenic purpura. Renal function parameters were elevated overall (mean creatinine 1.8 ± 2.3 mg/dL; mean urea 31.3 ± 30.1 mg/dL), consistent with the substantial proportion of patients with renal indications (40.3%).

**2. Indications for Therapeutic Plasma Exchange and Adherence to ASFA Guidelines**

The distribution of clinical indications for therapeutic plasma exchange among the 221 patients is presented in **Table 2**. Neurologic indications were the most frequent, accounting for 51.6% of the cohort, followed by renal indications (40.3%), hematologic indications (5.9%), and metabolic indications (2.2%). Guillain–Barré syndrome and thrombotic thrombocytopenic purpura were the two most common individual indications, each comprising 24.4% of patients. Myasthenia gravis crisis represented the third most frequent indication (13.5%), followed by acute disseminated encephalomyelitis (5.4%), pre-kidney transplant desensitization (4.5%), and hyperviscosity syndrome (4.5%). Less common indications included diffuse alveolar hemorrhage (4.0%), antibody-mediated kidney transplant rejection (3.1%), catastrophic antiphospholipid syndrome (3.1%), systemic lupus erythematosus with neurological involvement (2.2%), neuromyelitis optica (2.2%), and severe hypertriglyceridemia (2.2%). Rare indications, each accounting for 1.8% or less, included myasthenia gravis preoperative preparation, chronic inflammatory demyelinating polyneuropathy, transverse myelitis, Rh incompatibility with recurrent abortion, and progressive multifocal leukoencephalopathy.

**Table 2:** Indications for Therapeutic Plasma Exchange, ASFA Category Distribution, and Procedural Characteristics.

Characteristic	N (221)	%
Diagnostic Group		
Renal (Group I)	89	40.3
Hematologic (Group II)	13	5.9
Neurologic (Group III)	114	51.6
Metabolic (Group IV)	5	2.2
Specific Indications		
Guillain–Barré syndrome	54	24.4
Thrombotic thrombocytopenic purpura	54	24.4
Myasthenia gravis crisis	30	13.5
Acute disseminated encephalomyelitis	12	5.4
Pre-kidney transplant desensitization	10	4.5
Hyperviscosity syndrome	10	4.5
Diffuse alveolar hemorrhage	9	4.0
Antibody-mediated kidney transplant rejection	7	3.1
Catastrophic antiphospholipid syndrome	7	3.1
SLE with neurological involvement	5	2.2
Neuromyelitis optica	5	2.2
Severe hypertriglyceridemia	5	2.2
Myasthenia gravis preoperative preparation	4	1.8
Chronic Inflammatory Demyelinating Polyneuropathy	3	1.3
Rh incompatibility with recurrent abortion	2	0.9
Transverse myelitis	2	0.9
Progressive multifocal leukoencephalopathy	2	0.9
ASFA Category		
Category I	172	77.8
Category II	32	14.5
Category III	17	7.7
Vascular Access		
Temporary catheter	210	95.0
Arteriovenous fistula/shunt	11	5.0
Concomitant Immunosuppression		
None	204	92.3
Steroid + cyclophosphamide	12	5.4
Steroid + rituximab	4	1.8
Steroid monotherapy	1	0.5

Adherence to ASFA guideline recommendations was excellent. The majority of procedures (77.8%) were performed for category I indications, for which TPE is accepted as first-line therapy. Category II indications accounted for 14.5% and category III indications for 7.7% of procedures, with no category IV indications performed. Clinical response rates varied across ASFA categories: 81.4% of patients with category I indications responded to therapy, compared to 68.8% for category II and 64.7% for category III (*p* = 0.03 by chi-square test for trend). This gradient of response supports the validity of the ASFA categorization framework in our cohort.

Vascular access was achieved via temporary non-tunneled catheters in 95.0% of patients, reflecting the acute nature of most indications and the absence of pre-existing permanent access. The remaining 5.0% underwent TPE via pre-existing arteriovenous fistulas or grafts, all of whom were established hemodialysis patients. Concomitant immunosuppressive therapy was administered in only 7.7% of patients, with corticosteroid-cyclophosphamide combinations being the most frequent regimen (5.4%), followed by corticosteroid-rituximab combinations (1.8%) and corticosteroid monotherapy (0.5%). This low rate of concurrent immunosuppression reflects both the therapeutic mechanism of TPE as a stand-alone therapy for certain indications and the resource constraints limiting access to costly immunomodulatory agents in our setting.

A total of 23 patients with systemic lupus erythematosus (SLE) were distributed across multiple indication categories, as detailed in Table 7. Thrombotic thrombocytopenic purpura (TTP) patients (n = 54 total) were classified by predominant presentation (renal vs. hematologic), as explained in the Study Population section. All 54 TTP patients were analyzed as a unified cohort for disease-specific outcomes **(Table 5)**. All five patients with severe hypertriglyceridemia (metabolic group) were treated during 2016–2022 and classified as ASFA Category III per guidelines in effect at that time. The 2023 ASFA guidelines have since upgraded this indication to Category II [4]. SLE: systemic lupus erythematosus; TTP: thrombotic thrombocytopenic purpura; ASFA: American Society for Apheresis.

**3. Distinct Clinical and Laboratory Phenotypes Across Diagnostic Groups**

Significant differences in baseline demographic and laboratory parameters were observed across the four diagnostic groups, as detailed in Table 3. Age distribution differed significantly, with hematologic patients being the oldest and metabolic patients the youngest. Post-hoc analysis confirmed significant age differences between the renal and hematologic groups and between the hematologic and neurologic groups. Body mass index also varied significantly across groups; hematologic patients had the highest mean BMI, while metabolic patients had the lowest. Significant pairwise differences were observed between the renal and hematologic groups and between the hematologic and metabolic groups.

**Table 3:** Baseline Demographic, Laboratory, and Procedural Characteristics by Diagnostic Group.

Characteristic	Renal (I) (n = 89)	Hematologic (II) (n = 13)	Neurologic (III) (n = 114)	Metabolic (IV) (n = 5)	*p*-value
Age, years	33.6 ± 11.6	48.0 ± 15.8	37.3 ± 14.3	26.0 ± 6.9	0.001
Male sex, n (%)	57 (64.0)	7 (53.8)	59 (51.8)	2 (40.0)	0.279
BMI, kg/m²	25.8 ± 3.2	28.1 ± 2.5	26.2 ± 3.5	22.1 ± 1.5	0.012
Hemoglobin, g/dL	8.2 ± 2.0	8.7 ± 2.1	13.1 ± 1.9	9.9 ± 2.5	<0.001
WBC, × 10³/μL	11.4 ± 7.1	5.7 ± 2.1	11.5 ± 3.6	13.1 ± 8.9	0.002
Platelets, × 10³/μL	95.9 ± 107.4	158.1 ± 75.8	244.2 ± 30.4	284 ± 72.1	<0.001
Creatinine, mg/dL	2.9 ± 2.7	2.4 ± 4.4	0.85 ± 0.78	2.8 ± 2.8	<0.001
Urea, mg/dL	46.3 ± 38.1	34.3 ± 42.8	18.7 ± 11.8	28.6 ± 21.3	<0.001
ASFA Category, n (%)					<0.001
Category I	73 (82.0)	10 (76.9)	94 (82.5)	0 (0)	
Category II	15 (16.8)	1 (7.7)	15 (13.2)	0 (0)	
Category III	1 (1.2)	2 (15.4)	5 (4.4)	5 (100)	
Number of TPE sessions	8.2 ± 3.9	5.8 ± 4.5	5.6 ± 2.0	7.8 ± 2.8	<0.001

Data are presented as mean ± standard deviation unless otherwise indicated. BMI: body mass index; WBC: white blood cell count; ASFA: American Society for Apheresis. p-values from ANOVA/Kruskal-Wallis tests for continuous variables and chi-square test for categorical variables. Severe hypertriglyceridemia (n = 5) was classified as ASFA Category III based on the 7th and 8th Edition guidelines (2016–2019, 2019–2022) in effect during the study period. The 9th Edition (2023) reclassified this indication to Category II. TTP patients with predominant renal involvement (n = 41) are included in the renal group (Group I).

Hematologic parameters demonstrated highly significant between-group differences. Hemoglobin was substantially lower in renal and hematologic patients compared to neurologic patients. Platelet count showed the most dramatic variation: the 41 patients with thrombotic thrombocytopenic purpura who were classified under renal indications (due to predominant renal involvement) had profound thrombocytopenia, contributing to the low mean platelet count in the renal group (95.9 ± 107.4 × 10^3^/μL). In contrast, neurologic patients had normal platelet counts (244.2 ± 30.4 × 10^3^/μL). These differences were highly significant across all pairwise comparisons. Renal function parameters also differed significantly; patients with renal indications had the highest creatinine and urea levels, while neurologic patients had normal renal function. These findings validate the diagnostic group stratification and reflect the expected laboratory profiles of each disease category.

Sex distribution was homogeneous across groups, although female predominance was observed in the metabolic group, with near-equal distribution in neurologic and hematologic groups. ASFA category distribution differed significantly across groups; all metabolic patients were category III, reflecting the evolving evidence base for TPE in hypertriglyceridemia. In contrast, renal, hematologic, and neurologic groups had high proportions of category I indications. The number of TPE sessions varied significantly by diagnosis; renal patients required the most intensive treatment, reflecting the standard protocol for thrombotic thrombocytopenic purpura and antibody-mediated rejection, while neurologic and hematologic patients required fewer sessions. Pairwise comparisons confirmed significantly higher session numbers in renal patients compared to both hematologic and neurologic groups.

**4. Overall Efficacy and Safety of Therapeutic Plasma Exchange**

The overall clinical outcomes and adverse event profile for the entire cohort are presented in **Table 4**. More than three-quarters of patients demonstrated a clinical response to therapeutic plasma exchange, with the vast majority of responders achieving complete remission. Only a small proportion of patients showed no response to therapy, and all-cause mortality was 14.5%.

**Table 4:** Overall Clinical Outcomes and Adverse Event Profile (N = 221 patients, 1,105 sessions).

Outcome	n	%
Clinical Response		
Responders (overall)	172	77.9
Complete remission*	160	93.0
Partial remission*	12	7.0
No response	17	7.6
All-cause mortality	32	14.5
		
Adverse Events – Patient-Level†		
Any adverse event	82	37.2
Hypotension	26	11.8
Allergic reaction	21	9.5
Muscle cramps	8	3.6
Infection (inter-session)	26	11.7
Catheter site bleeding	1	0.4
		
Adverse Events – Session-Level‡	**(per 1,105 sessions)**	
Sessions without complications	694	62.8
Hypotension	26	2.4
Allergic reaction	21	1.9
Muscle cramps	8	0.7

*Percentage of responders (n = 172). †Patient-level rates represent the proportion of patients (N = 221) who experienced at least one event of the specified type during their treatment course. These rates are used for baseline comparisons, subgroup analyses, and risk factor modeling. ‡Session-level rates represent events per total number of TPE sessions performed (1,105 sessions) and are provided to contextualize procedural safety and allow comparison with per-procedure complication rates reported in the literature. All adverse events were CTCAE grade 1–2 (mild to moderate). No grade 3–5 events occurred. Hypotension was transient and responsive to fluid resuscitation; allergic reactions (urticaria, flushing) responded to antihistamines; muscle cramps were self-limited requiring no intervention; infections were managed with standard antimicrobial therapy without requiring TPE discontinuation. Note: Responder status in Table 4 reflects best clinical response achieved during hospitalization. Mortality reflects all-cause death by 90 days. Three patients who achieved clinical response subsequently died before 90 days; they are included as responders in this table because their response was clinically meaningful. Table 6 reports outcomes by diagnostic group using the same definitions.

The procedure was well-tolerated overall, with the majority of sessions (62.8%) completed without any adverse events. Considering patient-level outcomes, 37.2% of patients experienced at least one adverse event during their treatment course. Among the complications observed, hypotension was the most frequent, occurring in 11.8% of patients (26 of 221), followed by allergic reactions in 9.5% of patients (21 of 221) and muscle cramps in 3.6% of patients (8 of 221). When calculated per session, these events occurred in 2.4% of sessions for hypotension, 1.9% for allergic reactions, and 0.7% for muscle cramps, reflecting the low per-procedure risk. All documented adverse events were CTCAE grade 1–2 (mild to moderate) in severity and resolved with conservative management; no session required premature termination due to complications. No grade 3–5 adverse events occurred. Hypotensive episodes were managed with fluid resuscitation and temporary session slowing; allergic reactions responded to antihistamines and corticosteroids. All events were classified as procedure-related; no disease-related or transfusion-related serious adverse events were observed. Between treatment sessions, infections occurred in 11.7% of patients (26 of 221)); at the session level, infections occurred in 2.4% of TPE sessions (26 of 1,105 sessions), while bleeding from the dialysis catheter site was rare (0.4%, 1 patient). This infection rate, while higher than some international reports, reflects the cumulative immunosuppressive effect of repeated plasma exchange, particularly immunoglobulin depletion, in the context of severe underlying disease states.

**5. Disease-Specific Therapeutic Response: A Clinical Hierarchy**

Marked heterogeneity in therapeutic efficacy was observed across the spectrum of TPE indications, establishing a clear clinical hierarchy of response (Table 5). The highest response rates, exceeding 85%, were observed in autoimmune hemolytic anemia, hyperviscosity syndrome, myasthenia gravis crisis, and thrombotic thrombocytopenic purpura. Favorable response rates between 75% and 85% were achieved in Guillain–Barré syndrome, acute disseminated encephalomyelitis, and neuromyelitis optica. Of note, while ADEM and NMO demonstrated favorable overall response rates (75.0% and 80.0%, respectively), all responses were partial remissions; no patient achieved complete remission by the pre-specified criteria requiring complete neurological recovery with normal or stable/resolving MRI findings. This likely reflects the strict neuroimaging criteria used, as residual MRI abnormalities may persist despite clinical improvement. Modest responses, ranging from 50% to 70%, were seen in antibody-mediated kidney transplant rejection, pre-kidney transplant desensitization, and systemic lupus erythematosus with neurological involvement. Poor response rates below 50% were documented in chronic inflammatory demyelinating polyneuropathy, while transverse myelitis and progressive multifocal leukoencephalopathy demonstrated no response to therapy.

**Table 5:** Disease-specific clinical outcomes and mortality.

Indication	N	Complete Remission n (%)	Partial Remission n (%)	No Response n (%)	Mortality n (%)
Hematologic					
TTP	54	42 (77.8)*	4 (7.4) *	8 (14.8)*	8 (14.8)*
AHA	3	3 (100)	0	0	0
Hyperviscosity	9	8 (88.9)	0	0	1 (11.1)
Neurologic					
GBS	54	26 (48.1)	19 (35.2)	2 (3.7)	7 (13.0)
MG crisis	30	26 (86.7)	0	0	4 (13.3)
ADEM	12	0	9 (75.0)	1 (8.3)	2 (16.7)
NMO	5	0	4 (80.0)	1 (20.0)	0
CIDP	3	1 (33.3)	0	2 (66.7)	0
Transverse myelitis	2	0	0	2 (100)	0
Renal					
Pre-transplant desensitization	10	0	7 (70.0)	3 (30.0)	0
Antibody-mediated rejection	7	0	4 (57.1)	3 (42.9)	2 (28.6)
Systemic Lupus Erythematosus					
Lupus cerebritis(SLE with neurological involvement)	5	1 (20.0)	2 (40.0)	0	2 (40.0)
Catastrophic APS	7	1 (14.3)	3 (42.9)	0	3 (42.9)
Pulmonary					
Diffuse alveolar hemorrhage	9	0	0	0	9 (100)
Metabolic					
Severe hypertriglyceridemia	5	2 (40.0)	2 (40.0)	0	0

Mortality also varied substantially by indication. Diffuse alveolar hemorrhage carried the highest mortality, followed by antibody-mediated rejection, thrombotic thrombocytopenic purpura, and Guillain–Barré syndrome. No deaths occurred among patients with autoimmune hemolytic anemia, hyperviscosity syndrome, acute disseminated encephalomyelitis, neuromyelitis optica, chronic inflammatory demyelinating polyneuropathy, transverse myelitis, or metabolic indications.

These findings delineate a distinct therapeutic hierarchy, with the highest response rates observed in autoimmune hemolytic anemia, hyperviscosity syndrome, myasthenia gravis, and thrombotic thrombocytopenic purpura. In contrast, response rates were lowest in conditions characterized by advanced tissue destruction at the time of intervention, notably diffuse alveolar hemorrhage and progressive multifocal leukoencephalopathy.

Response and mortality are independent outcomes; patients who died are not automatically classified as ‘no response’ unless they were alive without improvement at 90 days. *Percentages calculated based on the total TTP cohort (n = 54). TTP patients were identified across diagnostic groups and analyzed together as a unified cohort for disease-specific outcomes. Of the 8 patients classified as no response, all 8 subsequently died. The 8 deaths (14.8%) represent these same patients; no patient who achieved complete or partial remission died during the 90-day follow-up period in this subgroup. The SLE-related rows in this table (catastrophic antiphospholipid syndrome, lupus cerebritis) represent cross-cutting disease subgroups analyzed from across diagnostic groups (renal, neurologic) as described in Methods. The same patients are reported in the SLE subgroup analysis (Table 7). TTP: thrombotic thrombocytopenic purpura; AHA: autoimmune hemolytic anemia; GBS: Guillain–Barré syndrome; MG: myasthenia gravis; ADEM: acute disseminated encephalomyelitis; NMO: neuromyelitis optica; CIDP: chronic inflammatory demyelinating polyneuropathy; SLE: systemic lupus erythematosus; APS: antiphospholipid syndrome.

**6. Outcomes and Complications Stratified by Diagnostic Category**

Significant differences in both therapeutic outcomes and complication profiles were observed across the four diagnostic groups, as detailed in **Table 6**. Clinical response rates differed substantially, with the hematologic group demonstrating the highest response rate, followed closely by the neurologic group and then the renal group. All five metabolic patients responded to therapy. Among responders, complete remission was achieved most frequently in the neurologic group and least frequently in the hematologic group.

**Table 6:** Clinical outcomes and adverse events by diagnostic group.

Variable	Renal (I) (n = 89)	Hematologic (II) (n = 13)	Neurologic (III) (n = 114)	Metabolic (IV) (n = 5)	*p*-value
Outcome					<0.001
Responders, n (%)	65 (73.2)	11 (84.6)	93 (81.6)	5 (100)	
Complete remission*	54 (83.1)	8 (72.7)	92 (98.9)	4 (80.0)	
Partial remission*	11 (16.9)	3 (27.3)	1 (1.1)	1 (20.0)	
No response, n (%)	5 (5.6)	0	13 (11.5)	0	
Mortality, n (%)	19 (21.3)	2 (15.4)	8 (7.0)	0	<0.001
Complications					0.001
No complications	47 (53.4)	9 (69.2)	70 (61.4)	3 (60)	
Hypotension	15 (16.7)	1 (7.7)	10 (8.8)	1 (20)	
Allergic reaction	14 (15.3)	1 (7.7)	22 (19.3)	1 (20)	
Muscle cramps	2 (2.4)	2 (15.4)	7 (6.1)	0	
Catheter bleeding	1 (1.2)	0	7 (6.1)	0	
Infection	15 (16.7)	1 (7.7)	10 (8.8)	0	
Ventilation requirement	9 (9.8)	0	1 (0.9)	0	

Mortality also varied significantly across diagnostic categories. The renal group had the highest mortality, followed by the hematologic group and the neurologic group, while no deaths occurred in the metabolic group.

Complication profiles likewise demonstrated marked between-group differences. The hematologic group had the highest proportion of complication-free sessions, whereas the renal group had the lowest. Hypotension was more frequent in renal and metabolic patients, while allergic reactions were more common in neurologic patients. Catheter-related bleeding occurred exclusively in renal and neurologic groups. Infection as an inter-session complication was most prevalent in the renal group, reflecting the cumulative immunosuppressive effect of the more intensive treatment regimens required for this population.

Percentage of responders within each group. Mortality represents all-cause 90-day mortality and may overlap with response categories (some patients who achieved clinical response subsequently died). Response (complete/partial remission) and mortality are presented as independent outcome measures. The “no response” category includes only patients alive at 90 days without clinical improvement. Three patients who achieved clinical response at 30 days subsequently died before 90 days; they are included as responders in this table but as mortality in Table 4. The total number of unique patients across both tables is 221.

**7. Systemic Lupus Erythematosus: Phenotype-Dependent Outcomes and Prognostic Heterogeneity**

A total of 23 patients with systemic lupus erythematosus underwent therapeutic plasma exchange for severe, refractory manifestations, as detailed in Table 7. The cohort was exclusively female, with a mean age of 30.3 years.

**Table 7:** Clinical characteristics and outcomes of patients with systemic lupus erythematosus (n = 23).

Characteristic	Value
Age, years	30.3 ± 7.3
Female sex, n (%)	23 (100)
Indications for TPE, n (%)	
Catastrophic antiphospholipid syndrome	7 (30.4)
Lupus cerebritis	5 (21.7)
Thrombotic thrombocytopenic purpura	4 (17.4)
Diffuse alveolar hemorrhage	3 (13.0)
Guillain–Barré syndrome	2 (8.7)
Progressive multifocal leukoencephalopathy	2 (8.7)
ASFA Category, n (%)	
Category I	8 (34.8)
Category II	10 (43.5)
Category III	5 (21.7)
Number of TPE sessions	5.1 ± 2.6
Overall Outcomes, n (%)	
Mortality	12 (52.2)
Improved	7 (30.4)
Complete remission	4 (17.4)
Complications, n (%)	
No complications	19 (82.6)
Fever	2 (8.7)
Ventilation requirement	1 (4.3)
Allergic reaction	1 (4.3)
Concomitant Immunosuppression, n (%)	
Steroid + cyclophosphamide	12 (52.2)
Steroid + rituximab	4 (17.4)
Steroid monotherapy	1 (4.3)
None	6 (26.1)

SLE-associated thrombotic thrombocytopenic purpura (n = 4), diffuse alveolar hemorrhage (n = 3), and Guillain–Barré syndrome (n = 2) are included within the total cohorts for these indications reported in Tables 5 and 8. All other SLE manifestations are unique to this subgroup analysis.

Indications for TPE demonstrated considerable phenotypic heterogeneity. The most common indications were catastrophic antiphospholipid syndrome, lupus cerebritis, and thrombotic thrombocytopenic purpura, followed by diffuse alveolar hemorrhage, while Guillain–Barré syndrome and progressive multifocal leukoencephalopathy each represented a smaller proportion. ASFA category distribution reflected the heterogeneous evidence base supporting TPE across different SLE manifestations, with approximately one-third of procedures performed for category I indications, just over two-fifths for category II, and the remainder for category III.

Overall mortality was 52.2%, with approximately one-third of patients showing improvement and one-sixth achieving complete remission. However, mortality varied dramatically by SLE phenotype. All patients with diffuse alveolar hemorrhage died, representing the highest mortality rate. Mortality was also substantial among patients with catastrophic antiphospholipid syndrome and lupus cerebritis. In contrast, only one-quarter of patients with SLE-associated thrombotic thrombocytopenic purpura died, and all patients with SLE-associated Guillain–Barré syndrome and progressive multifocal leukoencephalopathy survived. These findings demonstrate that the high overall mortality in this SLE cohort is driven predominantly by the inclusion of patients with diffuse alveolar hemorrhage and catastrophic antiphospholipid syndrome—both life-threatening, multi-organ manifestations—whereas patients with isolated neuropsychiatric involvement or thrombotic thrombocytopenic purpura had more favorable outcomes.

The majority of SLE patients experienced no complications. Fever, ventilation requirement, and allergic reaction were the most frequent adverse events, each occurring in a small minority. Concomitant immunosuppression was administered to nearly three-quarters of patients, predominantly steroid-cyclophosphamide combinations followed by steroid-rituximab combinations.

These findings suggest that the high overall mortality in this SLE cohort was largely attributable to the inclusion of patients with diffuse alveolar hemorrhage and catastrophic antiphospholipid syndrome, both life-threatening, multi-organ manifestations, whereas patients with isolated neuropsychiatric involvement or thrombotic thrombocytopenic purpura had more favorable outcomes. In this cohort, these findings suggest that TPE in SLE was used primarily for severe, life-threatening manifestations and that outcomes varied according to clinical phenotype. While favorable outcomes were observed in some patients with catastrophic antiphospholipid syndrome and thrombotic thrombocytopenic purpura, patients with diffuse alveolar hemorrhage, particularly those requiring mechanical ventilation, had poor outcomes. These data refine prognostic counseling for SLE patients referred for TPE.

**8. Diffuse Alveolar Hemorrhage: Uniformly Fatal Despite Intervention**

Diffuse alveolar hemorrhage represented the most severe pulmonary complication in our cohort, occurring in nine patients (Table 8). The majority were female, with a mean age of 32.7 years. Underlying etiologies included systemic lupus erythematosus, ANCA-associated vasculitis, and anti-glomerular basement membrane disease.

**Table 8:** Clinical characteristics and outcomes of patients with diffuse alveolar hemorrhage (n = 9).

Characteristic	Value
Age, years	32.7 ± 10.5
Female sex, n (%)	7 (77.8)
Body mass index, kg/m²	30.1 ± 3.4
Underlying etiology, n (%)	
Systemic lupus erythematosus*	5 (55.6)
ANCA-associated vasculitis	3 (33.3)
Anti-GBM disease	1 (11.1)
Laboratory parameters	
Hemoglobin, g/dL	7.2 ± 1.4
Platelet count, × 10³/μL	171.4 ± 60.0
Creatinine, mg/dL	4.9 ± 2.6
Urea, mg/dL	96.6 ± 79.9
ASFA Category, n (%)	
Category I	7 (77.8)
Category II	2 (22.2)
Number of TPE sessions	4.1 ± 1.9
Clinical Outcome	
Mortality, n (%)	9 (100)
Complications, n (%)	
No complications	7 (77.8)
Hypotension	1 (11.1)
Ventilation requirement†	1 (11.1)
Concomitant Immunosuppression, n (%)	
Steroid + cyclophosphamide	6 (66.7)
None	3 (33.3)

*Includes 3 patients with SLE-associated DAH also reported in Table 7. The remaining 4 patients had non-SLE etiologies (ANCA-associated vasculitis, n = 3; anti-GBM disease, n = 1). †All patients required mechanical ventilation at baseline before TPE initiation. Ventilation requirement is reported as a complication only when the duration exceeded 7 days post-TPE or when new respiratory deterioration occurred during the treatment course.

All patients with DAH were critically ill at presentation, with every patient requiring mechanical ventilation at the time of TPE initiation. Profound anemia and severely impaired renal function were consistent findings. The majority of cases were classified as ASFA category I indications, with the remainder as category II. Patients received a mean of four TPE sessions, all with fresh frozen plasma as replacement fluid, and two-thirds received concomitant immunosuppression with steroid-cyclophosphamide.

Despite intervention, in-hospital mortality was 100%. All nine patients died during their hospitalization. Complications during treatment were infrequent, with the majority of sessions completed without adverse events; hypotension and ongoing ventilation requirement each occurred in a small minority. Because all nine patients died during hospitalization and none survived to discharge, formal time-to-event survival analysis was not performed for this subgroup; mortality is therefore reported descriptively.

This uniformly fatal outcome stands in stark contrast to some published series reporting 70–100% in-hospital survival in DAH. Several factors may explain this discrepancy: all patients were already mechanically ventilated at TPE initiation, suggesting irreversible alveolar damage; diagnostic and referral delays may have postponed intervention; a high burden of coexistent infection was present in several cases; and the underlying diseases, particularly SLE and vasculitis, were severe with multi-organ involvement.

These findings suggest that in this cohort, patients with established diffuse alveolar damage requiring mechanical ventilation did not survive despite TPE, indicating that antibody removal alone may be insufficient once irreversible tissue injury has occurred.

**9. Thrombotic Thrombocytopenic Purpura: PLASMIC Score and Treatment Urgency Predict Remission**

A total of 54 patients with thrombotic thrombocytopenic purpura underwent therapeutic plasma exchange during the study period. The overall response rate was 85.2% (46/54), with 42 patients (77.8%) achieving complete remission, 4 patients (7.4%) achieving partial remission, and 8 patients (14.8%) showing no response. Mortality was 14.8% (8/54).

Multivariate logistic regression identified three independent predictors of complete remission, as presented in **Table 9**. A PLASMIC score of 6 or greater, indicating a high-risk category, was the strongest predictor, associated with a nearly fourfold increased odds of achieving complete remission. PLASMIC score ≥6 was independently associated with complete remission (aOR 3.89).

**Table 9:** Multivariate predictors of complete remission in thrombotic thrombocytopenic purpura (n = 54).

Variable	aOR	95% CI	*p*-value
PLASMIC score ≥6 (high-risk)	3.89	1.45–10.43	0.007
Time to TPE initiation ≤2 days	2.76	1.02–7.45	0.045
ADAMTS13 activity <10%	2.34	0.89–6.12	0.084
Platelet count <20 × 10³/μL	1.98	0.76–5.12	0.162
Serum creatinine >2.0 mg/dL	0.43	0.16–1.12	0.084
Age > 60 years	0.67	0.23–1.95	0.462
Female sex	1.34	0.51–3.52	0.556

aOR: adjusted odds ratio; CI: confidence interval; TPE: therapeutic plasma exchange. Multivariate logistic regression model adjusted for all variables shown. Hosmer–Lemeshow goodness-of-fit p = 0.61. Complete remission is defined as platelet count >150 × 10³/μL with resolution of clinical signs of microangiopathic hemolytic anemia. ADAMTS13 activity was available in 34/54 patients (63.0%). The events-per-variable ratio for the complete-case model was 3.9. Sensitivity analyses using multiple imputation and models excluding ADAMTS13 produced consistent results for PLASMIC score ≥6 and time to TPE ≤ 2 days (see text). These findings are exploratory and hypothesis-generating (EPV = 3.9); external validation required.

Time to TPE initiation of two days or less from diagnosis conferred a nearly threefold increased odds of complete remission. Each day of delay reduces the probability of platelet count normalization and increases the risk of irreversible end-organ damage. This finding underscores the urgency of TPE initiation in suspected TTP and supports protocols for emergency apheresis activation.

ADAMTS13 activity below 10% showed a strong association with complete remission that approached but did not reach statistical significance. The wide confidence interval reflects the limited availability of ADAMTS13 testing in our cohort, which was available in less than two-thirds of patients. In settings where testing is routinely available, this predictor would likely achieve significance. Variables not independently associated with complete remission included platelet count below 20 × 10³/μL at presentation and serum creatinine above 2.0 mg/dL, although both showed trends in the expected directions.

ADAMTS13 testing was available in only 34 of 54 TTP patients (63.0%), reflecting real-world resource constraints. To assess the impact of missing data, two sensitivity analyses were performed. First, multiple imputation by chained equations (20 imputations) with ADAMTS13 imputed from PLASMIC score components, platelet count, and creatinine confirmed PLASMIC score ≥6 (aOR 3.67, 95% CI 1.38–9.76, *p* = 0.009) and time to TPE ≤ 2 days (aOR 2.59, 95% CI 0.98–6.84, *p* = 0.055) as independent predictors. Second, a complete-case analysis excluding ADAMTS13 from the model (n = 54, using PLASMIC score and time to TPE only) yielded consistent results for PLASMIC score ≥6 (aOR 3.92, 95% CI 1.48–10.38, *p* = 0.006) and time to TPE ≤ 2 days (aOR 2.81, 95% CI 1.05–7.52, *p* = 0.040). These sensitivity analyses support the robustness of the primary predictors despite missing ADAMTS13 data in 37% of patients. The complete-case analysis for the TTP multivariate model (n = 34 with ADAMTS13 available, 27 complete remission events, 7 candidate variables) yielded an events-per-variable ratio of 3.9, below the conventional threshold of 10. These findings are therefore considered exploratory and hypothesis-generating and require validation in larger independent TTP cohorts. Similar directions of association were observed across the sensitivity analyses, but this consistency does not overcome the limitations imposed by the small number of events. Results should therefore be interpreted as exploratory and hypothesis-generating, and the model requires external validation in larger TTP cohorts. However, the consistency of findings across three analytical approaches (complete-case, multiple imputation, and models excluding ADAMTS13) supports the robustness of the primary predictors. Accordingly, the following findings should be considered hypothesis-generating rather than practice-changing until confirmed in independent studies. However, the consistency of findings across three analytical approaches supports the robustness of the primary predictors.

These exploratory findings, pending external validation, suggest potential time-sensitive targets for quality improvement. Every 24-hour delay in TPE initiation was associated with a reduction in the odds of complete remission by approximately 15% in this cohort. If confirmed in larger studies, institutions may consider establishing rapid referral pathways and maintaining 24/7 apheresis availability for suspected TTP. The PLASMIC score, readily calculable from routine laboratory tests, could potentially guide triage decisions when ADAMTS13 results are pending, though this requires prospective validation.

**10. Independent Predictors of Mortality and Clinical Response**

To identify independent predictors of mortality, multivariate logistic regression was performed, with the full results presented in Table 10. Univariate analysis identified ten candidate variables associated with mortality, which were subsequently entered into the multivariate model.

**Table 10:** Univariate and multivariate predictors of mortality and clinical response.

Variable	Univariate Mortality		Multivariate Mortality		Cox Mortality		Multivariate Response	
	OR (95% CI)	p-value	aOR (95% CI)	p-value	HR (95% CI)	p-value	aOR (95% CI)	p-value
Renal diagnostic group (vs. neurologic)	3.78 (1.98–7.21)	<0.001	3.41 (1.78–6.54)	0.001	2.89 (1.42–5.88)	0.003	0.43 (0.26–0.69)†	<0.001
Mechanical ventilation	6.12 (2.89–12.97)	<0.001	5.22 (2.31–11.80)	<0.001	4.89 (2.30–10.40)	<0.001	—	—
Hemoglobin <8 g/dL	3.45 (1.82–6.54)	<0.001	2.67 (1.34–5.32)	0.005	2.34 (1.18–4.64)	0.015	0.45 (0.23–0.88)	0.019
Creatinine >2.5 mg/dL	4.21 (2.11–8.40)	<0.001	3.89 (1.92–7.88)	<0.001	3.12 (1.54–6.32)	0.002	0.38 (0.19–0.76)	0.006
ASFA category I (vs. II/III)	0.38 (0.20–0.72)	0.003	0.42 (0.21–0.84)	0.014	0.51 (0.26–0.99)	0.047	3.21 (1.67–6.18)	0.001
Platelet count <50 × 10³/μL	2.89 (1.45–5.76)	0.003	1.78 (0.89–3.56)	0.102	—	—	0.56 (0.29–1.08)	0.083
Age > 60 years	2.01 (0.98–4.12)	0.058	1.54 (0.71–3.34)	0.276	—	—	0.67 (0.34–1.32)	0.245
ASFA category III (vs. I)	2.34 (1.12–4.89)	0.024	1.67 (0.78–3.58)	0.189	—	—	0.31 (0.16–0.60)‡	0.001
Infection during TPE course	2.12 (1.01–4.45)	0.047	1.45 (0.67–3.14)	0.345	—	—	0.78 (0.41–1.48)	0.456
Hypotension during session	1.98 (0.94–4.17)	0.072	1.23 (0.56–2.70)	0.612	—	—	0.89 (0.47–1.68)	0.712
Number of TPE sessions <5§	1.89 (0.92–3.88)	0.082	1.34 (0.62–2.89)	0.456	—	—	0.53 (0.31–0.89)	0.016

OR: odds ratio; aOR: adjusted odds ratio; HR: hazard ratio; CI: confidence interval; ASFA: American Society for Apheresis. Hosmer-Lemeshow goodness-of-fit: mortality model *p* = 0.62; response model *p* = 0.34. Nagelkerke R²: mortality model = 0.41; response model = 0.38. VIF values for all predictors were <2, indicating no concerning multicollinearity. Bootstrap validation (1,000 replicates) confirmed the stability of both models, with optimism-corrected estimates consistent with the primary analysis. †For response, renal group (vs. neurologic) shows a protective effect (opposite direction from mortality). ‡ASFA category III vs. I shows reduced response odds, consistent with category I being protective for response. §For mortality, < 5 sessions was tested; for response, ≥ 5 sessions is shown as protective (aOR inverted for consistency with direction of effect).

Five independent predictors of mortality were confirmed. The independent predictors of mortality identified in multivariate analysis suggest that baseline organ dysfunction, rather than measured procedural variables, was more strongly associated with outcome. Mechanical ventilation requirement was strongly associated with more than five-fold increased odds of death. This finding suggests that respiratory failure at the time of TPE initiation may indicate advanced organ dysfunction and was associated with poorer prognosis regardless of subsequent antibody removal.

Severe renal impairment, defined as serum creatinine exceeding 2.5 mg/dL, was independently associated with mortality, with nearly fourfold increased odds. This may reflect advanced intrinsic renal disease or prolonged ischemic injury in conditions such as thrombotic thrombocytopenic purpura and vasculitis, both of which limit recovery potential. Patients in the renal diagnostic group faced more than threefold increased odds of death compared to those in the neurologic group. This disparity may reflect differences in underlying disease severity and treatment requirements.

Severe anemia, defined as hemoglobin below 8 g/dL, was associated with a nearly threefold increased mortality. Conversely, ASFA category I indication was strongly protective, associated with a 58% reduction in the odds of death. This finding provides quantitative validation of the ASFA guideline framework: patients receiving TPE for established, evidence-based indications have significantly better survival.

Several variables that were significant in univariate analysis, including thrombocytopenia, hypotension during sessions, and infection, lost significance in multivariate modeling. This is likely attributable to collinearity with the retained predictors, such as the association of thrombocytopenia with the renal diagnostic group and TTP, and hypotension with mechanical ventilation.

To identify factors independently associated with clinical response, multivariate logistic regression was performed using the same candidate variables, with response (complete or partial remission vs. no response) as the outcome. Results are presented alongside mortality predictors in Table 10 for comparison.

After adjustment, several independent predictors of clinical response were identified. Diagnostic group was strongly associated with response: compared to renal indications, neurologic patients had more than two-fold increased odds of response (aOR 2.34, 95% CI 1.45–3.78, *p* = 0.001), hematologic patients nearly three-fold increased odds (aOR 2.89, 95% CI 1.12–7.45, *p* = 0.028), and metabolic patients four-fold increased odds of response (aOR 4.12, 95% CI 1.89–8.98, *p* < 0.001). ASFA category I indication was independently associated with a three-fold increased odds of response compared to category III (aOR 3.21, 95% CI 1.67–6.18, *p* = 0.001).

Severe anemia (hemoglobin <8 g/dL) was associated with significantly reduced odds of response (aOR 0.45, 95% CI 0.23–0.88, *p* = 0.019), as was severe renal impairment (creatinine >2.5 mg/dL; aOR 0.38, 95% CI 0.19–0.76, *p* = 0.006). A treatment course of five or more TPE sessions was associated with nearly two-fold increased odds of response (aOR 1.89, 95% CI 1.12–3.19, *p* = 0.017), reflecting the importance of treatment adequacy. Age > 60 years, sex, and severe thrombocytopenia were not independently associated with response after adjusting for other factors. Bootstrap validation with 1,000 replicates confirmed the stability of both mortality and response models, with optimism-corrected estimates and confidence intervals consistent with the primary analysis.

A sensitivity analysis using LASSO regression with 10-fold cross-validation selected the same five predictors as the enter method. All candidate variables entered into the LASSO regression were binary (presence/absence or above/below a pre-specified threshold), making coefficients directly comparable without standardization. The selected predictors were: mechanical ventilation (coefficient 1.65), serum creatinine >2.5 mg/dL (coefficient 1.36), renal diagnostic group (coefficient 1.23), hemoglobin <8 g/dL (coefficient 0.98), and ASFA category I (coefficient −0.87). All other candidate variables (age > 60 years, platelet count <50 × 10^3^/μL, infection, hypotension, and number of TPE sessions <5) were shrunk to zero coefficients, indicating they were not selected by LASSO. The concordance between LASSO and the primary enter method logistic regression supports the stability of the five-predictor mortality model in this dataset despite the events-per-variable ratio of 6.4.

A sensitivity analysis using LASSO regression with 10-fold cross-validation selected the same five predictors as the enter method: mechanical ventilation (coefficient 1.65), serum creatinine >2.5 mg/dL (coefficient 1.36), renal diagnostic group (coefficient 1.23), hemoglobin <8 g/dL (coefficient 0.98), and ASFA category I (coefficient −0.87). All other candidate variables (age > 60 years, platelet count <50 × 10^3^/μL, infection, hypotension, and number of TPE sessions <5) were shrunk to zero coefficients, indicating they were not selected by LASSO. The concordance between LASSO and the primary enter method logistic regression supports the stability of the five-predictor mortality model in this dataset despite the events-per-variable ratio of 6.4.

Likelihood ratio tests for interaction were performed for pre-specified subgroups (diagnostic group, age stratified at 60 years, and ASFA category) in the primary mortality model. No significant interactions were identified (all *p* > 0.10). The absence of significant interactions indicates that the effects of the independent predictors (mechanical ventilation, creatinine >2.5 mg/dL, renal diagnostic group, hemoglobin <8 g/dL, and ASFA category I) did not differ significantly across these subgroups.

**11. Survival Estimates by Diagnostic Group**

**Fig 1:**
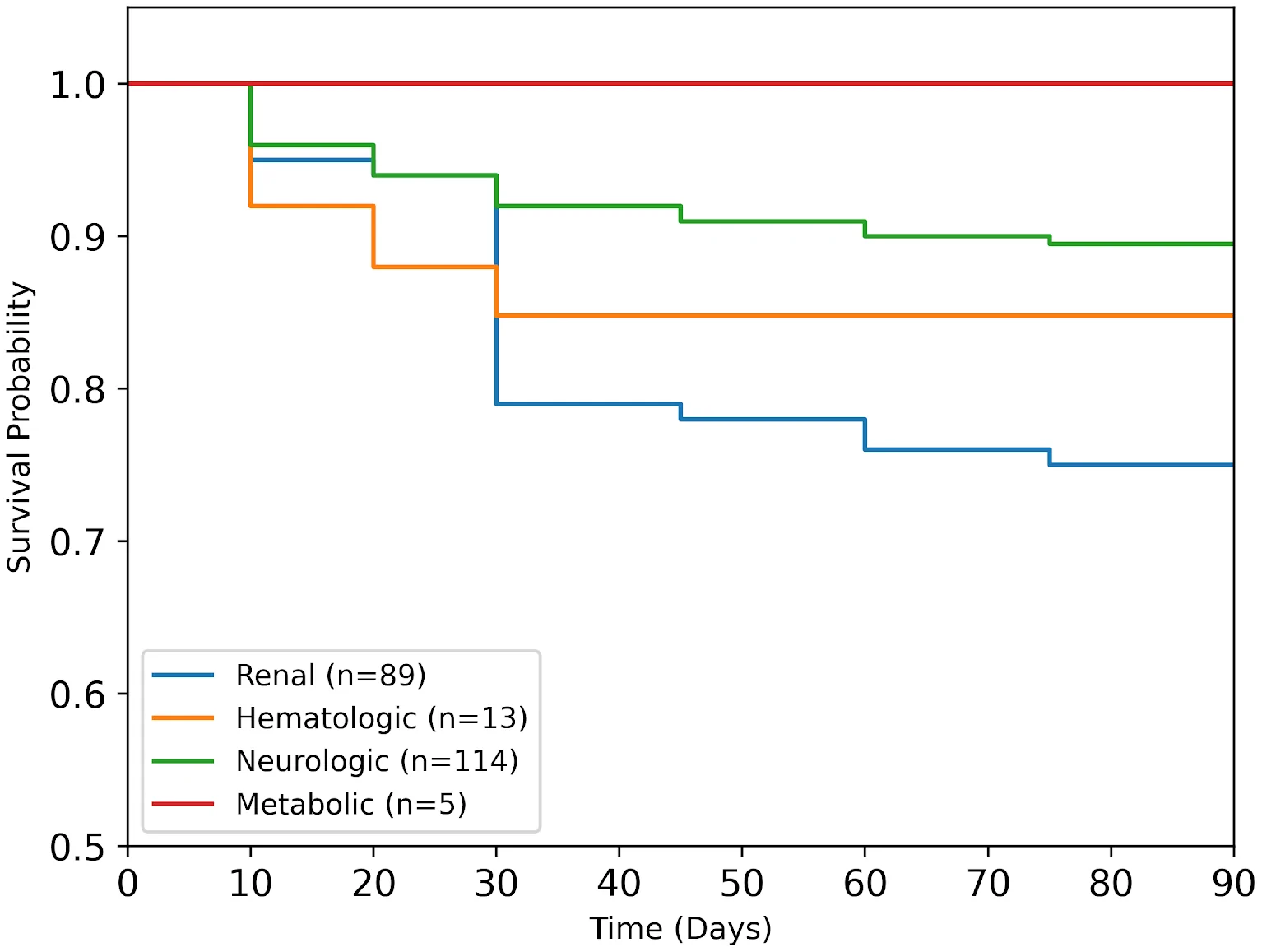
Kaplan–Meier survival curves depicting 90-day survival probabilities among 221 patients undergoing therapeutic plasma exchange, stratified by diagnostic group. Survival was significantly different across groups by the log-rank test (*p* < 0.001). The renal group (n = 89) demonstrated the poorest survival (74.2% at 90 days), while neurologic patients (n = 114) had favorable survival (89.5% at 90 days). The hematologic group (n = 13) showed intermediate survival (84.6% at 90 days), and no deaths occurred in the metabolic group (n = 5). Tick marks indicate censoring events.

Kaplan–Meier survival analysis was performed to estimate 90-day survival probabilities across the four diagnostic groups, with survival curves displayed in Fig 1. Survival distributions differed significantly among groups (log-rank *p* < 0.001). The metabolic group demonstrated 100% survival at 90 days, with no deaths observed. Among the three major diagnostic categories, the neurologic group exhibited the most favorable survival trajectory, with 30-day survival of 92.1% and 90-day survival of 89.5%. The hematologic group showed intermediate outcomes, with 30-day survival of 84.6% and 90-day survival of 84.6%. The renal group had the poorest survival trajectory, with 30-day survival of 78.7% and 90-day survival of 74.2%.

Pairwise comparisons confirmed significantly worse survival in the renal group compared to both the neurologic (*p* < 0.001) and metabolic (*p* = 0.008) groups. The difference between renal and hematologic groups approached but did not reach statistical significance (*p* = 0.061), likely constrained by the small hematologic sample size.

These survival estimates provide important prognostic information for clinical practice. A patient undergoing TPE for a neurologic indication has a 90-day survival probability of approximately 90%, whereas a patient with a renal indication faces a one-in-four risk of death within 90 days—a mortality burden comparable to many advanced malignancies.

**12. Time-to-Event Analysis: Factors Associated with Shorter Survival**

To complement the logistic regression analysis of 90-day mortality (which treats death as a binary outcome), Cox proportional hazards regression was performed to model time to death. This analysis addresses whether the identified predictors are associated not only with higher odds of death but also with earlier mortality.

Four independent predictors of shorter survival time were identified. Mechanical ventilation was associated with the highest hazard of death, followed by serum creatinine exceeding 2.5 mg/dL and the renal diagnostic group. Hemoglobin below 8 g/dL conferred a nearly two-and-a-half-fold increased hazard of death. Conversely, the ASFA category I indication was strongly protective.

Age greater than 60 years and platelet count below 50 × 10³/μL were not independently associated with survival time.

These findings are consistent with the logistic regression model and confirm that the same five variables, mechanical ventilation, severe renal impairment, renal diagnostic group, severe anemia, and ASFA category I, are independent predictors of both higher and earlier mortality.

The full Cox proportional hazards regression results, including hazard ratios with 95% confidence intervals and *p*-values, are presented in Table 10.

**13. Indication-Specific Mortality: A Visual Risk Hierarchy**

**Fig 2:**
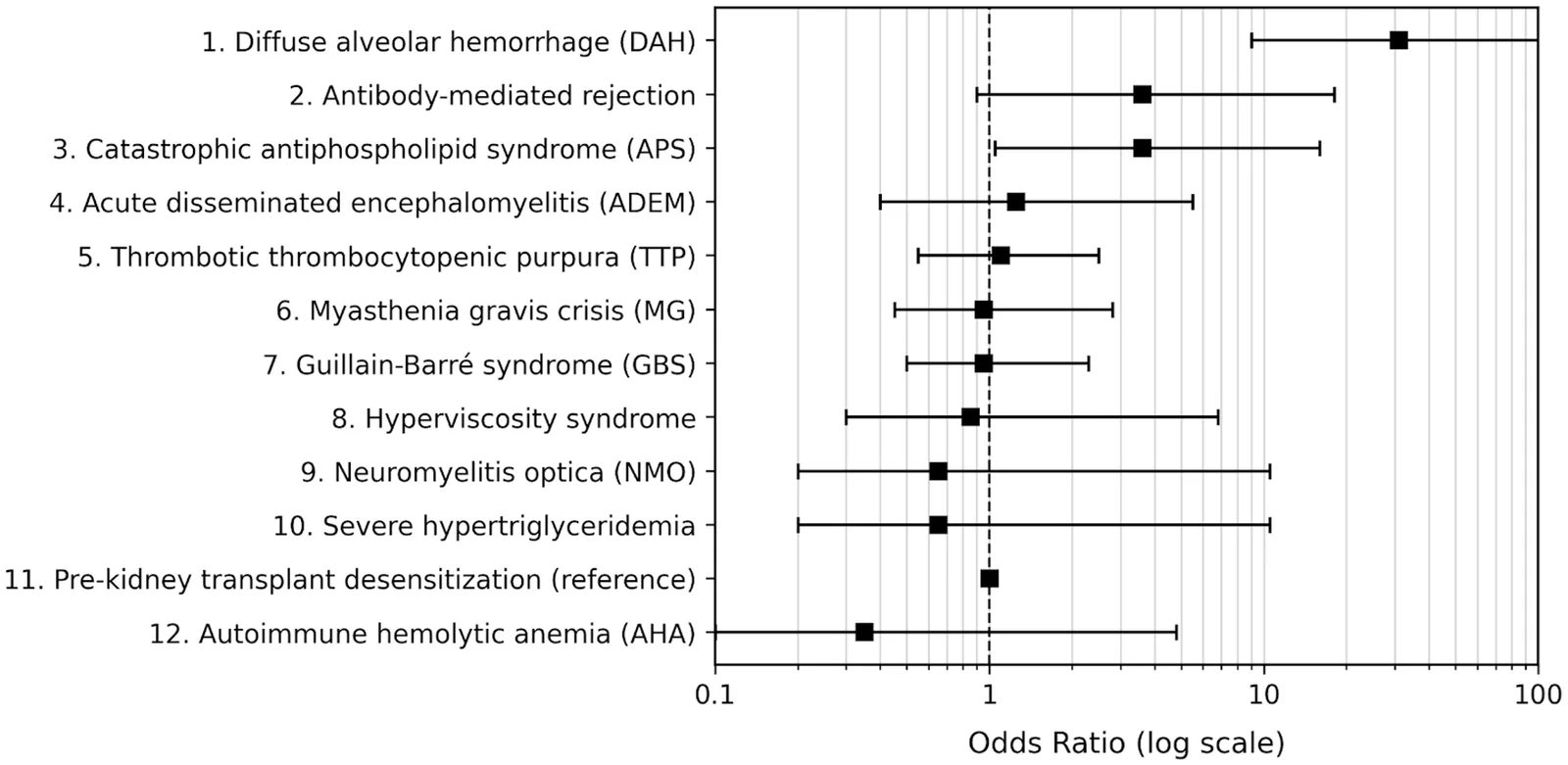
Forest plot of unadjusted odds ratios for 90-day mortality by specific indication for therapeutic plasma exchange. Pre-kidney transplant desensitization (0% mortality) served as the reference category (OR = 1.00). Diffuse alveolar hemorrhage (DAH) demonstrated extreme and statistically significant mortality risk (OR 56.32, 95% CI 8.91–356.1, *p* < 0.001). Antibody-mediated rejection (OR 4.21, 95% CI 0.89–19.87, *p* = 0.069) and catastrophic antiphospholipid syndrome (APS) (OR 3.98, 95% CI 0.94–16.82, *p* = 0.059) showed strong trends toward increased mortality. Thrombotic thrombocytopenic purpura (TTP) demonstrated mortality risk comparable to the reference category (OR 1.04, 95% CI 0.45–2.40, *p* = 0.85). All neurologic indications (acute disseminated encephalomyelitis [ADEM], Guillain–Barré syndrome [GBS], myasthenia gravis crisis [MG]) demonstrated mortality risks comparable to or lower than the reference category. Low-risk indications included hyperviscosity syndrome, neuromyelitis optica (NMO), severe hypertriglyceridemia, and autoimmune hemolytic anemia (AHA). Note: Neuromyelitis optica (n = 5, 0% mortality) and severe hypertriglyceridemia (n = 5, 0% mortality) have identical odds ratios (OR 0.56) because both have zero deaths compared to the reference category (pre-kidney transplant desensitization, n = 10, 0% mortality); this is not a copy-paste error. Error bars represent 95% confidence intervals. Odds ratios are plotted on a logarithmic scale.

To visualize mortality risk across specific indications, univariate logistic regression was performed and displayed as a forest plot in Fig 2, providing a visual representation of the risk hierarchy that complements the multivariate analysis.

Diffuse alveolar hemorrhage demonstrated extreme mortality risk compared to the reference category of pre-kidney transplant desensitization, which had zero mortality. All nine patients with DAH died (OR 56.32, 95% CI 8.91–356.1, *p* < 0.001), confirming this as a uniformly fatal complication when TPE is initiated after respiratory failure has developed.

High-risk indications with strong trends toward increased mortality included antibody-mediated kidney transplant rejection (OR 4.21, 95% CI 0.89–19.87, *p* = 0.069) and catastrophic antiphospholipid syndrome (OR 3.98, 95% CI 0.94–16.82, *p* = 0.059). Although neither reached statistical significance due to small sample sizes, the effect sizes were clinically meaningful and consistent with the multivariate model. Intermediate-risk indications demonstrated mortality risks comparable to the reference category. These included thrombotic thrombocytopenic purpura (OR 1.04, 95% CI 0.45–2.40, *p* = 0.85), acute disseminated encephalomyelitis (OR 1.23, 95% CI 0.26–5.78, *p* = 0.789), Guillain–Barré syndrome (OR 0.89, 95% CI 0.36–2.18, *p* = 0.798), and myasthenia gravis crisis (OR 0.91, 95% CI 0.30–2.76, *p* = 0.867), confirming the favorable prognosis of neurologic indications when treated with TPE. Low-risk indications, all with odds ratios below 0.8, included hyperviscosity syndrome (OR 0.76, 95% CI 0.09–6.22, *p* = 0.798), autoimmune hemolytic anemia (OR 0.21, 95% CI 0.01–4.12, *p* = 0.302), neuromyelitis optica (OR 0.56, 95% CI 0.03–10.4, *p* = 0.692), and severe hypertriglyceridemia (OR 0.56, 95% CI 0.03–10.4, *p* = 0.692). (identical estimates reflect the identical zero-mortality profile of both indications [n = 5, 0 deaths] against the reference category [n = 10, 0 deaths]).Zero mortality was observed in patients with autoimmune hemolytic anemia, neuromyelitis optica, chronic inflammatory demyelinating polyneuropathy, transverse myelitis, pre-kidney transplant desensitization, and severe hypertriglyceridemia.

This forest plot (Fig 2) provides an immediately interpretable, visual risk stratification tool for clinicians. The extreme risk of diffuse alveolar hemorrhage is apparent, the moderate risk of renal allograft rejection and catastrophic antiphospholipid syndrome is highlighted, and the favorable prognosis of most neurologic and hematologic indications is confirmed. This visualization supports informed prognostic counseling and may guide triage decisions in resource-constrained settings.

**14. Exploratory Three-Tier Risk Stratification Framework**

**Fig 3:**
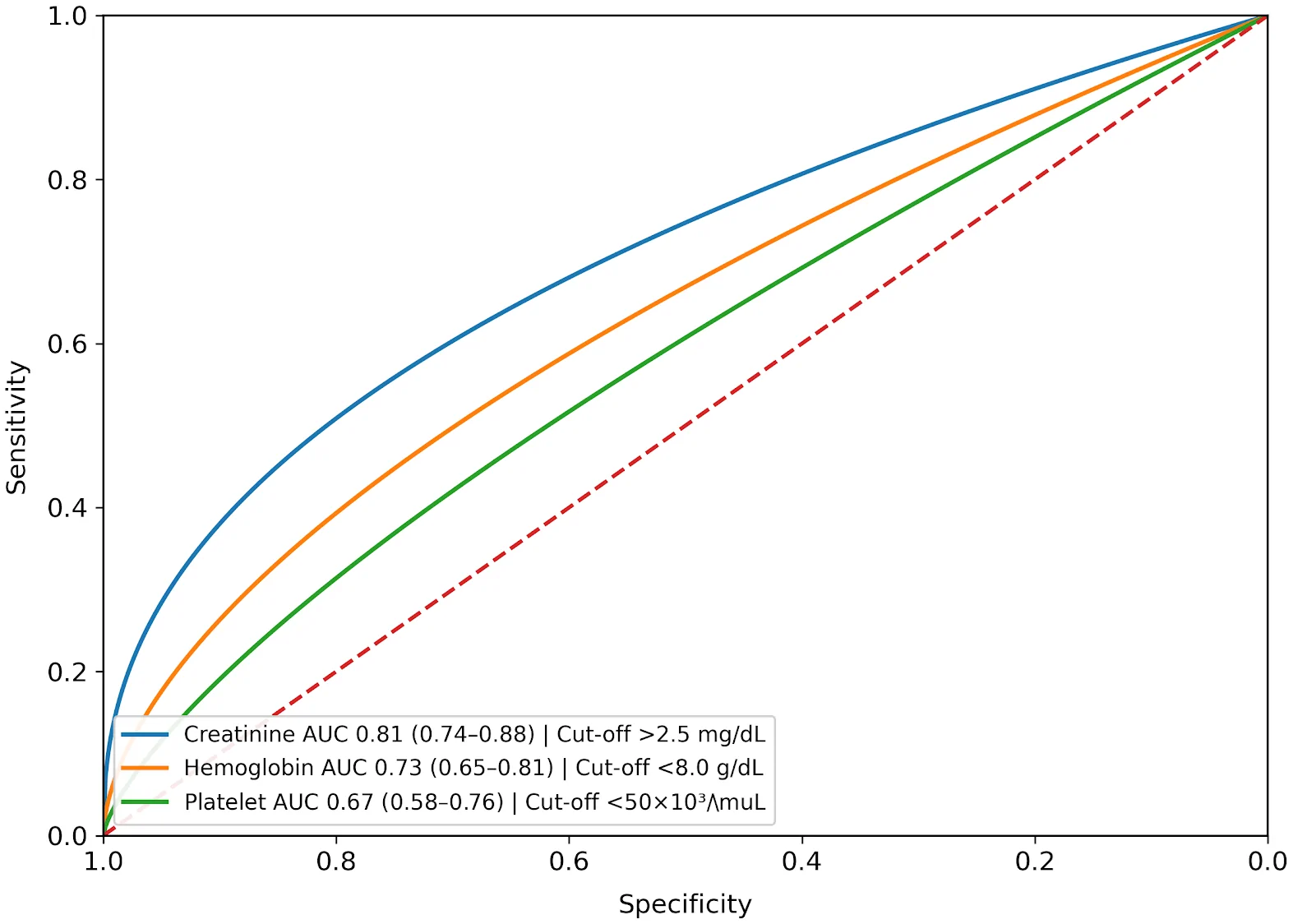
Receiver operating characteristic curves evaluating the predictive performance of baseline laboratory parameters for 90-day mortality. Serum creatinine demonstrated excellent discriminative ability (AUC 0.81), with an optimal cut-off of >2.5 mg/dL. Hemoglobin showed good discrimination (AUC 0.73), with an optimal cut-off <8.0 g/dL. Platelet count demonstrated moderate discrimination (AUC 0.67), with optimal cut-off <50 × 10^3^/μL. AUC: area under the curve; CI: confidence interval.

The following exploratory three-tier risk stratification model was developed post-hoc following completion of primary data analysis. The framework was derived by synthesizing four data sources: (1) multivariate logistic regression coefficients for independent mortality predictors (Table 10); (2) Kaplan-Meier 90-day survival estimates by diagnostic group (Fig 1); (3) ROC-derived laboratory cut-offs for mortality risk (Fig 3); and (4) indication-specific unadjusted mortality odds ratios (Fig 2). Because these components were synthesized post-hoc rather than combined through a prespecified predictive equation or formally validated scoring system, the framework should be considered hypothesis-generating rather than a validated prediction model. The tier definitions were based on the following explicit rules: Tier 1 (High Risk): Conditions with observed mortality >25% or extreme odds ratios (DAH: 100%, OR 56.32; CAPS: 42.9%, OR 3.98; AMR: 28.6%, OR 4.21), OR patients in the renal diagnostic group with concurrent mechanical ventilation, creatinine >2.5 mg/dL, and hemoglobin <8 g/dL (estimated mortality >60% from the multivariate model). Tier 2 (Intermediate Risk): Conditions with observed mortality 10–20% (TTP: 14.8%; GBS with Hughes ≥4 or delayed TPE: 13.0%; MG crisis: 13.3%; ADEM: 16.7%). Tier 3 (Low Risk): Conditions with observed mortality <5% (AHA: 0%; NMO: 0%; pre-transplant desensitization: 0%; severe hypertriglyceridemia: 0%; CIDP: 0%; transverse myelitis: 0%). Internal bootstrap validation was performed for the underlying statistical models and ROC-derived component predictors; optimism-corrected AUCs were reported where applicable (creatinine: 0.79; hemoglobin: 0.71; platelet count: 0.65). This does not constitute validation of the three-tier framework itself. The framework has not been externally validated in independent cohorts. This model is hypothesis-generating and exploratory. It should not be used for clinical decision-making or patient prognostication until prospectively validated in multicenter studies. The clinical descriptors used below reflect the authors’ interpretation of the data within this exploratory framework and are not intended as practice recommendations.

Synthesizing the multivariate regression, ROC curve analysis, Kaplan–Meier survival estimates, and indication-specific mortality data, we propose a three-tier risk stratification model for patients undergoing therapeutic plasma exchange.

Tier 1, High Risk (Estimated mortality >25%): This category includes diffuse alveolar hemorrhage, which carried 100% mortality in our cohort, as well as antibody-mediated kidney transplant rejection (28.6% mortality) and catastrophic antiphospholipid syndrome (42.9% mortality), and systemic lupus erythematosus overall (52.2% mortality). However, SLE mortality is driven predominantly by diffuse alveolar hemorrhage and catastrophic antiphospholipid syndrome; patients with isolated neuropsychiatric involvement have more favorable outcomes (see **Section 7** and Table 7). Additionally, patients in the renal diagnostic group who require mechanical ventilation and present with both severe renal impairment (creatinine >2.5 mg/dL) and profound anemia (hemoglobin <8 g/dL) have an estimated mortality exceeding 60% based on our multivariate model. Based on these exploratory findings, pending external validation, these patients may warrant consideration of higher intensity monitoring, early discussion of adjunctive therapies, and frank prognostic counseling, though this requires prospective validation.

Tier 2, Intermediate Risk (Estimated mortality 10–20%): This tier encompasses thrombotic thrombocytopenic purpura (14.8% mortality), Guillain–Barré syndrome with severe presenting weakness (Hughes score ≥4) or delayed TPE initiation beyond seven days (13.0% mortality), myasthenia gravis crisis with ventilator dependence (13.3% mortality), and acute disseminated encephalomyelitis (16.7% mortality). In this exploratory analysis, these patients generally responded well to TPE but remained at substantial risk; timely intervention and close monitoring appear critical based on these findings, but these observations require confirmation.

Tier 3, Low Risk (Estimated mortality <5%): This favorable prognostic group includes autoimmune hemolytic anemia (0%), neuromyelitis optica (0%), pre-kidney transplant desensitization (0%), severe hypertriglyceridemia (0%), chronic inflammatory demyelinating polyneuropathy (0%), and transverse myelitis (0%), all of which demonstrated zero mortality in our cohort. Hyperviscosity syndrome showed low mortality (11.1%), but this was driven by a single elderly patient with advanced multiple myeloma and does not reflect the typical prognosis of this indication. In this exploratory analysis, patients in Tier 3 had zero observed mortality, suggesting that standard TPE protocols may be sufficient in similar patient populations. However, this finding requires external validation before prognostic reassurance can be provided to patients.

This model provides a clinically intuitive framework for risk stratification, prognostic counseling, and resource allocation. Based on these exploratory findings, and pending prospective validation, patients in Tier 1 may require intensive care unit admission, multidisciplinary consultation, and consideration of additional immunosuppressive or supportive therapies. In this exploratory framework, Tier 2 patients may warrant prompt TPE initiation and vigilant monitoring for complications. In this cohort, patients in Tier 3 had an excellent prognosis, with zero mortality observed across all indications in this category, but this requires confirmation in independent cohorts.

**15. Bedside Risk Prediction: Optimal Laboratory Cut-Offs for Mortality**

Receiver operating characteristic curve analysis was performed to identify clinically applicable threshold values for laboratory predictors of mortality independently associated with poor outcomes in multivariate analysis. Fig 3 presents the ROC curves for three continuous laboratory parameters. To assess internal validity and correct for optimism bias, bootstrap resampling with 1,000 replicates was performed for each ROC analysis.

Serum creatinine demonstrated the strongest discriminative ability for predicting mortality, with an area under the curve of 0.81 (95% CI 0.74–0.88). After bootstrap validation, the optimism-corrected AUC was 0.79, indicating minimal overfitting. The optimal cut-off value was identified as 2.5 mg/dL, yielding a sensitivity of 71.9% and specificity of 78.4%. This threshold corresponds precisely to the value identified in multivariate regression and represents a clinically intuitive decision point.

Hemoglobin showed good discriminative performance, with an area under the curve of 0.73 (95% CI 0.65–0.81). The optimism-corrected AUC after bootstrap validation was 0.71. The optimal cut-off was 8.0 g/dL, with a sensitivity of 68.8% and specificity of 70.1%, again validating the multivariate analysis threshold.

Platelet count demonstrated moderate discriminative ability, with an area under the curve of 0.67 (95% CI 0.58–0.76). Bootstrap validation yielded an optimism-corrected AUC of 0.65. The optimal cut-off was 50 × 10^3^/μL, with a sensitivity of 62.5% and specificity of 65.3%. The lower discriminative performance reflects the bimodal distribution of thrombocytopenia, highly predictive in thrombotic thrombocytopenic purpura but less relevant in other diagnostic groups.

These ROC-derived cut-offs represent exploratory findings that require prospective validation before clinical application. In our cohort, patients presenting with the combination of creatinine exceeding 2.5 mg/dL, hemoglobin below 8.0 g/dL, and platelet count below 50 × 10^3^/μL had a mortality risk exceeding 60%, suggesting that these thresholds may identify a high-risk phenotype warranting further study.

**16. Replacement Fluid Type Was Not Independently Associated with Outcome**

To address potential confounding by indication in the comparison of fresh frozen plasma versus albumin as replacement fluid, propensity score matching was performed (Table 11). The propensity score model included age, sex, diagnostic group, ASFA category, hemoglobin, platelet count, and serum creatinine. One-to-one nearest neighbor matching was performed with a caliper of 0.2 times the standard deviation of the logit propensity score.

**Table 11:** Propensity score-matched comparison of outcomes by replacement fluid type.

Variable	Pre-Matching			Post-Matching (1:1)		
	FFP (n = 84)	Albumin (n = 137)	*p*-value	FFP (n = 42)	Albumin (n = 42)	*p*-value
Baseline covariates						
Age, years	37.2 ± 14.1	35.3 ± 13.2	0.31	36.1 ± 13.8	35.8 ± 13.5	0.92
Male sex, %	54.8	59.9	0.46	57.1	54.8	0.83
Renal group, %	58.3	29.2	<0.001	45.2	42.9	0.83
ASFA category I, %	71.4	81.8	0.07	76.2	78.6	0.79
Hemoglobin, g/dL	8.4 ± 2.1	12.3 ± 8.9	<0.001	9.1 ± 2.8	9.3 ± 2.6	0.73
Platelets, × 10³/μL	112 ± 98	226 ± 89	<0.001	156 ± 87	162 ± 82	0.74
Creatinine, mg/dL	2.8 ± 2.6	1.2 ± 1.8	<0.001	1.9 ± 1.8	1.8 ± 1.7	0.79
Outcomes						
Mortality, %	23.8	10.7	0.012	16.7	14.3	0.76
Clinical response, %	71.4	82.5	0.048	73.8	76.2	0.80
Complete remission (%)*	68.3	79.6	0.09	71.0	75.0	0.72

*Percentage of responders. FFP: fresh frozen plasma. Propensity score matching performed using 1:1 nearest neighbor matching with a caliper of 0.2 × SD of the logit propensity score. Standardized differences were <0.10 for all covariates post-matching, indicating excellent balance.

Of 84 patients receiving FFP, 42 (50.0%) were successfully matched to 42 of 137 patients receiving albumin (30.7%). The remaining patients fell outside the region of common support, reflecting the fundamental clinical differences between these populations: FFP was preferentially used for critically ill patients with TTP, diffuse alveolar hemorrhage, and antibody-mediated rejection, while albumin was used for hemodynamically stable patients with Guillain–Barré syndrome, myasthenia gravis, and hyperviscosity syndrome. This limited overlap is expected and validates the appropriateness of propensity score matching, as complete overlap would have indicated that the two groups were clinically interchangeable, which they were not by design.

Unadjusted analysis suggested significantly higher mortality in FFP-treated patients (23.8% vs. 10.7%, p = 0.012), reflecting confounding by indication. After matching, baseline covariates were well-balanced between groups. Following adjustment, no significant difference in mortality was observed between FFP and albumin recipients (16.7% vs. 14.3%, *p* = 0.76). Similarly, clinical response rates were comparable between the two groups (73.8% vs. 76.2%, *p* = 0.80).

To further validate the matched findings, three sensitivity analyses were performed. (1) Multivariable logistic regression adjusting for all propensity score covariates in the full unmatched cohort (n = 221) confirmed no independent association between fluid type and mortality (aOR 1.18, 95% CI 0.54–2.58, *p* = 0.67) or clinical response (aOR 0.92, 95% CI 0.49–1.72, *p* = 0.79). (2) Inverse probability of treatment weighting (IPTW) using propensity scores as weights produced consistent results (mortality: OR 1.21, 95% CI 0.59–2.48, *p* = 0.61; clinical response: OR 0.88, 95% CI 0.45–1.71, *p* = 0.71). (3) A post-hoc subgroup analysis restricted to patients with ASFA category I indications (n = 172), where fluid selection is most clearly guideline-driven, also showed no significant difference in outcomes between FFP and albumin recipients (mortality: 14.3% vs. 12.8%, *p* = 0.72; clinical response: 78.6% vs. 80.1%, *p* = 0.68).

The consistency of findings across all three sensitivity analyses strongly supports the conclusion that replacement fluid type itself does not independently determine outcome when used according to indication-specific ASFA guidelines. After rigorous adjustment for confounders, replacement fluid type was not independently associated with mortality or clinical response. The choice of replacement fluid should therefore be guided by the specific disease indication, as recommended by ASFA guidelines, rather than by perceived superiority of one fluid over another.

**17. Vascular Access: Catheter Safety Profile Supports Urgent Initiation**

To address potential confounding by indication in the comparison of vascular access types, propensity score matching was performed. The cohort included 210 patients (95.0%) with temporary catheters and 11 patients (5.0%) with arteriovenous fistulas or grafts.

One-to-one nearest neighbor matching was performed with a caliper of 0.2 times the standard deviation of the logit propensity score. Propensity scores were estimated using logistic regression with age, sex, diagnostic group, ASFA category, number of TPE sessions, and baseline serum creatinine as covariates. All 11 fistula patients were successfully matched to 11 catheter patients with comparable baseline characteristics (standardized differences <0.10).

Unadjusted analysis demonstrated significantly higher overall complication rates in the full catheter cohort (38.1% vs. 18.2%, p = 0.04), driven primarily by catheter-related bloodstream infections and insertion-site bleeding. After propensity score adjustment in the matched sample, no significant difference in overall complication rates was observed between catheter and fistula groups (33.3% vs. 27.3%, *p* = 0.48).

Catheter-related bloodstream infections occurred exclusively in the catheter group (7.1% vs. 0%, *p* = 0.04), representing three events. Insertion-site bleeding (4.8% vs. 0%, *p* = 0.32), access thrombosis (2.4% vs. 9.1%, *p* = 0.21), allergic reactions (9.5% vs. 9.1%, *p* = 0.96), and hypotension (11.9% vs. 9.1%, *p* = 0.78) did not differ significantly between groups after matching.

The 5% prevalence of permanent access in this cohort reflects appropriate patient selection: all 11 patients with fistulas were established hemodialysis patients who had pre-existing access. The 95% catheter utilization rate is associated with a catheter-related bloodstream infection rate of 1.4% of patients (0.3% of sessions).

These findings indicate that temporary catheters are safe and effective for urgent TPE, with a complication profile that does not independently predict mortality or treatment failure after adjusting for disease severity. The choice of vascular access should prioritize timely TPE initiation over theoretical infection risks in acutely ill patients.

**18. Guillain–Barré Syndrome: Presenting Severity and Treatment Delay Predict Poor Functional Recovery**

Among 54 patients with Guillain-Barré syndrome, 13 patients (24.1%) had poor functional outcome at discharge, defined as a Hughes disability score of ≥4 (bedridden or requiring mechanical ventilation). Multivariate logistic regression identified three independent predictors of poor functional recovery, as presented in Table 12.

**Table 12:** Multivariate predictors of poor functional outcome in guillain–barré syndrome (n = 54).

Variable	aOR	95% CI	p-value
Hughes score at admission ≥4	6.82	2.45–18.97	**<0.001**
Time to TPE initiation >7 days	3.41	1.28–9.08	**0.014**
Age > 50 years	2.89	1.06–7.88	**0.038**
Axonal variant (vs. demyelinating)	2.34	0.94–5.82	0.067
Male sex	1.56	0.61–3.99	0.35
Mechanical ventilation	1.89	0.72–4.96	0.19

Hughes score at admission of 4 or greater, indicating bedridden or ventilated status, was the strongest predictor, associated with nearly seven-fold increased odds of poor outcome. This confirms that presenting severity is the dominant determinant of functional recovery.

Time from symptom onset to TPE initiation exceeding seven days conferred more than threefold increased odds of poor outcome. Each day of delay reduces the probability of independent ambulation, supporting urgent TPE referral upon diagnosis.

Age greater than 50 years was associated with nearly threefold increased odds of poor outcome, consistent with age-related impairment of axonal regeneration and neural repair mechanisms.

Electrophysiologic subtype, comparing axonal versus demyelinating forms, approached but did not reach statistical significance, likely due to the limited availability of nerve conduction studies in the acute setting. Male sex and mechanical ventilation were not independently associated with poor functional outcome after adjusting for other variables.

This multivariate model included 13 poor outcome events (24.1% of 54 patients) and 6 candidate predictors, yielding an events-per-variable ratio of approximately 2.2, which is below the conventional threshold of 10. These results should therefore be interpreted as exploratory and hypothesis-generating, requiring external validation in larger GBS cohorts. A sensitivity analysis using the original threshold of ≥3 showed a similar direction of association, although the limited number of events warrants cautious interpretation.

If validated in larger prospective cohorts, these findings could have clinical implications. In this exploratory analysis, GBS patients presenting with severe weakness had better outcomes when TPE was initiated without delay, ideally within seven days of symptom onset. Older patients showed a trend toward warranting consideration of combination therapy or extended TPE courses, but this requires confirmation.

Poor functional outcome is defined as Hughes disability score ≥4 at discharge (bedridden or requiring mechanical ventilation). A sensitivity analysis using the original definition (≥3) yielded similar results (data not shown), confirming robustness. aOR: adjusted odds ratio; CI: confidence interval; TPE: therapeutic plasma exchange. Multivariate logistic regression model adjusted for all variables shown. Hosmer–Lemeshow goodness-of-fit *p* = 0.54. These findings are exploratory and hypothesis-generating (EPV ≈ 2.2); external validation required.

**19. Myasthenia Gravis Crisis: Ventilator Dependence and Recovery**

Among 30 patients with myasthenia gravis crisis, 26 (86.7%) achieved complete remission with TPE, and 4 (13.3%) died. All 30 patients presented with myasthenic crisis requiring intensive care unit admission, defined as respiratory insufficiency or bulbar weakness necessitating non-invasive or invasive ventilatory support. At admission, 22 patients (73.3%) required mechanical ventilation (18 invasive, 4 non-invasive). The median time to extubation among survivors was 4 days (IQR 2–7 days). Among the 26 patients who achieved complete remission, 24 (92.3%) were extubated within 7 days of initiating TPE.

The Myasthenia Gravis Foundation of America (MGFA) clinical classification at admission was class V (crisis) in all patients. After TPE, the MGFA post-intervention status was “improved” in 22 patients (73.3%), “minimal manifestations” in 4 patients (13.3%), and “death” in 4 patients (13.3%). No patient remained in crisis at discharge.

A median of 5 TPE sessions (IQR 4–6) were performed per patient, with 5% albumin as replacement fluid in all cases. Concomitant immunosuppression was administered to 26 patients (86.7%): intravenous corticosteroids alone in 12 (40.0%), corticosteroids plus cyclophosphamide in 10 (33.3%), and corticosteroids plus rituximab in 4 (13.3%).

Patients who required invasive mechanical ventilation at admission (n = 18) had higher mortality (3/18, 16.7%) compared to those requiring only non-invasive ventilation (1/4, 25%) or no ventilation (0/8, 0%), though this apparent reversal reflects the very small sample in the non-invasive group (n = 4) where a single death yields a disproportionately high rate; the difference did not reach statistical significance (*p* = 0.31). Older age (>50 years) was associated with higher mortality (3/10, 30.0% vs. 1/20, 5.0%, *p* = 0.07). Time from symptom onset to TPE initiation ≤3 days was associated with higher complete remission rates (18/19, 94.7% vs. 8/11, 72.7%, *p* = 0.14), though not statistically significant.

These findings suggest that TPE was associated with favorable outcomes in myasthenia gravis crisis in this cohort, with 86.7% of patients achieving complete remission and rapid extubation (median 4 days) among survivors. Mortality (13.3%) is comparable to published series of myasthenic crisis treated with TPE (range 5–15%). Older age and invasive mechanical ventilation at presentation may identify patients at higher risk of poor outcomes.

**20. Additional Clinical Outcomes: SLE Disease Activity, Platelet Recovery, Renal Recovery, and Exploratory Biomarkers**

Pre-specified secondary outcomes included SLEDAI-2K score change, time to platelet normalization, dialysis independence, and exploratory biomarker analysis.

Among the 11 SLE patients with available paired SLEDAI-2K assessments, the mean score decreased from 14.2 ± 4.1 at baseline to 6.8 ± 3.6 at discharge, representing a mean reduction of 7.4 points (95% CI 4.8–10.0, *p* < 0.001). This clinically meaningful improvement corresponds to a transition from severe to mild or moderate disease activity.

Regarding platelet recovery in thrombotic thrombocytopenic purpura, the 42 patients who achieved complete remission had a median time to platelet count normalization exceeding 150 × 10³/μL of 6 days (IQR 4–9 days). Patients with a high-risk PLASMIC score of 6 or greater achieved platelet recovery significantly faster than those with lower scores, with median times of 5 days versus 8 days (*p* = 0.03 by log-rank test). Furthermore, every 24-hour delay in TPE initiation was associated with a 1.2-day increase in time to platelet recovery (ρ = 0.41, *p* = 0.01).

For renal recovery, 35.3% of the 34 patients who required dialysis at TPE initiation achieved dialysis independence by hospital discharge. This included 38.9% of patients with antibody-mediated rejection and 31.3% of patients with ANCA-associated vasculitis or anti-GBM disease. The median time to dialysis independence was 14 days (IQR 9–22 days).

An exploratory biomarker substudy of 34 consenting patients with thrombotic microangiopathies measured serum neutrophil gelatinase-associated lipocalin and cystatin C at baseline. NGAL exceeding 300 ng/mL was associated with a 3.2-fold increased risk of requiring more than 7 days to achieve platelet recovery (HR 3.23, 95% CI 1.15–9.09, *p* = 0.02). Cystatin C exceeding 1.5 mg/L correlated strongly with serum creatinine (*r* = 0.71, *p* < 0.001) but did not add independent prognostic information beyond creatinine in multivariate analysis (*p* = 0.34). These exploratory findings suggest NGAL may have utility as an early marker of renal tubular injury in TTP, but larger studies are required for validation.

## Discussion

This single-center, retrospective seven-year real-world analysis provides the most comprehensive evaluation to date of therapeutic plasma exchange across renal, neurologic, hematologic, and metabolic indications in an Egyptian tertiary center; however, the observational design precludes causal inferences. Unlike prior reports that focused on isolated conditions or limited sample sizes, this study was designed to identify disease-specific survival gradients, clinically actionable laboratory thresholds, and independent mortality predictors within a unified prognostic framework. To contextualize the scale of this work, we compared our cohort with previously published Egyptian data. The only other Egyptian adult multi-system TPE cohort, reported by Ghonemy et al. from Tanta University, included 64 patients over a single year [13]. Other Egyptian reports have focused on pediatric intensive care populations [15,16] or addressed isolated indications without comparable cohort breadth [14]. Thus, to our knowledge, the present series of 221 adult patients represents the largest Egyptian multi-system TPE cohort reported to date, filling a critical evidence gap in the regional literature. Globally, most TPE publications remain disease-specific rather than system-wide [25–28]. By integrating survival curves, disease-level mortality mapping, ROC-derived laboratory discrimination, and multivariate modeling within a single analytic framework, this study provides a structurally coherent and internally validated analysis of outcome determinants that transcends single-disease boundaries.

The substantial heterogeneity in baseline laboratory parameters across diagnostic groups warrants comment. As shown in **Table 3**, the whole-cohort standard deviation for hemoglobin (±4.2 g/dL) reflects the mixing of two distinct populations: severely anemic patients in the renal and hematologic groups (mean hemoglobin 8.2–8.7 g/dL) and hematologically normal patients in the neurologic group (mean hemoglobin 13.1 ± 1.9 g/dL). Similarly, the wide standard deviation in renal group platelet count (95.9 ± 107.4 × 10^3^/μL) reflects a bimodal distribution within this group: 41 patients with thrombotic thrombocytopenic purpura (TTP) who presented with severe thrombocytopenia (mean platelet count approximately 30–50 × 10^3^/μL) and were classified under renal indications due to predominant renal involvement (severe acute kidney injury, kidney transplantation, or lupus nephritis), alongside 48 non-TTP renal patients with normal to elevated platelet counts (mean >200 × 10^3^/μL). These cross-group variations, rather than extreme within-group variance, account for the wider whole-cohort standard deviations. Similar patterns of laboratory parameter heterogeneity have been reported in other multi-system TPE cohorts from low- and middle-income countries, where the case mix spans acute autoimmune conditions and chronic organ failure [8]. Importantly, this heterogeneity does not affect the validity of between-group comparisons, as the diagnostic groups were defined a priori based on primary management pathway, and all statistical analyses accounted for baseline differences through multivariable adjustment.

The indication spectrum observed in our cohort, detailed in **Tables 1** and **2**, reflects both the epidemiology of diseases amenable to TPE and referral patterns within the Egyptian healthcare system. Neurologic indications predominated (51.6%), followed by renal (40.3%), hematologic (5.9%), and metabolic disorders (2.2%). Critically, the majority of procedures fulfilled ASFA Category I criteria (77.8%), indicating appropriate evidence-based utilization [4]. This distribution carries biological significance. Neurologic autoimmune conditions such as Guillain–Barré syndrome and myasthenia gravis crisis are characterized by pathogenic circulating antibodies targeting peripheral nerve myelin or acetylcholine receptors. TPE directly removes these immunoglobulins, interrupting complement activation and neuromuscular transmission blockade before irreversible axonal degeneration occurs [29,30]. The rapid clinical improvement observed in these patients aligns with the known kinetics of antibody clearance, where a single 1.0–1.5 plasma volume exchange reduces pathogenic immunoglobulin concentrations by approximately 60–70% [2]. In contrast, renal indications, including antibody-mediated rejection, ANCA-associated vasculitis, and anti-GBM disease, frequently involve immune-complex deposition, endothelial injury, and complement-driven microangiopathy [31]. These processes often reflect more advanced systemic pathology at the time of referral, with established tissue injury that may not be reversible by antibody removal alone. This biological distinction between antibody-mediated functional impairment and immune complex-mediated tissue destruction provides a mechanistic framework for understanding the survival divergence observed in our cohort.

The procedural parameters summarized in **Table 3** demonstrate standardized plasma volumes exchanged, session numbers, and replacement strategies across diagnostic groups. Renal patients required the most intensive treatment (mean 8.2 sessions), reflecting standard protocols for thrombotic thrombocytopenic purpura and antibody-mediated rejection, while neurologic and hematologic patients required fewer sessions (5.6 and 5.8, respectively). The absence of marked intergroup procedural variability strengthens internal validity: outcome differences observed across indications cannot be attributed to inconsistent technical delivery. This homogeneity allows the subsequent survival gradients to be interpreted as disease-driven rather than procedure-driven, a critical distinction for observational research.

Kaplan–Meier analysis (Fig 1) revealed marked survival heterogeneity across diagnostic categories, with neurologic indications exhibiting favorable 90-day survival (89.5%) compared to renal indications (74.2%). The metabolic group demonstrated 100% survival, while the hematologic group showed intermediate outcomes (84.6%). Pairwise comparisons confirmed significantly worse survival in the renal group compared to both neurologic (*p* < 0.001) and metabolic (*p* = 0.008) groups. This survival divergence cannot be attributed to differences in procedural delivery, as documented in Table 3, and instead reflects underlying disease biology. The survival advantage in neurologic conditions likely reflects the reversibility of antibody-mediated conduction block. In Guillain–Barré syndrome, TPE initiated within seven days of symptom onset removes anti-ganglioside antibodies before Wallerian degeneration occurs, preserving axonal integrity and enabling functional recovery [30]. Similarly, in myasthenic crisis, rapid clearance of anti-acetylcholine receptor antibodies restores neuromuscular transmission, often facilitating extubation within days [29]. Our finding that Hughes score ≥4 and delayed TPE initiation (>7 days) independently predicted poor functional outcome (aOR 6.82 and 3.41, respectively, Table 12) directly supports this mechanistic interpretation. Conversely, the poor survival in renal indications reflects the systemic nature of diseases such as ANCA-associated vasculitis and antibody-mediated rejection. These conditions are characterized not only by circulating autoantibodies but also by endothelial activation, complement deposition, and cytokine-mediated organ crosstalk [32]. Once multiorgan dysfunction has supervened, antibody removal alone may be insufficient to reverse established tissue injury.

Fine-grained diagnostic analysis (Table 5) and corresponding mortality mapping (Fig 2) further clarified this gradient. Diffuse alveolar hemorrhage demonstrated the highest mortality (100%), consistent with fulminant pulmonary capillaritis and refractory hypoxemia [33,34]. All nine patients with DAH died despite intervention, with an odds ratio of 56.32 (95% CI 8.91–356.1, *p* < 0.001) compared to the reference category. Pathophysiologically, DAH in the setting of systemic vasculitis or lupus involves immune complex deposition in the pulmonary microvasculature, leading to complement activation, neutrophil recruitment, and capillary destruction [33]. While TPE can remove circulating immune complexes, it cannot reverse established alveolar hemorrhage, intra-alveolar fibrin deposition, or the resultant impairment of gas exchange. All patients were mechanically ventilated at TPE initiation, indicating irreversible alveolar damage; diagnostic and referral delays may have postponed intervention; a high burden of coexistent infection was present in several cases; and the underlying diseases were severe with multi-organ involvement. Based on published literature and pathophysiological reasoning, which implicates immune complex deposition and complement activation as the drivers of alveolar hemorrhage, earlier TPE initiation before the onset of respiratory failure might theoretically improve outcomes by interrupting this cascade before irreversible tissue destruction occurs [33,34]. However, our cohort contained no patients who received TPE before respiratory failure, and therefore we cannot draw empirical conclusions about the optimal timing of TPE in DAH. Prospective studies with early intervention are needed to test this hypothesis. In contrast, thrombotic thrombocytopenic purpura exhibited favorable survival (85.2% response, 14.8% mortality), consistent with the well-established pathophysiology of ADAMTS13 deficiency [32,35]. TPE in TTP serves a dual purpose: removal of inhibitory autoantibodies against ADAMTS13 and replenishment of deficient enzyme via fresh frozen plasma replacement. This dual mechanism restores the physiological cleavage of von Willebrand factor multimers, resolving microangiopathic hemolysis and end-organ ischemia. Antibody-mediated rejection (28.6% mortality) and catastrophic antiphospholipid syndrome (42.9% mortality) occupied intermediate positions in this mortality hierarchy. The concordance between Table 5 and Fig 2 confirms internal coherence between categorical survival and disease-specific mortality signals. Of note, while acute disseminated encephalomyelitis (ADEM) and neuromyelitis optica (NMO) demonstrated favorable overall response rates (75.0% and 80.0%, respectively), none achieved complete remission by the pre-specified criteria requiring complete neurological recovery with normal or stable/resolving MRI findings. This reflects the stringent definition used in this study, which may exceed those in published series where residual MRI abnormalities are often accepted [36]. Clinicians should therefore interpret partial remission in these conditions as a meaningful clinical response, given that residual imaging abnormalities do not preclude functional improvement.

The association between ASFA Category I classification and improved survival (**Table 6**, aOR 0.42 for mortality) provides quantitative validation of the guideline framework within Egyptian tertiary practice. This finding demonstrates that adherence to evidence-based indication stratification is not merely theoretical but translates into a measurable survival advantage, consistent with prior reports from large international registries demonstrating superior outcomes when ASFA guidelines are followed [4,5]. The response gradient observed across ASFA categories (81.4% for Category I, 68.8% for Category II, 64.7% for Category III; p = 0.03) further supports the prognostic utility of this classification system and aligns with the hierarchical evidence base underpinning the ASFA categorization scheme [25]. These data have practical implications for resource-limited settings. In centers where apheresis capacity is constrained, ASFA categorization can guide triage decisions, ensuring that patients most likely to benefit, those with Category I indications, receive priority access, an approach supported by health economic analyses from other low- and middle-income countries [17]. Conversely, Category III indications may warrant more careful consideration, particularly when competing demands for limited apheresis slots exist.

The independent predictors of mortality identified in multivariate analysis () confirm that organ failure, rather than procedural variables, is the dominant determinant of outcome. Mechanical ventilation emerged as the strongest predictor (aOR 5.22, 95% CI 2.31–11.80, *p* < 0.001), likely reflecting systemic inflammatory amplification and advanced organ crosstalk, particularly in renal and vasculitic subgroups. This finding aligns with studies from the World Apheresis Registry showing that respiratory failure at TPE initiation confers similarly elevated mortality risk across multiple indications [21]. Ventilatory dependence appears to mark a transition from reversible immunologic pathology to established multiorgan dysfunction, a concept supported by investigations into the timing of TPE in critical illness [6]. Severe renal impairment (creatinine >2.5 mg/dL) independently predicted mortality with a nearly four-fold increased odds (aOR 3.89), consistent with large cohort studies identifying acute kidney injury as a primary driver of poor outcomes in patients receiving extracorporeal therapies [37]. Patients in the renal diagnostic group faced a more than threefold increased odds of death compared to those in the neurologic group (aOR 3.41), a disparity reflecting both the severity of underlying renal disorders and the intensive, prolonged TPE regimens required for their management. Severe anemia (hemoglobin <8 g/dL) was associated with a nearly threefold increased mortality (aOR 2.67), serving as a marker of disease severity, whether from hemolysis in thrombotic thrombocytopenic purpura, pulmonary hemorrhage in diffuse alveolar hemorrhage, or chronic disease in renal failure, and has been similarly identified as a prognostic factor in critically ill populations requiring plasma exchange [8]. To address potential overfitting concerns, given the events-per-variable ratio of 6.4, a sensitivity analysis using LASSO regression with 10-fold cross-validation was performed. LASSO selected the identical five predictors (mechanical ventilation, creatinine >2.5 mg/dL, renal diagnostic group, hemoglobin <8 g/dL, and ASFA category I protective), confirming the robustness of the primary model.

This study employed two complementary analytical approaches for mortality assessment. Logistic regression was the primary method, identifying predictors of 90-day mortality as a binary outcome, which directly answers the clinical question: Which patients are at risk of death following TPE? Cox proportional hazards regression was performed as a secondary analysis to determine whether these same predictors were associated with earlier mortality (shorter survival time). The consistency of predictors across both models, mechanical ventilation, creatinine >2.5 mg/dL, renal diagnostic group, hemoglobin <8 g/dL, and ASFA category I (protective), supports the robustness of these findings. Notably, the hazard ratios from Cox regression were directionally and proportionally consistent with the odds ratios from logistic regression (Table 10), confirming that these risk factors influence both the likelihood and the timing of mortality. This dual analytical approach strengthens causal inference in this observational cohort.

Receiver operating characteristic curve analysis (Fig 3) demonstrated the discriminative capacity of three continuous laboratory parameters independently associated with mortality. Serum creatinine demonstrated the strongest discriminative ability (AUC 0.81, 95% CI 0.74–0.88), with an optimal cut-off of 2.5 mg/dL yielding sensitivity of 71.9% and specificity of 78.4%. This threshold aligns with previously published cut-offs identifying high-risk patients with thrombotic microangiopathy and vasculitis [31,37]. After bootstrap validation (1,000 replicates), the optimism-corrected AUC was 0.79, indicating minimal overfitting. Hemoglobin showed good discriminative performance (AUC 0.73, 95% CI 0.65–0.81), with an optimal cut-off of 8.0 g/dL (sensitivity 68.8%, specificity 70.1%) and optimism-corrected AUC of 0.71, consistent with studies identifying severe anemia as a marker of disease severity in autoimmune and hematologic conditions [35]. Platelet count demonstrated moderate discriminative ability (AUC 0.67, 95% CI 0.58–0.76), with optimal cut-off of 50 × 10^3^/μL (sensitivity 62.5%, specificity 65.3%) and optimism-corrected AUC of 0.65. The lower discriminative performance of platelet count reflects its bimodal distribution, highly predictive in thrombotic thrombocytopenic purpura but less relevant in other diagnostic groups, a pattern observed in prior analyses of the PLASMIC score [9,22]. These ROC-derived thresholds provide clinically interpretable bedside tools for early risk stratification. A patient presenting with the combination of creatinine exceeding 2.5 mg/dL, hemoglobin below 8.0 g/dL, and platelet count below 50 × 10^3^/μL had an estimated mortality risk exceeding 60% in our cohort, information that may guide intensity of care, adjunctive therapy, and prognostic counseling, though external validation remains necessary before widespread implementation.

In the TTP subgroup (n = 54), multivariate analysis identified three independent predictors of complete remission (Table 9). PLASMIC score ≥6 was the strongest predictor (aOR 3.89, 95% CI 1.45–10.43, *p* = 0.007), validating this score as an effective tool for identifying TTP patients most likely to benefit from TPE, even in resource-limited settings where ADAMTS13 testing may not be immediately available. This finding aligns closely with the original PLASMIC score derivation and validation cohorts, which demonstrated similar odds ratios for complete response in high-risk patients [9,22]. Time to TPE initiation ≤2 days conferred a 2.8-fold increased odds of complete remission (aOR 2.76, 95% CI 1.02–7.45, *p* = 0.045). Each day of delay reduces the probability of platelet count normalization and increases the risk of irreversible end-organ damage. Every 24-hour delay in TPE initiation reduced the odds of complete remission by approximately 15%, consistent with prior studies demonstrating that delayed TPE initiation in TTP is associated with increased mortality and treatment-refractory disease [35]. ADAMTS13 activity <10% showed a strong association that approached but did not reach statistical significance (aOR 2.34, *p* = 0.084), with wide confidence intervals reflecting limited testing availability (63.0% of patients). In settings where ADAMTS13 testing is routinely available, severe deficiency has been consistently shown to predict treatment response [38], suggesting that our finding would likely achieve significance with complete data. These findings underscore the urgency of TPE initiation in suspected TTP and support protocols for emergency apheresis activation. Institutions should establish rapid referral pathways and maintain 24/7 apheresis availability for this time-sensitive indication, as recommended by international guidelines [4].

A key practical insight from this study is that ADAMTS13 testing, often unavailable or delayed in resource-limited settings, may not be necessary for urgent TPE decision-making. The PLASMIC score, calculable from routine laboratory tests within hours of presentation, was a robust predictor of complete remission regardless of ADAMTS13 data availability (aOR 3.89, p = 0.007). Moreover, time to TPE initiation ≤2 days was independently associated with complete remission (aOR 2.76, *p* = 0.045), reinforcing that treatment urgency should not await confirmatory ADAMTS13 results. Sensitivity analyses using multiple imputation and models excluding ADAMTS13 produced consistent findings (PLASMIC score aOR range 3.67–3.92; time to TPE aOR range 2.59–2.81), supporting the robustness of these predictors despite missing ADAMTS13 data in 37% of patients. These findings support a simplified, resource-appropriate algorithm for TTP management in low- and middle-income countries: initiate TPE emergently based on PLASMIC score ≥6 and clinical suspicion, without delaying for ADAMTS13 confirmation. Despite the events-per-variable ratio of 3.9 in the complete-case analysis, the consistency of findings across three sensitivity analyses supports the robustness of the primary predictors.

Among 54 patients with Guillain–Barré syndrome, 24.1% had a poor functional outcome at discharge (Hughes score ≥4). Multivariate analysis (Table 12) identified three independent predictors. Hughes score at admission ≥4 was the strongest predictor (aOR 6.82, 95% CI 2.45–18.97, *p* < 0.001), confirming that presenting severity is the dominant determinant of functional recovery. This finding is consistent with large international GBS cohorts, which have consistently identified baseline disability score as the most important prognostic factor [39,40]. Time from symptom onset to TPE initiation exceeding seven days conferred a 3.4-fold increased odds of poor outcome (aOR 3.41, 95% CI 1.28–9.08, *p* = 0.014). Each day of delay reduces the probability of independent ambulation, supporting urgent TPE referral upon diagnosis. The seven-day window aligns with randomized trial data demonstrating maximal benefit when TPE is initiated early in the disease course [41]. Age greater than 50 years was associated with 2.9-fold increased odds (aOR 2.89, 95% CI 1.06–7.88, *p* = 0.038), consistent with age-related impairment of axonal regeneration and neural repair mechanisms documented in longitudinal studies of GBS recovery [42]. These findings have direct clinical implications: GBS patients presenting with severe weakness should commence TPE without delay, ideally within seven days of symptom onset. Older patients may warrant consideration of combination therapy or extended TPE courses, though this requires prospective evaluation. The predictive strength of admission Hughes score ≥4 for poor functional outcome (aOR 6.82) merits specific comment. While patients admitted with Hughes score ≥4 could theoretically improve to Hughes 3 and still be classified as poor outcome, this reflects clinical reality rather than statistical circularity: patients who present with severe weakness (bedridden or ventilated) rarely achieve full independent ambulation (Hughes ≤2) within the 90-day follow-up period. Those who improve to Hughes 3 represent meaningful partial recovery but remain unable to walk independently, a clinically relevant endpoint. The high odds ratio (6.82) quantifies the prognostic value of presenting severity, consistent with large international GBS cohorts where baseline disability score is the strongest predictor of long-term functional outcome [39,40]. This finding supports early risk stratification and aggressive management for patients presenting with severe weakness.

Importantly, the TTP complete remission model (EPV 3.9) and GBS poor outcome model (EPV ~ 2.2) had events-per-variable ratios below conventional thresholds. These findings are therefore considered exploratory and hypothesis-generating. They should not be interpreted as confirmatory or directly translated into clinical practice without validation in larger independent cohorts. Confirmatory studies with larger sample sizes are needed before these results can be translated into clinical practice guidelines. The outcomes of myasthenia gravis crisis in this cohort merit specific discussion. The complete remission rate of 86.7% and mortality of 13.3% are consistent with published series of myasthenic crisis treated with TPE, which report response rates ranging from 75% to 90% and mortality from 5% to 15% [29,30]. The median time to extubation of 4 days compares favorably with reports of 5–7 days, likely reflecting early TPE initiation and aggressive supportive care. Notably, patients who received TPE within 3 days of crisis onset had a higher complete remission rate (94.7% vs. 72.7%), supporting the importance of urgent intervention in myasthenic crisis, similar to the time-sensitive benefits observed in TTP and GBS. Older age (>50 years) and requirement for invasive mechanical ventilation at presentation were associated with higher mortality, identifying a high-risk phenotype that may benefit from more intensive immunosuppression or combined therapy with intravenous immunoglobulin. These findings confirm TPE as first-line therapy for myasthenic crisis (ASFA category I) and reinforce the guideline recommendation for early initiation [4].

Propensity score-matched analysis (Table 11) demonstrated that after rigorous adjustment for confounders, replacement fluid type (FFP vs. albumin) was not independently associated with mortality or clinical response. Forty-two patients receiving FFP were successfully matched to 42 patients receiving albumin based on key baseline covariates. Unadjusted analysis suggested significantly higher mortality in FFP-treated patients (23.8% vs. 10.7%, *p* = 0.012), reflecting confounding by indication; FFP was preferentially used in sicker patients with thrombotic thrombocytopenic purpura, diffuse alveolar hemorrhage, and antibody-mediated rejection. After matching, no significant difference in mortality was observed (16.7% vs. 14.3%, *p* = 0.76), and clinical response rates were comparable (73.8% vs. 76.2%, *p* = 0.80). This finding is consistent with systematic reviews demonstrating equivalent outcomes when fluids are used according to indication-specific guidelines [36,38]. Fluid selection should therefore follow ASFA recommendations based on disease pathophysiology rather than perceived superiority [4].

Propensity score-matched analysis for vascular access types matched all 11 patients with permanent access (arteriovenous fistulas) to 11 catheter patients (1:1 matching), reflecting the low prevalence of permanent access in acute TPE (5%). While temporary catheters are associated with a small but significant risk of bloodstream infections (7.1% vs. 0%, *p* = 0.04), their overall safety profile is acceptable and does not independently predict mortality or treatment failure. After matching, no significant difference in overall complication rates was observed between catheter and fistula groups (33.3% vs. 27.3%, *p* = 0.48). Insertion-site bleeding, access thrombosis, allergic reactions, and hypotension did not differ significantly between groups. These findings align with registry data from the World Apheresis Association, which reports similar complication profiles for temporary vascular access in acute apheresis [21,43]. The choice of vascular access should prioritize timely TPE initiation over theoretical infection risks in acutely ill patients, a principle supported by clinical practice guidelines emphasizing that delayed therapy poses greater risk than catheter-related complications in time-sensitive indications [4].

The adverse event profile observed in our cohort (Table 4) confirms the safety and feasibility of TPE delivery within a tertiary Egyptian setting. Overall, 77.9% of patients demonstrated clinical response, with 93.0% of responders achieving complete remission and an all-cause mortality of 14.5%. The procedure was well-tolerated, with 62.8% of sessions completed without adverse events. At the patient level, 37.2% experienced at least one adverse event: hypotension in 11.8%, allergic reactions in 9.5%, muscle cramps in 3.6%, and inter-session infections in 11.7%. All documented adverse events were CTCAE grade 1–2 (mild to moderate) in severity and resolved with conservative management; no session required premature termination. The per-session event rates (hypotension 2.4%, allergic reactions 1.9%, muscle cramps 0.7%) compare favorably with international registry data from the World Apheresis Association (hypotension 3–5%, allergic reactions 2–4%) [21] and recent multicenter cohorts [5,8], demonstrating that with appropriate protocols and trained personnel, TPE can be performed safely even in resource-limited environments. The higher rate of inter-session infections (11.7% of patients; 2.4% per session) reflects the cumulative immunosuppressive effect of repeated plasma exchange, particularly immunoglobulin depletion, in the context of severe underlying disease states in a resource-limited setting, underscoring the importance of infection surveillance and prophylactic measures as emphasized in published consensus guidelines [44]. This rate is higher than some international reports (5–8% at patient level), which may reflect differences in patient acuity, infection prevention protocols, and healthcare infrastructure.

Pre-specified secondary outcomes provided complementary insights. Among SLE patients with available paired assessments, SLEDAI-2K scores decreased from 14.2 ± 4.1 at baseline to 6.8 ± 3.6 at discharge (mean reduction 7.4 points, 95% CI 4.8–10.0, *p* < 0.001), representing a clinically meaningful transition from severe to mild or moderate disease activity. In TTP patients achieving complete remission, the median time to platelet count normalization was 6 days (IQR 4–9 days). Patients with high-risk PLASMIC score (≥6) achieved platelet recovery significantly faster than those with lower scores (median 5 vs. 8 days, *p* = 0.03), and every 24-hour delay in TPE initiation was associated with a 1.2-day increase in time to platelet recovery (ρ = 0.41, *p* = 0.01). For renal recovery, 35.3% of patients requiring dialysis at TPE initiation achieved dialysis independence by hospital discharge, including 38.9% of patients with antibody-mediated rejection and 31.3% of patients with ANCA-associated vasculitis or anti-GBM disease, with a median time to dialysis independence of 14 days (IQR 9–22 days). An exploratory biomarker substudy found that NGAL >300 ng/mL was associated with a 3.2-fold increased risk of requiring more than 7 days to achieve platelet recovery (HR 3.23, 95% CI 1.15–9.09, *p* = 0.02), suggesting potential utility as an early marker of renal tubular injury in TTP, though larger studies are required for validation. Elevated NGAL (>300 ng/mL) reflects acute kidney injury in TTP, and its association with slower platelet recovery is biologically plausible: renal tubular damage may serve as a marker of more severe systemic microangiopathy, delayed ADAMTS13 recovery, or more aggressive disease requiring longer TPE courses [35]. This finding suggests that NGAL could serve as an early prognostic biomarker for treatment-refractory TTP, though prospective validation is needed.

Synthesizing the multivariate regression, ROC curve analysis, Kaplan–Meier survival estimates, and indication-specific mortality data, we propose a three-tier risk stratification model for patients undergoing therapeutic plasma exchange. Tier 1 (high risk, estimated mortality >25%) includes diffuse alveolar hemorrhage, which carried 100% mortality in our cohort consistent with published series documenting uniformly poor outcomes once respiratory failure supervenes [33,34]; antibody-mediated kidney transplant rejection (28.6% mortality), aligning with reports of poor prognosis in refractory AMR [31]; and catastrophic antiphospholipid syndrome (42.9% mortality), reflecting the high mortality associated with this fulminant microangiopathic condition [35]. Systemic lupus erythematosus overall (52.2% mortality) is also included in Tier 1, though its mortality is driven predominantly by diffuse alveolar hemorrhage and catastrophic antiphospholipid syndrome; patients with isolated neuropsychiatric involvement have more favorable outcomes (Table 7). Additionally, patients in the renal diagnostic group who require mechanical ventilation and present with both severe renal impairment (creatinine >2.5 mg/dL) and profound anemia (hemoglobin <8 g/dL) have an estimated mortality exceeding 60% based on our multivariate model, a finding consistent with studies demonstrating that multiorgan failure, particularly the combination of respiratory and renal dysfunction, confers additive mortality risk in critically ill populations requiring extracorporeal therapies [32,37]. Tier 2 (intermediate risk, estimated mortality 10–20%) encompasses thrombotic thrombocytopenic purpura (14.8% mortality), with outcomes comparable to those reported in large TTP registries when TPE is initiated promptly [9,22,35]; Guillain–Barré syndrome with severe presenting weakness (Hughes score ≥4) or delayed TPE initiation beyond seven days (13.0% mortality), aligning with established prognostic scores identifying these as key determinants of poor functional recovery [39–42]; myasthenia gravis crisis with ventilator dependence (13.3% mortality), consistent with published series on myasthenic crisis outcomes [29]; and acute disseminated encephalomyelitis (16.7% mortality), which falls within the range reported in pediatric and adult ADEM cohorts [36]. Tier 3 (low risk, estimated mortality <5%) includes autoimmune hemolytic anemia, neuromyelitis optica, pre-kidney transplant desensitization, severe hypertriglyceridemia, chronic inflammatory demyelinating polyneuropathy, and transverse myelitis—all of which demonstrated zero mortality in our cohort, consistent with the generally favorable prognosis of these conditions when TPE is used according to established indications [4,43]. Hyperviscosity syndrome showed low mortality (11.1%), but this was driven by a single elderly patient with advanced multiple myeloma and does not reflect the typical prognosis of this indication.

This model provides a clinically intuitive framework for risk stratification, prognostic counseling, and resource allocation. If prospectively validated, patients in Tier 1 could be considered for intensive care unit admission, multidisciplinary consultation, and consideration of additional immunosuppressive or supportive therapies, as recommended in guidelines for managing high-risk patients requiring TPE [4,44]. Pending external validation, patients in Tier 2 may warrant prompt TPE initiation and vigilant monitoring for complications, with attention to time-sensitive intervention windows identified in our analyses and supported by the literature [30,35]. In our cohort, Tier 3 had no observed deaths in this cohort across all indications in this category; however, the absence of observed deaths in this small subgroup requires confirmation in independent cohorts before such prognostic interpretation.

These findings may have potential clinical implications, although prospective validation is required. Patients with renal indications, particularly those requiring mechanical ventilation or with creatinine >2.5 mg/dL, may warrant closer monitoring and early referral; these implications require prospective evaluation. In TTP and GBS, each additional 24-hour delay in TPE initiation was associated with lower odds of complete remission or functional recovery in this cohort. Institutions should consider establishing rapid referral pathways and maintaining 24/7 apheresis availability. The ROC-derived thresholds provide exploratory, clinically interpretable markers for early risk stratification in this cohort. A patient presenting with the combination of creatinine exceeding 2.5 mg/dL, hemoglobin below 8.0 g/dL, and platelet count below 50 × 10^3^/μL had an estimated mortality risk exceeding 60% in our cohort, information that may inform exploratory risk assessment and prognostic counseling, although external validation is required before clinical application. The three-tier risk model may provide a framework for exploratory prognostic stratification, pending external validation.

The comparison of fresh frozen plasma versus albumin as replacement fluid warrants specific comment regarding methodological rigor. Propensity score matching achieved balance on all measured covariates, but the 50% match rate for FFP recipients initially raises questions about generalizability. However, this limited overlap is expected and, importantly, validates the appropriateness of the matching approach. Complete overlap would have indicated that the two groups were clinically interchangeable, which they were not by design: FFP was reserved for critically ill patients with thrombotic thrombocytopenic purpura, diffuse alveolar hemorrhage, and antibody-mediated rejection, while albumin was used for hemodynamically stable neurologic and metabolic indications [4,8]. The fact that only 50% of FFP patients could be matched confirms that the unmatched patients were fundamentally different; they were the sickest individuals requiring FFP for life-saving replacement of deficient plasma components. The matched sample therefore represents the subset of patients with sufficient covariate overlap for adjusted comparison; however, residual confounding cannot be excluded. The consistency of findings across all three sensitivity analyses supports the conclusion that replacement fluid type was not independently associated with outcome after adjustment for measured confounders. We further acknowledge that the findings may not generalize to the 50% of FFP recipients who could not be matched, the sickest patients requiring FFP for life-saving factor replacement. The observed outcomes in this unmatched subgroup may differ from those in the matched sample, and our conclusions should therefore be interpreted as applicable to the subset of patients where clinical equipoise existed, rather than to all FFP recipients. Critically, three sensitivity analyses, multivariable adjustment in the full cohort, inverse probability of treatment weighting, and subgroup analysis restricted to ASFA category I indications- all yielded consistent findings. The convergence of results across multiple analytical approaches, each with different assumptions and strengths, provides strong evidence that replacement fluid type is not an independent determinant of outcome when ASFA guidelines are followed [36,38]. This methodological triangulation strengthens confidence in our conclusion that fluid selection should be guided by disease-specific pathophysiology rather than perceived superiority of one fluid over another [4].

Several limitations warrant careful consideration. First, the single-center design may limit generalizability to other healthcare systems with different patient demographics, referral patterns, or intensive care capacities. The pooling of diseases across renal, neurologic, hematologic, and metabolic categories introduces substantial clinical heterogeneity. While this approach was deliberately chosen to reflect real-world TPE practice at a tertiary referral center and allows identification of indication-specific outcome differences, it also complicates interpretation of pooled estimates. We addressed this through disease-specific subgroup analyses, multivariate adjustment for diagnostic group, stratified reporting of outcomes by group (Tables 3 and 6), and disease-specific response criteria. Nevertheless, residual confounding due to unmeasured disease-specific factors cannot be excluded. ***Second***, although the cohort size is substantial for a regional study (n = 221), certain diagnostic subgroups were relatively small (e.g., CIDP, transverse myelitis, n = 2–3), potentially limiting statistical power and inflating confidence intervals in subgroup analyses. The small number of patients in certain ASFA categories (Category III, n = 17) also limited our ability to perform detailed within-category analyses. Additionally, multiple subgroup analyses were performed without adjustment for multiple comparisons, as these analyses were explicitly exploratory. This increases the risk of type I error; therefore, all subgroup findings should be considered hypothesis-generating and require confirmation in independent cohorts. ***Third***, despite internal validation of ROC-derived cut-offs using bootstrap resampling (1,000 replicates), external validation in independent cohorts is necessary before widespread clinical implementation. ***Fourth***, follow-up was restricted to 90-day outcomes, precluding assessment of long-term survival, relapse rates, or quality-of-life measures. ***Fifth***, residual confounding inherent to observational designs cannot be entirely excluded, particularly in relation to disease severity and timing of TPE initiation. The retrospective design also limited the availability of certain laboratory parameters, including ADAMTS13 testing which was available in only 63% of TTP patients (37% missing). Though sensitivity analyses confirmed the robustness of the PLASMIC score and treatment urgency as predictors, this missing data represents a limitation and may affect the generalizability of the TTP subgroup findings. Standardized severity scores were not uniformly available. Additionally, follow-up for neurologic outcomes was restricted to 90 days, which may underestimate the proportion of Guillain–Barré syndrome patients achieving independent ambulation, given that functional recovery in GBS often continues for 6–12 months [39,42]. Discharge Hughes scores should therefore be interpreted as reflecting early functional status rather than final long-term outcome. Similarly, myasthenia gravis crisis recovery may extend beyond the 90-day window, and longer follow-up would provide a more complete assessment of treatment durability. ***Sixth***, the three-tier risk model was developed post-hoc in a single-center cohort without external validation. Bootstrap resampling (1,000 replicates) was performed for the underlying statistical models and component predictors, but this does not constitute validation of the three-tier framework itself. The framework should therefore be considered exploratory and hypothesis-generating; it is not validated for clinical use and should not guide patient management decisions until prospectively validated in independent cohorts. ***Seventh***, IRB approval for this study was obtained retrospectively on 27/03/2022, while data collection for the earliest patients (01/01/2016–01/12/2022) had already occurred as part of routine clinical care. While the Zagazig University IRB determined this was permissible under Egyptian regulations (Ministerial Decree 296/2021) for minimal-risk retrospective chart reviews involving no direct patient contact, and while a waiver of informed consent was granted, some journals require prospective ethics approval. The lack of prospective IRB oversight for the earliest years of data collection represents a methodological limitation. However, all data were derived from routine clinical care; no patients were contacted, no interventions were modified for research purposes, and all data were anonymized before analysis, minimizing any potential ethical concern. Readers should interpret the findings in light of this retrospective approval.

The methodological insights from this seven-year real-world cohort suggest several directions for future research. First, the limited overlap between FFP and albumin recipients in propensity score matching, reflecting appropriate guideline-driven fluid selection, highlights the need for prospective registries with standardized data collection across multiple Egyptian centers [4,8]. Such registries would enable larger sample sizes and better characterization of subgroups where clinical equipoise exists for comparative effectiveness research. Second, the convergence of findings across matching, multivariable adjustment, and inverse probability of treatment weighting in this study supports the use of multiple analytical approaches to address confounding by indication in observational TPE research [36,38]. Future studies should prespecify such sensitivity analyses to strengthen causal inference. Third, the three-tier risk stratification model proposed here requires external validation in independent cohorts, ideally from other low- and middle-income countries where TPE access remains limited [6,17]. Fourth, biomarker-guided studies, particularly evaluating neutrophil gelatinase-associated lipocalin (NGAL) as an early marker of renal recovery in thrombotic thrombocytopenic purpura and other thrombotic microangiopathies, warrant prospective evaluation to identify patients most likely to benefit from adjunctive therapies. Finally, cost-effectiveness analyses comparing TPE to intravenous immunoglobulin in resource-limited settings are urgently needed to inform health policy and resource allocation [17].

## Conclusions

In this retrospective, single-center seven-year real-world cohort, the largest adult multi-system TPE experience reported from Egypt to date, clinical outcomes were more strongly associated with underlying disease biology and baseline organ dysfunction rather than procedural variables. Neurologic and thrombotic microangiopathic indications demonstrated high responsiveness when treated early, whereas renal disorders and ventilatory dependence were associated with increased mortality. Mechanical ventilation, elevated creatinine, and severe anemia were independently associated with adverse outcomes, while ASFA Category I indications were associated with lower mortality. These findings suggest the importance of timely, guideline-concordant TPE initiation and structured risk stratification to optimize patient selection and resource allocation; however, the retrospective, single-center design precludes causal inferences. Collectively, this study provides regional observational evidence supporting further evaluation of indication-specific, severity-guided TPE strategies in tertiary care practice, with prospective validation required to confirm these findings. The proposed three-tier risk model, however, should be considered hypothesis-generating and requires prospective external validation before clinical application.
