# Supplementary material for: Therapeutic plasma exchange across multiple organ systems in an Egyptian tertiary center: A seven-year real-world cohort study
Source: PLoS One. 2026 Sep 11;21(9):e0358069. doi: 10.1371/journal.pone.0358069 (PMC13567810; doi:10.1371/journal.pone.0358069)
Supplement: Supplementary file 1 [file pone.0358069.s002.docx]

**Point by Point Response**

**Response to Editorial Comments PLOS ONE Style Requirements**

**Editorial Comment #1**

**Journal Requirements:**

“ Please ensure that your manuscript meets PLOS ONE's style requirements, including those for file naming. The PLOS ONE style templates can be found at:

- <https://journals.plos.org/plosone/s/file?id=wjVg/PLOSOne_formatting_sample_main_body.pdf>
- <https://journals.plos.org/plosone/s/file?id=ba62/PLOSOne_formatting_sample_title_authors_affiliations.pdf> . ”

**Author Response:**

We have carefully reviewed the PLOS ONE style templates and guidelines. We have reformatted our manuscript to meet all requirements. Specifically, we have:

1. **File Format:** Confirmed the manuscript is in DOCX format as required
2. **Font:** Used Arial 11pt font throughout (standard font)
3. **Spacing:** Set to double spacing throughout the manuscript
4. **Page Numbers:** Added page numbers to all pages
5. **Line Numbers:** Added continuous line numbers throughout the manuscript (not restarting on each page)
6. **Title Page:** Formatted as the first page with all required elements (title, authors, affiliations, corresponding author, short title)
7. **Section Headers:** Used consistent formatting for all section headers
8. **No Footnotes:** Verified no footnotes are present; all content is in main text or references
9. **Single Column:** Manuscript is in single column format
10. **Reference Style:** Verified references follow Vancouver style
11. **File Naming:** Renamed all files according to PLOS ONE conventions

**Revised Manuscript Section:**

**No specific text revisions required for this comment.** The entire manuscript has been reformatted to comply with PLOS ONE style requirements. The following changes have been made throughout:

| Requirement | Original Status | Revised Status |
| --- | --- | --- |
| Double spacing | Checked | Confirmed double spacing |
| Page numbers | Missing | Added to all pages |
| Continuous line numbers | Missing | Added throughout |
| File naming | Non-standard | Renamed per PLOS ONE conventions |
| Abstract headers | Present | **NO CHANGE NEEDED** (allowed by PLOS ONE) |

**Editorial Comment #2**

**Data Availability Statement:**

“ We note that you have indicated that there are restrictions to data sharing for this study. For studies involving human research participant data or other sensitive data, we encourage authors to share de-identified or anonymized data. However, when data cannot be publicly shared for ethical reasons, we allow authors to make their data sets available upon request. For information on unacceptable data access restrictions, please see <http://journals.plos.org/plosone/s/data-availability#loc-unacceptable-data-access-restrictions>.

Before we proceed with your manuscript, please address the following prompts:

a) If there are ethical or legal restrictions on sharing a de-identified data set, please explain them in detail (e.g., data contain potentially identifying or sensitive patient information, data are owned by a third-party organization, etc.) and who has imposed them (e.g., a Research Ethics Committee or Institutional Review Board, etc.). Please also provide contact information for a data access committee, ethics committee, or other institutional body to which data requests may be sent.

b) If there are no restrictions, please upload the minimal anonymized data set necessary to replicate your study findings to a stable, public repository and provide us with the relevant URLs, DOIs, or accession numbers.

Please update your Data Availability statement in the submission form accordingly. “

**Author Response:**

We thank the PLOS ONE editorial office for bringing this issue to our attention. We have reviewed the journal's data-sharing requirements and have revised the Data Availability Statement to clearly explain why the individual-level dataset cannot be made publicly available.

The study is based on retrospective clinical records from 221 patients. The underlying individual-level dataset contains demographic, diagnostic, laboratory, treatment-related, and clinical outcome information. Although the analytical data were de-identified, the combination of variables, particularly for patients with uncommon TPE indications, could permit re-identification through linkage with institutional records. The study was conducted under institutional ethics approval and a waiver of informed consent, and the underlying patient-level data are subject to institutional and applicable data-protection restrictions**.**

**Accordingly, we are unable to deposit the individual-level dataset in a public repository or make it freely downloadable. We have therefore provided a detailed Data Availability Statement in the revised manuscript explaining the restriction and specifying the institutional procedure through which scientifically justified data-access requests may be considered, subject to approval by the Zagazig University Institutional Review Board and applicable data-protection requirements.**

We have provided complete contact information for data-access requests, including the Research Ethics Committee Office.

**We understand that PLOS ONE requires an explicit editorial assessment when human participant data cannot be publicly deposited for ethical or legal reasons. We therefore respectfully request that the present case be considered for an exemption from public data deposition under the journal's data-sharing policy. We are willing to provide any additional documentation required by the editorial office to support this exemption**.

**Revised Manuscript Section:**

**Original Data Availability Statement:**

The datasets generated and analyzed during the current study are not publicly available because they contain information that could compromise research participant privacy, and informed consent was waived for this retrospective study. De-identified data may be available from the corresponding author upon reasonable request, subject to approval from the Zagazig University Institutional Review Board and applicable Egyptian data protection regulations.

**Revised Data Availability Statement: (Bold = Add)**

“ **The individual-level dataset generated and analyzed during the current study is not publicly available because it comprises retrospective human participant clinical data subject to institutional ethical and data-protection restrictions. The study was conducted under a waiver of informed consent, and public deposition or unrestricted external transfer of the underlying patient-level data was not authorized. Although the analytical dataset was de-identified, the combination of demographic, diagnostic, laboratory, treatment, and outcome variables may permit re-identification, particularly for patients with uncommon TPE indications, through linkage with institutional records. Accordingly, the individual-level dataset cannot be deposited in a public repository or made freely downloadable. De-identified data may be made available for scientifically justified requests, subject to review and approval by the Zagazig University Institutional Review Board and applicable data-protection requirements. Data-access requests may be submitted to the Research Ethics Committee Office, Zagazig University Faculty of Medicine, Zagazig, Egypt (email: irb@zu.edu.eg; phone: +20 55 230 3125).**"

**Editorial Comment #3**

**Ethics Statement Location:**

“ Your ethics statement should only appear in the Methods section of your manuscript. If your ethics statement is written in any section besides the Methods, please delete it from any other section ”.

**Author Response:**

We have confirmed that the full ethics statement appears exclusively in the Methods section (under "Ethical Considerations"). We have revised the Declarations section to contain only a brief reference to the Methods section, eliminating any duplication.

**Revised Manuscript Section:**

**Original Declarations Section (Ethics Statement – Keep full text):**

This retrospective cohort study was approved by the Institutional Review Board of Zagazig University Faculty of Medicine (ZU-IRB #9357) on 27/03/2022 (initial approval; expired 27/03/2023), with renewal approved on 08/02/2026 (expires 08/02/2027). Data collection and analysis were completed before the initial approval expired on 27/03/2023; the 2026 renewal was obtained solely for manuscript preparation and journal submission requirements, as the study involved no ongoing patient contact or new data collection after the initial approval period. IRB approval was obtained retrospectively, as data from patients treated between 01/01/2016 and 01/12/2022 were extracted from existing medical records created during routine clinical care. The IRB permitted this under Egyptian national regulations (Ministerial Decree 296/2021) for minimal-risk retrospective research involving no direct patient contact.

Informed consent: Because this study involved only the analysis of de-identified, pre-existing medical records, with no direct patient contact or intervention, the requirement for written informed consent was waived by the Zagazig University IRB. No research consent was obtained from any patient or legally authorized representative. The reason written consent could not be obtained was the retrospective nature of the study: contacting all 221 patients (including 32 who died during follow-up) was not feasible without prospective patient contact, which this retrospective design specifically excluded. This determination is consistent with Egyptian national regulations (Ministerial Decree 296/2021, Article 18) and the Declaration of Helsinki (2013 revision), which permit waiver of consent for retrospective research using existing medical records when the research involves no more than minimal risk and cannot practicably be carried out without the waiver.

Data anonymization and confidentiality: Patient data were extracted from electronic medical records and paper-based apheresis logs. Immediately after extraction, data were pseudonymized using unique study identification numbers. Direct identifiers (names, national identification numbers, exact dates of birth) were removed before analysis. The linking file connecting identifiers to study numbers was stored on a password-protected, encrypted hospital server accessible only to the principal investigator.

Confirmation of ethical compliance: The authors confirm that all research was performed in accordance with the ethical principles of the Belmont Report, the Declaration of Helsinki (2013 revision), and Egyptian national regulations for biomedical research.

Human Participants Checklist: The completed PLOS Human Participants Research Checklist is submitted as Supporting Information (S1 Checklist).

**Revised Declarations Section (Ethics Statement - condensed):**

***Ethics approval and consent to participate:*** This study was approved by the Institutional Review Board of Zagazig University Faculty of Medicine (ZU-IRB #9357) as described in the Methods section (Ethical Considerations). **The IRB waived the requirement for written informed consent as detailed in the Methods section*.*** All methods were performed in accordance with relevant guidelines and regulations, including Egyptian national regulations (Ministerial Decree 296/2021) and the Declaration of Helsinki (2013).

**Editorial Comment #4**

**Reviewer-Recommended Citations:**

“ If the reviewer comments include a recommendation to cite specific previously published works, please review and evaluate these publications to determine whether they are relevant and should be cited. There is no requirement to cite these works unless the editor has indicated otherwise. “

**Author Response:**

We have carefully reviewed all reviewer comments to identify any recommendations to cite specific previously published works. **No reviewer recommended any specific citations.**

**Reviewer #1's** comments focused on manuscript length, study design, statistical modeling, and presentation issues. **Reviewer #2's** comments focused on model interpretation, patient classification, propensity score matching, and adverse event reporting. Neither reviewer requested the addition of any particular references.

Therefore, we have not added any new citations in response to this comment. We have verified that our existing reference list is comprehensive and appropriately supports the manuscript content. No additional citations were identified as necessary based on reviewer feedback.

**Revised Manuscript Section:**

**No revisions required.** The existing reference list remains unchanged as no reviewer recommended any specific citations. The reference list in the manuscript is complete and contains all necessary citations to support the content.

**Response to Reviewer #1**

A. **Overall Assessment**

**Reviewer comment:**

*“*This manuscript presents a retrospective seven-year cohort evaluating therapeutic plasma exchange (TPE) in an Egyptian tertiary center. The topic is clinically relevant because real-world evidence from low- and middle-income countries remains limited. The study includes 221 patients and evaluates efficacy, safety, and predictors of outcomes. While the work has merit, substantial revisions are required before publication*.”*

**Response:**
 We thank the reviewer for the positive recognition of our work's clinical relevance and the acknowledgment that real-world evidence from low- and middle-income countries remains limited. We appreciate the reviewer's constructive assessment and have carefully addressed all substantial revisions requested in the subsequent comments.

B. **Major Comments**

**1. Manuscript Length:**

**Reviewer comment:**

*“*The manuscript is considerably longer than necessary. The Introduction, Methods, and Discussion should be reduced by approximately 30%, removing repeated methodological descriptions and excessive technical details*.”*

**Author Response:**

We respectfully acknowledge the reviewer's concern regarding manuscript length, but we would like to provide a rationale for the current structure and explain why many of the details the reviewer may perceive as "excessive" or "repeated" are, in our view, essential to the scientific integrity, reproducibility, and interpretability of this work.

**Our Approach: Comprehensive Rather Than Redundant**

We have carefully reviewed the manuscript for redundancy and have made **targeted reductions of approximately 15–18%** (rather than the full 30% requested) because we believe the current length is justified by several factors:

***1. The Heterogeneity of the Cohort Necessitates Detailed Description***

Unlike disease-specific TPE studies that focus on a single condition (e.g., TTP alone or GBS alone), our cohort spans **renal, neurologic, hematologic, and metabolic indications**—each with distinct pathophysiology, procedural requirements, and outcome measures. This heterogeneity demands:

| Component | Why It Is Necessary |
| --- | --- |
| Detailed disease-specific response criteria | Without these, readers cannot interpret whether "response" means the same thing across GBS, TTP, SLE, and hypertriglyceridemia |
| Stratified baseline characteristics (Table 3) | The profound differences in hemoglobin, platelets, and creatinine across groups are the foundation of our risk model—readers need to see these to understand why outcomes differ |
| Disease-specific outcome tables (Tables 5–8) | Pooling all diseases into a single outcome table would mask the critical finding that TTP and GBS respond very differently from DAH and AMR |

**The heterogeneity is not a flaw; it is the central finding of the study.** The length reflects the complexity of the dataset, not redundancy.

***2. Reproducibility Requires Technical Detail***

We fully agree that manuscripts should avoid unnecessary technical detail. However, we believe that **transparency in methods is essential for reproducibility**, particularly in resource-limited settings where protocols may need to be adapted.

The following technical details are not "excessive"; they are necessary for replication:

| Technical Detail | Why It Is Necessary |
| --- | --- |
| TPE device specifications (membrane vs. centrifugal) | Outcomes may differ by device; readers need to know which devices were used and why |
| Kaplan's equation for plasma volume calculation | This is the standard formula, but variations exist; specifying the exact method ensures consistency in future studies |
| Replacement fluid selection criteria (FFP vs. albumin) | This is a critical clinical decision; readers need to understand the rationale to apply it in practice |
| Vascular access protocol (ultrasound guidance, catheter type) | Catheter-related complications are a major concern; specifying the protocol allows others to achieve similar safety outcomes |
| Adverse event classification (CTCAE v5.0) | Standardized grading ensures comparability with international literature |

**Technical detail is not redundant; it is the foundation of evidence-based practice transfer.**

***3. The Discussion Addresses a Complex Evidence Gap***

The reviewer suggests reducing the Discussion by 30%. However, our Discussion must accomplish several distinct tasks:

| Discussion Component | Why It Is Necessary |
| --- | --- |
| Contextualizing within international literature | This is the first large Egyptian multi-system TPE cohort; readers need to understand how our findings compare to those from North America, Europe, and Asia |
| Explaining disease-specific mechanisms | The pathophysiology of TTP (ADAMTS13) differs fundamentally from GBS (anti-ganglioside antibodies) and SLE (immune complexes); explaining these differences is essential for interpreting why outcomes vary |
| Translating findings to clinical practice | The ROC-derived cut-offs (creatinine >2.5, hemoglobin <8, platelets <50) are actionable; readers need sufficient context to apply them |
| Acknowledging limitations | The retrospective, single-center design and low EPV ratios require careful caveats; minimizing this section would undermine scientific integrity |
| Proposing future directions | The field needs prospective validation and multicenter registries; this is not padding, it is a call to action |

**The Discussion is comprehensive because the evidence gap is wide and the implications are significant.**

***4. The "Repeated Methodological Descriptions" Are Cross-Referenced, Not Duplicated***

Upon reviewing the manuscript, we identified that some methodological descriptions appear to be repeated because:

- **Methods are presented in the main text for accessibility** (for readers who may not consult supplements)
- **Disease-specific criteria are summarized in the main text and detailed in the supplement** (this is standard practice)

We have **merged** the following repetitive descriptions:

| Merged Content | Action Taken |
| --- | --- |
| Laboratory protocols for ADAMTS13, NGAL, and cystatin C | Now consolidated into one paragraph |
| TPE procedure descriptions for membrane vs. centrifugal | Now consolidated with a single reference to the standard protocol |
| Disease-specific response criteria | Now summarized in the main text |

**However, we have not removed all technical detail because doing so would compromise the manuscript's utility as a reference for clinicians in similar resource-limited settings.**

***5. The Manuscript Architecture Follows a Logical Progression***

We have structured the manuscript to guide the reader through:

1. **The problem** (evidence gap in LMICs) → **Introduction**
2. **The approach** (comprehensive cohort study) → **Methods**
3. **The findings** (disease-specific outcomes, predictors, risk model) → **Results**
4. **The implications** (clinical translation, limitations, future directions) → **Discussion**

Each step builds on the previous one. **A further reduction of this magnitude would risk removing information necessary for interpretation and reproducibility**.

**Revised Manuscript**

***Introduction: Removed Redundant Historical Background***

**Original (removed):**

*"*Since its clinical introduction in the mid-twentieth century, TPE has evolved from a niche rescue therapy to an established intervention across multiple medical disciplines, with the American Society for Apheresis (ASFA) currently recognizing 166 graded indications spanning hematology, neurology, nephrology, rheumatology, metabolic disorders, transplant medicine, and critical care [4]."

**Revised (retained only essential statement):**

*"*The American Society for Apheresis (ASFA) currently recognizes 166 graded indications for TPE across multiple disciplines [4]."

***Methods: Merged Laboratory Protocols***

**Original (two separate paragraphs):**

"For patients with suspected thrombotic thrombocytopenic purpura, ADAMTS13 activity and inhibitor titers were measured when clinically available. Samples were collected in 3.2% sodium citrate tubes, centrifuged at 2500 ×g for 15 minutes at 4°C, and plasma was stored at -80°C until batch analysis. ADAMTS13 activity was determined using a fluorescence resonance energy transfer assay (Technozym ADAMTS-13 Activity ELISA, Technoclone, Vienna, Austria). Severe deficiency was defined as activity <10%.

For biomarker substudies (n=34 consenting patients with thrombotic microangiopathies), serum neutrophil gelatinase-associated lipocalin and cystatin C were measured within 24 hours of TPE initiation. Samples were processed within 30 minutes of collection by centrifugation at 2500 rpm for 15 minutes at 4°C (Eppendorf 5702R, Hamburg, Germany). Aliquots were stored at -80°C (Thermo Scientific Forma 900 series, Waltham, MA, USA) until batch analysis. Serum NGAL and cystatin C concentrations were determined using commercially available enzyme-linked immunosorbent assay kits (Sunred Biological Technology Co., Ltd, Shanghai, China), following manufacturer protocols validated per Clinical and Laboratory Standards Institute EP15-A3 guidelines."

**Revised (merged):**

"	For patients with suspected thrombotic thrombocytopenic purpura, ADAMTS13 activity and inhibitor titers were measured when available. Plasma was collected in 3.2% sodium citrate tubes, centrifuged at 2500 ×g for 15 minutes at 4°C, stored at −80°C, and analyzed in batches using a fluorescence resonance energy transfer assay (Technozym ADAMTS-13 Activity ELISA, Technoclone, Vienna, Austria). Severe ADAMTS13 deficiency was defined as activity <10%. In the metabolic group, fasting triglycerides, total cholesterol, LDL cholesterol, and HDL cholesterol were measured by enzymatic colorimetric methods on the Cobas 8000 analyzer. In the biomarker substudy, serum NGAL and cystatin C were measured within 24 hours of TPE initiation in 34 consenting patients with thrombotic microangiopathies. Samples were centrifuged at 2500 rpm for 15 minutes at 4°C, aliquoted, and stored at −80°C until batch analysis. NGAL and cystatin C were measured using commercial ELISA kits (Sunred Biological Technology Co., Ltd., Shanghai, China) according to manufacturer protocols validated per CLSI EP15-A3 guidelines. Absorbance was measured using a BioTek ELx808 microplate reader (Agilent Technologies, Santa Clara, CA, USA). Intra- and inter-assay coefficients of variation were <10% for both assays."

***Discussion: Condensed International Comparison***

**Original (removed):**

" This finding is consistent with systematic reviews and meta-analyses demonstrating equivalent outcomes when fluids are used according to indication-specific guidelines [37,42]. The choice of replacement fluid should therefore be guided by the specific disease indication, as recommended by ASFA guidelines: FFP for conditions requiring factor replacement (TTP, HUS, antibody-mediated rejection, DAH) and albumin for pure antibody removal (GBS, MG, hyperviscosity), rather than by perceived superiority of one fluid over another [4]."

**Revised (retained only essential comparison):**

*"* This finding is consistent with systematic reviews demonstrating equivalent outcomes when fluids are used according to indication-specific guidelines [37,42]. Fluid selection should therefore follow ASFA recommendations based on disease pathophysiology rather than perceived superiority [4]*."*

**2. Study Objectives**

**Reviewer comment:**

*"*The objectives are overly broad. Clearly distinguish primary, secondary, and exploratory objectives*.”*

**Response:**
 We respectfully acknowledge the reviewer's concern but would like to clarify why our objectives are structured as they are and why, in our view, they are appropriate for a comprehensive real-world cohort study rather than "overly broad."

We understand the reviewer's concern that multiple objectives may appear "overly broad." However, we would like to explain why this structure is both necessary and appropriate for a study of this nature.

***1. Our Objectives Reflect the Study's Purpose as a Real-World Cohort***

This is not a narrow, hypothesis-driven study of a single disease. It is a **comprehensive real-world cohort study** designed to characterize TPE practice across multiple organ systems in a setting where no such data previously existed. The objectives are therefore appropriately broad because:

| Component | Why It Is Necessary |
| --- | --- |
| Demographics, indications, and ASFA distribution | This is **descriptive epidemiology**, the foundation of any first-time cohort study in a new setting. Without this, clinicians and policymakers have no baseline understanding of who is receiving TPE and for what reasons |
| Efficacy and safety quantification | This is the **core clinical question**: does TPE work in this setting, and is it safe? |
| Independent predictors of mortality and response | This is the **prognostic question**: which patients are most likely to benefit or suffer harm? |

**The objectives are broad because the evidence gap is wide.** This is the first comprehensive characterization of TPE in the Nile Delta region; narrowing the focus would leave critical questions unanswered.

***2. The "Objectives" and "Outcome Measures" Sections Serve Different Purposes***

We note that the reviewer may have conflated the **study objectives** (the overarching aims of the research) with the **outcome measures** (the specific metrics used to evaluate those aims). These are distinct:

| Section | Purpose | Content |
| --- | --- | --- |
| Introduction (Objectives) | State the overarching aims | Three high-level aims: (1) describe the cohort, (2) quantify efficacy/safety, (3) identify predictors |
| Methods (Outcome Measures) | Define specific metrics | Detailed definitions of primary, secondary, and disease-specific outcomes |

The "outcome measures" section is **not** a list of objectives; it is a detailed operationalization of how each objective will be evaluated. This distinction is standard in clinical research and is required for reproducibility.

***3. We Have Already Categorized Primary, Secondary, and Exploratory Outcomes in the Methods Section***

Upon review of our manuscript, we note that the **primary, secondary, and exploratory outcomes** are already clearly distinguished in the "Outcome Measures" section:

| Category | Outcome | Location in Text |
| --- | --- | --- |
| Primary | Clinical response (complete/partial/no response) | "The primary outcomes were clinical response and all-cause mortality" |
| Primary | All-cause mortality (30-day, 90-day, end of follow-up) | "All-cause mortality was assessed at 30 days, 90 days, and at the end of follow-up" |
| Secondary | Adverse events, ASFA adherence, length of stay, ICU admission, ventilation duration | "Secondary outcomes included adverse events, ASFA guideline adherence rate..." |
| Secondary | Disease-specific outcomes (TTP, GBS, MG, SLE, renal) | "Disease-specific outcome measures included time to platelet count normalization..." |
| Exploratory | NGAL and cystatin C biomarker substudy | "For the NGAL and cystatin C biomarker substudy..." |

**The distinction the reviewer requests exists in the manuscript** in the Methods section rather than the Introduction.

***4.* *The Introduction Objectives Are Intentionally Concise***

The Introduction states three objectives:

1. **To delineate** demographics, indications, and ASFA distribution
2. **To quantify** efficacy and safety with disease-specific stratification
3. **To identify** independent predictors of mortality and response.

These are **clear, distinct, and sequential**. Each objective corresponds to a specific set of analyses:

| Objective | Corresponding Analyses |
| --- | --- |
| Objective 1 (Descriptive) | Tables 1, 2, 3 (patient characteristics, indications, ASFA categories) |
| Objective 2 (Efficacy/Safety) | Tables 4, 5, 6 (response rates, mortality, adverse events by group) |
| Objective 3 (Predictors) | Tables 9, 10, 11, 12 (multivariate models, survival analysis, subgroup models) |

We respectfully submit that this is **not overly broad**; it is **appropriately comprehensive** for a first-time characterization of TPE in a new setting.

While we believe the current structure is scientifically sound, we **agree** with the reviewer that explicit categorization of primary, secondary, and exploratory objectives in the Introduction would improve clarity and readability.

We have therefore revised the Introduction to:

1. **Retain the three overarching aims** (which are appropriate and necessary)
2. **Explicitly label** each aim as primary, secondary, or exploratory
3. **Cross-reference** the corresponding analyses and outcome measures

**Revised Manuscript Section:**

***Original Introduction (Objectives paragraph):***

*"*Our specific objectives were threefold: first, to delineate the demographic characteristics, clinical indications, and ASFA category distribution among 221 patients undergoing TPE across renal, neurologic, hematologic, and metabolic diagnostic groups; second, to quantify treatment efficacy (clinical response rates, remission patterns) and safety (adverse event frequency and severity) with granular, disease-specific stratification; and third, to identify independent predictors of mortality and poor therapeutic response through multivariate regression modeling*."*

***Revised Introduction (with bold additions):***

*"* Our specific objectives were categorized as follows*:* ***Primary Objectives:*** (1) To quantify treatment efficacy (clinical response rates, complete and partial remission patterns) and safety (adverse event frequency and severity) across the full cohort and by diagnostic group; (2) To identify independent predictors of 90-day all-cause mortality and clinical response through multivariate logistic regression and time-to-event (Cox proportional hazards) analyses. ***Secondary Objectives:*** (1) To delineate the demographic characteristics, clinical indications, and ASFA category distribution among patients undergoing TPE across renal, neurologic, hematologic, and metabolic diagnostic groups; (2) To evaluate pre-specified disease-specific outcomes for five subgroups (TTP, GBS, MG crisis, SLE, and DAH); (3) To assess the association of procedural variables (replacement fluid type, vascular access) with outcomes using propensity score matching. ***Exploratory Objectives:*** *(*1) To develop a hypothesis-generating three-tier risk stratification model based on synthesized multivariate and survival data; (2) To evaluate the PLASMIC score and treatment urgency as predictors of complete remission in TTP; (3) To identify predictors of poor functional outcome in GBS; (4) To explore the utility of NGAL as a biomarker in thrombotic microangiopathies (substudy). "

**3. Study Design**

**Reviewer comment:**

*“*The retrospective, single-center nature of the study should be emphasized more consistently, and causal language should be avoided.”

**Response:**
 We thank the reviewer for this important methodological point. We agree that the retrospective, single-center design should be consistently emphasized throughout the manuscript and that causal language should be avoided. We have carefully revised the manuscript to address both concerns.

We fully agree with the reviewer on both points. However, we want to explain how we have approached the revision:

***1.* *Emphasis on Retrospective, Single-Center Design***

We have added explicit acknowledgment of the study's retrospective, single-center design at **multiple points** throughout the manuscript where it is most relevant:

| Location | Original Text | Revised Text |
| --- | --- | --- |
| Abstract | "This cohort study included 221 consecutive patients..." | "**This retrospective** cohort study included 221 consecutive patients..." |
| Methods (first line) | "This cohort study was conducted at..." | "**This retrospective cohort study** was conducted at..." |
| Methods (Study Population) | "Data were derived from a prospectively maintained institutional database..." | "Data were derived from a **retrospectively extracted** prospectively maintained institutional database..." |
| Results | Various descriptive statements | Added "in this cohort" or "in our single-center experience" where appropriate |
| Discussion (first line) | "This seven-year real-world analysis provides..." | "**This single-center, retrospective** seven-year real-world analysis provides..." |
| Discussion (Limitations) | Existing limitations section | Enhanced with clearer emphasis on retrospective, single-center nature |
| Conclusions | "In this seven-year real-world cohort..." | "In this **retrospective, single-center** seven-year real-world cohort..." |

***2. Removal of Causal Language***

We have systematically replaced causal language ("confirming," "validating," "predictive," "demonstrates") with associative language ("suggesting," "supporting," "associated with," "suggests that...may") throughout the manuscript:

| Original Causal Language | Revised Associative Language |
| --- | --- |
| "confirming that presenting severity is the dominant determinant" | "**suggesting that** presenting severity **is associated with** poor outcome" |
| "validating this score as an effective tool" | "**supporting the potential utility of** this score" |
| "demonstrates that TPE is highly effective" | "**suggests that** TPE **may be** highly effective" |
| "predicts poor functional recovery" | "**is associated with** poor functional recovery" |
| "these findings underscore the urgency" | "**these findings suggest** the urgency" |
| "identifies these as key determinants" | "**suggests these may be** key determinants" |
| "provides quantitative validation" | "**provides supportive evidence for**" |
| "confirms the robustness" | "**supports the robustness**" |
| "demonstrating superior outcomes" | "**showing** superior outcomes **in this cohort**" |
| "the strongest predictor" | "**was strongly associated with**" |
| "independently predicted mortality" | "**was independently associated with** mortality" |
| "these findings have direct clinical implications" | "**these findings suggest potential** clinical implications **that require prospective validation**" |

**Revised Manuscript Sections:**

***Abstract (Revised):***

**Original:**
 *"*This cohort study included 221 consecutive patients undergoing TPE (2016–2022) at Zagazig University Hospitals, Egypt*."*

**Revised:**
 *"***This retrospective cohort study** included 221 consecutive patients undergoing TPE (2016–2022) at Zagazig University Hospitals, Egypt*."*

***Methods Section - Study Population (Revised):***

**Original:**
 " This cohort study was conducted at the Nephrology and Apheresis Unit of Zagazig University Hospitals, a major tertiary referral center in the Nile Delta region of Egypt serving a catchment population of approximately ten million inhabitants. Data were derived from a prospectively maintained institutional database (from which data were extracted retrospectively for this analysis) with clinical information, including adverse events, recorded in real-time at the point of care and subsequently extracted for analysis."

**Revised:**
 " **This retrospective cohort study** was conducted at the Nephrology and Apheresis Unit of Zagazig University Hospitals, a major tertiary referral center in the Nile Delta region of Egypt serving a catchment population of approximately ten million inhabitants. Data were derived from a prospectively maintained institutional database **(from which data were extracted retrospectively for this analysis)** with clinical information, including adverse events, recorded in real-time at the point of care and subsequently extracted for analysis."

***Discussion - Opening Paragraph (Revised):***

**Original:**
 " This seven-year real-world analysis provides the most comprehensive evaluation to date of therapeutic plasma exchange across renal, neurologic, hematologic, and metabolic indications in an Egyptian tertiary center."

**Revised:**
 " **This single-center, retrospective** seven-year real-world analysis provides the most comprehensive evaluation to date of therapeutic plasma exchange across renal, neurologic, hematologic, and metabolic indications in an Egyptian tertiary center**; however, the observational design precludes causal inferences.** "

***Language Revisions:***

| Location/Section | Original Text | Revised Text |
| --- | --- | --- |
| SLE Subgroup (Section 7) | "These findings demonstrate that the high overall mortality in this SLE cohort is driven predominantly by the inclusion of patients with diffuse alveolar hemorrhage and catastrophic antiphospholipid syndrome…" | "**These findings suggest that** the high overall mortality in this SLE cohort **was largely attributable to** the inclusion of patients with diffuse alveolar hemorrhage and catastrophic antiphospholipid syndrome…" |
| SLE Subgroup (Section 7) | "These findings confirm that TPE in SLE is reserved for the most severe, life-threatening manifestations and that outcomes are strongly phenotype-dependent." | "**In this cohort,** these findings **suggest that** TPE in SLE **was used primarily for** severe, life-threatening manifestations and that outcomes **varied according to** clinical phenotype." |
| SLE Subgroup (Section 7) | "While TPE may be life-saving in catastrophic antiphospholipid syndrome and thrombotic thrombocytopenic purpura, its efficacy in diffuse alveolar hemorrhage… is limited." | "**While favorable outcomes were observed in some patients with catastrophic antiphospholipid syndrome and thrombotic thrombocytopenic purpura,** patients with diffuse alveolar hemorrhage, particularly those requiring mechanical ventilation, **had poor outcomes.** " |
| MG Crisis (Section 19) | "These findings demonstrate that TPE is highly effective for myasthenia gravis crisis…" | "**These findings suggest that** TPE **was associated with favorable outcomes in** myasthenia gravis crisis **in this cohort…** " |
| MG Crisis (Section 19) | "These findings confirm TPE as first-line therapy for myasthenic crisis…" | "**These findings are consistent with the established role of** TPE as a first-line therapy for myasthenic crisis…" |
| Statistical Modeling (Section 10) | "The concordance between LASSO and the primary enter method logistic regression supports the robustness of the five-predictor mortality model…" | "The concordance between LASSO and the primary enter method logistic regression supports the **stability of the five-predictor mortality model in this dataset**…" |
| Statistical Modeling (Section 10) | "LASSO selected the identical five predictors … confirming the robustness of the primary model." | "LASSO selected the same five predictors … **supporting the stability of** the primary model." |
| Statistical Modeling (Section 10) | "This dual analytical approach strengthens causal inference in this observational cohort." | "This dual analytical approach **strengthens confidence in the consistency of the observed associations** in this observational cohort." |
| Survival Analysis (Section 12) | "...confirming that these risk factors influence both the likelihood and the timing of mortality." | "…**supporting the consistency of their associations with** both the likelihood and timing of mortality." |
| Discussion - Predictors | "The independent predictors of mortality identified in multivariate analysis … confirm that organ failure, rather than procedural variables, is the dominant determinant of outcome." | "The independent predictors of mortality identified in multivariate analysis **suggest that** baseline organ dysfunction, rather than measured procedural variables, **was more strongly associated with** outcome." |
| Discussion - Mechanical Ventilation | "This finding underscores that respiratory failure at the time of TPE initiation represents established, irreversible organ damage and portends a poor prognosis…" | "This finding **suggests that** respiratory failure at the time of TPE initiation **may indicate** advanced organ dysfunction and **was associated with** poorer prognosis…" |
| Discussion - Renal Impairment | "This reflects either advanced intrinsic renal disease or prolonged ischemic injury…" | "**This may reflect** advanced intrinsic renal disease or prolonged ischemic injury…" |
| Discussion - Renal Group Disparity | "This disparity reflects both the severity of underlying renal disorders and the intensive, prolonged TPE regimens required…" | "**This disparity may reflect** differences in underlying disease severity and treatment requirements…" |
| Discussion - Treatment Sessions | "A treatment course of five or more TPE sessions was associated with nearly two-fold increased odds of response (aOR 1.89…), reflecting the importance of treatment adequacy." | "A treatment course of five or more TPE sessions was associated with nearly two-fold increased odds of response (aOR 1.89…); **however, this association should not be interpreted as evidence that a greater number of sessions improves response.** " |
| Discussion - Replacement Fluid | "The convergence of results across multiple analytical approaches … provides strong evidence that replacement fluid type is not an independent determinant of outcome…" | "**The consistency of results** across multiple analytical approaches **suggests that** replacement fluid type **was not independently associated with** outcome after adjustment for measured confounders." |
| Discussion - Replacement Fluid | "The consistency of findings across all three sensitivity analyses strongly supports the conclusion that replacement fluid type itself does not independently determine outcome…" | "The consistency of findings across all three sensitivity analyses **supports the conclusion that** replacement fluid type **was not independently associated with** outcome after adjustment for measured confounders." |
| Discussion - Methodological Triangulation | "This methodological triangulation strengthens confidence in our conclusion that fluid selection should be guided…" | "This methodological triangulation **increases confidence in the consistency of the observed association** and supports **interpretation of** fluid selection according to disease-specific indications…" |
| Discussion - Propensity Score | "The matched sample, therefore, represents the subset of patients where equipoise existed, allowing unbiased estimation of the treatment effect." | "The matched sample therefore represents the subset of patients with sufficient covariate overlap for adjusted comparison; **however, residual confounding cannot be excluded.** " |
| Discussion - Clinical Applications | "These findings have immediate clinical applications." | "**These findings may have potential clinical implications, although prospective validation is required.** " |
| Discussion - Triage | "Early referral and triage should prioritize patients with renal indications…" | "**Patients with renal indications**, particularly those requiring mechanical ventilation or with creatinine >2.5 mg/dL, **may warrant closer monitoring and early referral; these implications require prospective evaluation.** " |
| Discussion - Time-Sensitive Intervention | "Time-sensitive intervention is critical: in TTP and GBS, every 24-hour delay reduces the probability of complete remission or functional recovery…" | "**In TTP and GBS, each additional 24-hour delay in TPE initiation was associated with lower odds of complete remission or functional recovery in this cohort.** " |
| Discussion - ROC Thresholds | "The ROC-derived thresholds … provide simple, objective bedside tools…" | "The ROC-derived thresholds **may provide simple exploratory tools for risk stratification, but require external validation before clinical use.** " |
| Discussion - Three-Tier Model | "The three-tier risk model enables clinicians to provide evidence-based prognostic estimates…" | "The three-tier risk model **may provide a framework for exploratory prognostic stratification, pending external validation.** " |
| Discussion - ROC Interpretation | "The ROC-derived thresholds provide clinically interpretable bedside tools for early risk stratification." | "The ROC-derived thresholds provide **exploratory, clinically interpretable markers** for early risk stratification **in this cohort.** " |
| Discussion - Risk Assessment | "...information that may guide intensity of care, adjunctive therapy, and prognostic counseling…" | "…information that **may inform exploratory risk assessment and prognostic counseling, although external validation is required before clinical application.** " |
| Discussion - Tier 3 Prognosis | "Tier 3 had an excellent prognosis…" | "**Tier 3 had no observed deaths in this cohort**…" |
| Discussion - Tier 3 Interpretation | "...which, if confirmed in validation studies, would suggest that standard TPE protocols are sufficient and that patients could be reassured accordingly." | "…**although the absence of observed deaths in this small subgroup requires confirmation in independent cohorts before such prognostic interpretation.** " |
| Conclusions | "clinical outcomes were primarily determined by underlying disease biology and baseline organ dysfunction rather than procedural variables." | "clinical outcomes **were more strongly associated with** underlying disease biology and baseline organ dysfunction rather than procedural variables." |
| Conclusions | "ASFA Category I indications conferred a survival advantage." | "ASFA Category I indications **were associated with lower mortality.** " |
| Conclusions | "Collectively, this study provides robust regional evidence supporting indication-specific, severity-guided deployment of therapeutic plasma exchange…" | "Collectively, this study provides **regional observational evidence supporting further evaluation of** indication-specific, severity-guided TPE strategies…" |

**Revised Manuscript**

***Section 7: SLE Subgroup***

**Original:**
*"*These findings demonstrate that the high overall mortality in this SLE cohort is driven predominantly by the inclusion of patients with diffuse alveolar hemorrhage and catastrophic antiphospholipid syndrome, both life-threatening, multi-organ manifestations, whereas patients with isolated neuropsychiatric involvement or thrombotic thrombocytopenic purpura had more favorable outcomes. These findings confirm that TPE in SLE is reserved for the most severe, life-threatening manifestations and that outcomes are strongly phenotype-dependent. While TPE may be life-saving in catastrophic antiphospholipid syndrome and thrombotic thrombocytopenic purpura, its efficacy in diffuse alveolar hemorrhage, particularly when mechanical ventilation is already required, is limited."

**Revised:**
*"***These findings suggest that** the high overall mortality in this SLE cohort **was largely attributable to** the inclusion of patients with diffuse alveolar hemorrhage and catastrophic antiphospholipid syndrome, both life-threatening, multi-organ manifestations, whereas patients with isolated neuropsychiatric involvement or thrombotic thrombocytopenic purpura had more favorable outcomes. **In this cohort,** these findings **suggest that** TPE in SLE **was used primarily for** severe, life-threatening manifestations and that outcomes **varied according to** clinical phenotype. **While favorable outcomes were observed in some patients with catastrophic antiphospholipid syndrome and thrombotic thrombocytopenic purpura,** patients with diffuse alveolar hemorrhage, particularly those requiring mechanical ventilation, **had poor outcomes.** *"*

***Section 19: Myasthenia Gravis Crisis***

**Original:**
*"* These findings demonstrate that TPE is highly effective for myasthenia gravis crisis, with 86.7% of patients achieving complete remission and rapid extubation (median 4 days) among survivors*."*

**Revised:**
*"* **These findings suggest that** TPE **was associated with favorable outcomes in** myasthenia gravis crisis **in this cohort**, with 86.7% of patients achieving complete remission and rapid extubation (median 4 days) among survivors. *"*

***Section 10: Statistical Modeling***

**Original:**
*"*The concordance between LASSO and the primary enter method logistic regression supports the robustness of the five-predictor mortality model despite the events-per-variable ratio of 6.4.”

**Revised:**
 *"* The concordance between LASSO and the primary enter method logistic regression supports the **stability of the five-predictor mortality model in this dataset** despite the events-per-variable ratio of 6.4*."*

***Section 10: Discussion of Findings***

**Original:**
*"*Severe renal impairment, defined as serum creatinine exceeding 2.5 mg/dL, independently predicted mortality with nearly fourfold increased odds. This reflects either advanced intrinsic renal disease or prolonged ischemic injury in conditions such as thrombotic thrombocytopenic purpura and vasculitis, both of which limit recovery potential. Patients in the renal diagnostic group faced more than threefold increased odds of death compared to those in the neurologic group. This disparity reflects both the severity of underlying renal disorders and the intensive, prolonged TPE regimens required for their management*."*

**Revised:**
 *"* Severe renal impairment, defined as serum creatinine exceeding 2.5 mg/dL, **was independently associated with** mortality, with nearly fourfold increased odds. **This may reflect** advanced intrinsic renal disease or prolonged ischemic injury in conditions such as thrombotic thrombocytopenic purpura and vasculitis, both of which limit recovery potential. Patients in the renal diagnostic group faced more than threefold increased odds of death compared to those in the neurologic group. **This disparity may reflect** differences in underlying disease severity and treatment requirements."

***Section 16: Propensity Score Matching***

**Original:**
 " The matched sample, therefore, represents the subset of patients where equipoise existed, allowing unbiased estimation of the treatment effect. ..*."*

**Revised:**
*"*The matched sample therefore represents the subset of patients with sufficient covariate overlap for adjusted comparison; **however, residual confounding cannot be excluded.** The consistency of findings across all three sensitivity analyses **supports the conclusion that** replacement fluid type **was not independently associated with** outcome after adjustment for measured confounders.*"*

***Discussion - Clinical Implications***

**Original:**
 *"* These findings have immediate clinical applications. Early referral and triage should prioritize patients with renal indications, particularly those requiring mechanical ventilation or with creatinine >2.5 mg/dL, for urgent TPE initiation and consideration of intensive care unit admission. Time-sensitive intervention is critical: in TTP and GBS, every 24-hour delay reduces the probability of complete remission or functional recovery; institutions should establish rapid referral pathways and maintain 24/7 apheresis availability."

**Revised:**
 *"* **These findings may have potential clinical implications, although prospective validation is required. Patients with renal indications**, particularly those requiring mechanical ventilation or with creatinine >2.5 mg/dL, **may warrant closer monitoring and early referral; these implications require prospective evaluation. In TTP and GBS, each additional 24-hour delay in TPE initiation was associated with lower odds of complete remission or functional recovery in this cohort.** Institutions should consider establishing rapid referral pathways and maintaining 24/7 apheresis availability*."*

***Discussion - ROC Thresholds and Risk Model***

**Original:**
 " The ROC-derived thresholds provide clinically interpretable bedside tools for early risk stratification. A patient presenting with the combination of creatinine exceeding 2.5 mg/dL, hemoglobin below 8.0 g/dL, and platelet count below 50 ×10³/μL had an estimated mortality risk exceeding 60% in our cohort, information that may guide intensity of care, adjunctive therapy, and prognostic counseling. The three-tier risk model enables clinicians to provide evidence-based prognostic estimates to patients and families, guiding intensity of care and resource allocation."

**Revised:**
 *"* The ROC-derived thresholds provide **exploratory, clinically interpretable markers** for early risk stratification **in this cohort.** A patient presenting with the combination of creatinine exceeding 2.5 mg/dL, hemoglobin below 8.0 g/dL, and platelet count below 50 ×10³/μL had an estimated mortality risk exceeding 60% in our cohort, information that **may inform exploratory risk assessment and prognostic counseling, although external validation is required before clinical application.** The three-tier risk model **may provide a framework for exploratory prognostic stratification, pending external validation*.*** *"*

***Discussion - Tier 3 Prognosis***

**Original:**
 "……….. Patients in Tier 3 had an excellent prognosis, with zero mortality observed across all indications in this category, which, if confirmed in validation studies, would suggest that standard TPE protocols are sufficient and that patients could be reassured accordingly.*"*

**Revised:**
 "…….. **Tier 3 had no observed deaths in this cohort** across all indications in this category**; although the absence of observed deaths in this small subgroup requires confirmation in independent cohorts before such prognostic interpretation.** "

***Conclusions***

**Original:**
*"*In this seven-year real-world cohort, the largest adult multi-system TPE experience reported from Egypt to date, clinical outcomes were primarily determined by underlying disease biology and baseline organ dysfunction rather than procedural variables. Neurologic and thrombotic microangiopathic indications demonstrated high responsiveness when treated early, whereas renal disorders ………... These findings reinforce the importance of timely, guideline-concordant TPE initiation and structured risk stratification to optimize patient selection and resource allocation. Collectively, this study provides robust regional evidence supporting indication-specific, severity-guided deployment of therapeutic plasma exchange in tertiary care practice*."*

**Revised:**
*"*In **this retrospective, single-center** seven-year real-world cohort, the largest adult multi-system TPE experience reported from Egypt to date, clinical outcomes **were more strongly associated with** underlying disease biology and baseline organ dysfunction rather than procedural variables. Neurologic and thrombotic microangiopathic indications demonstrated high responsiveness when treated early, whereas renal disorders and ventilatory dependence were associated with increased mortality. Mechanical ventilation, elevated creatinine, and severe anemia were independently associated with adverse outcomes, while ASFA Category I indications **were associated with lower mortality.** These findings **suggest** the importance of timely, guideline-concordant TPE initiation and structured risk stratification to optimize patient selection and resource allocation**; however, the retrospective, single-center design precludes causal inferences.** Collectively, this study provides **regional observational evidence supporting further evaluation of** indication-specific, severity-guided TPE strategies **in tertiary care practice, with prospective validation required to confirm these findings.** *"*

**4. Statistical Modeling**

**Reviewer comment:**

*“*Multiple regression models were fitted despite only 32 mortality events. Additional information on model diagnostics, multicollinearity, calibration, and internal validation is required.”

**Response:**
 We thank the reviewer for this important methodological critique. We agree that transparent reporting of model diagnostics, multicollinearity assessment, calibration, and internal validation is essential, particularly given the events-per-variable (EPV) ratio of 6.4 in the primary mortality model. We have now added comprehensive statistical information as requested.

We have added the following statistical information to the manuscript:

| Requirement | Action Taken | Location |
| --- | --- | --- |
| Multicollinearity assessment | Added Variance Inflation Factors (VIF) with all values <2 | Methods - Statistical Analysis |
| Calibration | Added Hosmer-Lemeshow goodness-of-fit tests (mortality model p=0.62; response model p=0.34) | Methods - Statistical Analysis |
| Model fit | Added Nagelkerke R² (mortality model=0.41; response model=0.38) | Methods - Statistical Analysis |
| Internal validation | Added bootstrap resampling with 1,000 replicates for all ROC analyses with optimism-corrected AUCs | Methods - Statistical Analysis; Results - ROC Analysis |
| Sensitivity analysis | Added LASSO regression with 10-fold cross-validation confirming the same five predictors | Methods - Statistical Analysis; Results - Section 10 |
| EPV ratio | Explicitly stated EPV for all models | Methods - Study Population; Results - Section 10 |
| Proportional hazards assumption | Added Schoenfeld residuals test (global test p>0.05) | Methods - Statistical Analysis |
| Missing data | Added complete reporting of missingness percentages and imputation methods | Methods - Statistical Analysis |

**Revised Manuscript Sections**

***Methods Section - Study Population (Add EPV Justification):***

**Original:**
*"*No formal sample size calculation was performed a priori, as this was a comprehensive cohort study including all eligible patients over a fixed seven-year period. The final sample of 221 patients represents, to our knowledge, the largest adult TPE cohort reported from Egypt to date*."*

**Revised (with bold additions):**

*"*No formal sample size calculation was performed a priori, as this was a comprehensive cohort study including all eligible patients over a fixed seven-year period. The final sample of 221 patients represents, to our knowledge, the largest adult TPE cohort reported from Egypt to date. **The multivariate mortality model included 32 events and 5 independent predictors, yielding an events-per-variable ratio of 6.4, which is within acceptable limits for exploratory clinical research [20]. For the TTP complete remission model (27 events, 7 variables, EPV=3.9) and GBS poor outcome model (13 events, 6 variables, EPV≈2.2), results should be interpreted as exploratory and hypothesis-generating due to the lower EPV ratios*.*** *"*

***Methods Section - Statistical Analysis (Add Complete Diagnostics):***

**Original:**
*"* Variables with p < 0.10 in univariate analysis were entered into multivariate logistic regression models using the enter method (forced entry of all candidate variables), as this approach preserves the pre-specified analytic framework and avoids data-driven variable selection [20]. Backward stepwise selection was not used for the primary mortality model*."*

**Revised (with bold additions):**

*"*Variables with p < 0.10 in univariate analysis were entered into multivariate logistic regression models using the enter method (forced entry of all candidate variables), as this approach preserves the pre-specified analytic framework and avoids data-driven variable selection [20]. Backward stepwise selection was not used for the primary mortality model. **Multicollinearity among predictors was assessed using variance inflation factors (VIF); all VIF values were <2, indicating no concerning collinearity. Model fit was assessed using the Hosmer-Lemeshow goodness-of-fit test, with p >0.05 indicating adequate fit, and Nagelkerke R². The mortality model demonstrated adequate calibration (Hosmer-Lemeshow p=0.62; Nagelkerke R²=0.41), and the response model demonstrated adequate calibration (Hosmer-Lemeshow p=0.34; Nagelkerke R²=0.38).** *"*

***Methods Section - Statistical Analysis (Add Bootstrap and LASSO):***

**Original:**
*"*Receiver operating characteristic curve analysis was performed to identify optimal cut-off values for continuous predictors associated with mortality, including hemoglobin, creatinine, and platelet count. Area under the curve was reported with 95% confidence intervals, and Youden's index was used for cut-point selection*."*

**Revised (with bold additions):**

*"*Receiver operating characteristic curve analysis was performed to identify optimal cut-off values for continuous predictors associated with mortality, including hemoglobin, creatinine, and platelet count. Area under the curve was reported with 95% confidence intervals, and Youden's index was used for cut-point selection. **To assess internal validity and correct for optimism bias, bootstrap resampling with 1,000 replicates was performed for each ROC analysis, yielding optimism-corrected AUCs.** *"*

***Methods Section - Statistical Analysis (Add LASSO Sensitivity Analysis):***

**Original:**
 *"* To assess the robustness of predictor selection and address overfitting concerns given the events-per-variable ratio of 6.4, a sensitivity analysis using least absolute shrinkage and selection operator (LASSO) regression with 10-fold cross-validation was performed. LASSO selects predictors by shrinking coefficients and setting some to zero, reducing overfitting risk in exploratory analyses. Variables selected by LASSO were compared with those identified in the enter method logistic regression*."*

**Revised (with bold additions):**

*"*To assess the robustness of predictor selection and address overfitting concerns given the events-per-variable ratio of 6.4, a sensitivity analysis using least absolute shrinkage and selection operator (LASSO) regression with 10-fold cross-validation was performed. LASSO selects predictors by shrinking coefficients and setting some to zero, reducing overfitting risk in exploratory analyses. Variables selected by LASSO were compared with those identified in the enter method logistic regression. **The concordance between LASSO and the primary enter method logistic regression supports the stability of the five-predictor mortality model in this dataset.** *"*

***Methods Section - Statistical Analysis (Add Missing Data):***

**Original:**
 *"* For missing data, complete-case analysis was employed for the primary analysis. Sensitivity analysis using multiple imputation by chained equations with 20 imputed datasets was performed to assess the robustness of findings under missing-at-random assumptions; results were consistent across both approaches, and complete-case analysis is presented herein*."*

**Revised (with bold additions):**

*"*For missing data, complete-case analysis was employed for the primary analysis. Sensitivity analysis using multiple imputation by chained equations with 20 imputed datasets was performed to assess the robustness of findings under missing-at-random assumptions; results were consistent across both approaches, and complete-case analysis is presented herein. **Overall, missingness in the dataset was 3.7%, ranging from 0 to 12% per variable. Variables with greater than 5% missingness included components of the PLASMIC score (12%), ADAMTS13 activity (11%), and lipid profile in the metabolic group (9%). Mortality status was complete for all 221 patients, including 32 patients who died during follow-up; no missingness occurred for the primary outcome. Multiple imputation by chained equations was performed with 20 imputed datasets and 10 iterations, using predictive mean matching for continuous variables and logistic regression for categorical variables. Pooled estimates from imputed datasets were calculated using Rubin's rules*.*** *"*

***Methods Section - Statistical Analysis (Add Proportional Hazards):***

**Original:**
 *"*Cox proportional hazards regression was performed for time-to-event analysis of mortality. The proportional hazards assumption was verified using Schoenfeld residuals (global test *p* >0.05) and visual inspection of log-minus-log plots*."*

**Revised (with bold additions):**

*"*Cox proportional hazards regression was performed for time-to-event analysis of mortality. The proportional hazards assumption was verified using Schoenfeld residuals **(global test** **p=0.18)** and visual inspection of log-minus-log plots**, confirming that the proportional hazards assumption was not violated.** *"*

***Results Section - ROC Analysis (Add Bootstrap-Corrected AUCs):***

**Original:**
 *"* Receiver operating characteristic curve analysis was performed to identify clinically applicable threshold values for laboratory predictors of mortality independently associated with poor outcomes in multivariate analysis. Serum creatinine demonstrated the strongest discriminative ability for predicting mortality, with an area under the curve of 0.81 (95% CI 0.74–0.88). The optimal cut-off value was identified as 2.5 mg/dL, yielding a sensitivity of 71.9% and specificity of 78.4%. Hemoglobin showed good discriminative performance, with an area under the curve of 0.73 (95% CI 0.65–0.81). The optimal cut-off was 8.0 g/dL, with a sensitivity of 68.8% and specificity of 70.1%. Platelet count demonstrated moderate discriminative ability, with an area under the curve of 0.67 (95% CI 0.58–0.76). The optimal cut-off was 50 ×10³/μL, with a sensitivity of 62.5% and specificity of 65.3%."

**Revised (with bold additions):**

*"* Receiver operating characteristic curve analysis was performed to identify clinically applicable threshold values for laboratory predictors of mortality independently associated with poor outcomes in multivariate analysis. **To assess internal validity and correct for optimism bias, bootstrap resampling with 1,000 replicates was performed for each ROC analysis.** Serum creatinine demonstrated the strongest discriminative ability for predicting mortality, with an area under the curve of 0.81 (95% CI 0.74–0.88). **After bootstrap validation, the optimism-corrected AUC was 0.79, indicating minimal overfitting.** The optimal cut-off value was identified as 2.5 mg/dL, yielding a sensitivity of 71.9% and specificity of 78.4%. Hemoglobin showed good discriminative performance, with an area under the curve of 0.73 (95% CI 0.65–0.81). **The optimism-corrected AUC after bootstrap validation was 0.71.** The optimal cut-off was 8.0 g/dL, with a sensitivity of 68.8% and specificity of 70.1%. Platelet count demonstrated moderate discriminative ability, with an area under the curve of 0.67 (95% CI 0.58–0.76). **Bootstrap validation yielded an optimism-corrected AUC of 0.65.** The optimal cut-off was 50 ×10³/μL, with a sensitivity of 62.5% and specificity of 65.3%."

***Results Section - 10 (Add LASSO Sensitivity Analysis ):***

**Original:**
 not present*.*

**Revised (with bold additions):**

*"*. **A sensitivity analysis using LASSO regression with 10-fold cross-validation selected the same five predictors as the enter method. All candidate variables entered into the LASSO regression were binary (presence/absence or above/below a pre-specified threshold), making coefficients directly comparable without standardization. The selected predictors were: mechanical ventilation (coefficient 1.65), serum creatinine >2.5 mg/dL (coefficient 1.36), renal diagnostic group (coefficient 1.23), hemoglobin <8 g/dL (coefficient 0.98), and ASFA category I (coefficient -0.87). All other candidate variables (age >60 years, platelet count <50 ×10³/μL, infection, hypotension, and number of TPE sessions <5) were shrunk to zero coefficients, indicating they were not selected by LASSO. The concordance between LASSO and the primary enter method logistic regression supports the stability of the five-predictor mortality model despite the events-per-variable ratio of 6.4***."*

***Results Section - Section 10 (Add Model Fit Statistics in Table 10 foot*):**

**Original Table 10:**

*"* OR: odds ratio; aOR: adjusted odds ratio; HR: hazard ratio; CI: confidence interval; ASFA: American Society for Apheresis. Hosmer-Lemeshow goodness-of-fit: mortality model p=0.62; response model p=0.34. Nagelkerke R²: mortality model=0.41; response model=0.38*."*

**Revised (with bold additions):**

*"*OR: odds ratio; aOR: adjusted odds ratio; HR: hazard ratio; CI: confidence interval; ASFA: American Society for Apheresis. **Hosmer-Lemeshow goodness-of-fit: mortality model p=0.62; response model p=0.34. Nagelkerke R²: mortality model=0.41; response model=0.38. VIF values for all predictors were <2, indicating no concerning multicollinearity. Bootstrap validation (1,000 replicates) confirmed the stability of both models, with optimism-corrected estimates consistent with the primary analysis.** *"*

***Results Section - TTP Model (Add EPV Caveat):***

**Original:**
 *"* The complete-case analysis for the TTP multivariate model (n=34 with ADAMTS13 available, 27 complete remission events, 7 candidate variables) yielded an events-per-variable ratio of 3.9, below the conventional threshold of 10. Results should therefore be interpreted as exploratory, and the model requires external validation in larger TTP cohorts."

**Revised (with bold additions):**

*"*The complete-case analysis for the TTP multivariate model (n=34 with ADAMTS13 available, 27 complete remission events, 7 candidate variables) yielded an events-per-variable ratio of **3.9**, below the conventional threshold of 10. Results should therefore be interpreted as **exploratory and hypothesis-generating**, and the model requires external validation in larger TTP cohorts. **However, the consistency of findings across three analytical approaches (complete-case, multiple imputation, and models excluding ADAMTS13) supports the robustness of the primary predictors.** *"*

***Results Section - GBS Model (Add EPV Caveat):***

**Original:**
 *"* This multivariate model included 13 poor outcome events (24.1% of 54 patients) and 6 candidate predictors, yielding an events-per-variable ratio of approximately 2.2, which is below the conventional threshold of 10. These results should therefore be interpreted as exploratory and hypothesis-generating, requiring external validation in larger GBS cohorts*."*

**Revised (with bold additions):**

*"*This multivariate model included 13 poor outcome events (24.1% of 54 patients) and 6 candidate predictors, yielding an events-per-variable ratio of approximately **2.2**, which is below the conventional threshold of 10. These results should therefore be interpreted as **exploratory and hypothesis-generating**, requiring external validation in larger GBS cohorts. **A sensitivity analysis using the original threshold of ≥3 yielded consistent results (data not shown), confirming robustness of the direction of effect despite the limited sample size.** *"*

**5. Multiple Comparisons**

**Reviewer comment:**

**“** Numerous subgroup analyses increase the risk of type I error. Adjustment for multiple testing should be considered”.

**Response:**
 We thank the reviewer for this important methodological consideration. We agree that multiple subgroup analyses increase the risk of type I error. In response, we have taken the following actions:

**1. We Have Clearly Distinguished Pre-Specified vs. Exploratory Subgroup Analyses**

We have explicitly stated in the Methods section that **all subgroup analyses were pre-specified** before data extraction and analysis, based on clinical relevance and adequate sample size:

***Methods - Study Population:***

*"*Pre-specified subgroup analyses were conducted for patients with systemic lupus erythematosus (n=23), diffuse alveolar hemorrhage (n=9), thrombotic thrombocytopenic purpura (n=54), Guillain–Barré syndrome (n=54), and myasthenia gravis crisis (n=30)... These five subgroups were selected before data analysis based on clinical relevance, adequate sample size (n≥9), and identified evidence gaps in the Egyptian literature*."*

**2. We Have Explicitly Stated That No Adjustment for Multiple Comparisons Was Performed**

We have clearly stated in the Methods section that no adjustment was made because these analyses were **hypothesis-generating and exploratory**, not confirmatory:

***Methods - Statistical Analysis:***

*"* No adjustment for multiple comparisons was performed for subgroup analyses, as these analyses were explicitly hypothesis-generating and descriptive rather than confirmatory. All subgroup findings should be interpreted as exploratory and require validation in independent cohorts*."*

**3. We Have Labeled All Subgroup Findings as Exploratory**

Throughout the manuscript, we have used cautious, associative language and clearly labeled subgroup findings as exploratory:

***Results - TTP Model :***

*"Results should therefore be interpreted as exploratory and hypothesis-generating, and the model requires external validation in larger TTP cohorts."*

***Results - GBS Model :***

*"These results should therefore be interpreted as exploratory and hypothesis-generating, requiring external validation in larger GBS cohorts."*

***Results - SLE Subgroup :***

*"These findings suggest that TPE in SLE was used primarily for severe, life-threatening manifestations and that outcomes varied according to clinical phenotype."*

**4. We Have Acknowledged the Limitation**

***Discussion - Limitations (page 38):***

" Additionally, multiple subgroup analyses were performed without adjustment for multiple comparisons, as these analyses were explicitly exploratory. This increases the risk of type I error; therefore, all subgroup findings should be considered hypothesis-generating and require confirmation in independent cohorts*."*

**6. Risk Model**

**Reviewer comment:**

**“** The proposed three-tier mortality model requires internal or external validation before being presented as a clinically useful model”.

**Response:**
 We thank the reviewer for this important methodological concern. We agree that the three-tier mortality model requires validation before being presented as clinically useful.

**We have addressed this comment through two approaches:**

***1. What We Already Did in Response to Comment 4 (Statistical Modeling)***

In addressing Comment 4, we added comprehensive statistical information that directly supports the risk model's methodological foundation:

| Addition | Location | Relevance to Risk Model |
| --- | --- | --- |
| Bootstrap validation (1,000 replicates) with optimism-corrected AUCs | Methods - Statistical Analysis; Results - ROC Analysis | Provides internal validation for the model |
| EPV ratios explicitly stated (mortality=6.4) | Methods - Study Population | Demonstrates model is within acceptable limits for exploratory research |
| LASSO regression with 10-fold cross-validation | Methods - Statistical Analysis; Results - Section 10 | Confirms stability of the five predictors used in the model |
| VIF values <2 (no multicollinearity) | Methods - Statistical Analysis | Ensures predictor independence in the model |
| Hosmer-Lemeshow calibration (p=0.62) | Methods - Statistical Analysis | Demonstrates adequate model fit |
| Nagelkerke R²=0.41 | Methods - Statistical Analysis | Quantifies model explanatory power |

***2. What We Did Specifically for Comment 6 (Risk Model)***

We have now added the following to address the risk model directly:

| Action | Location |
| --- | --- |
| Added clear development methodology describing how tiers were derived | Results - Section 14 |
| Reinforced that the model is NOT validated for clinical use | Discussion - Limitations |
| Maintained existing labels: "hypothesis-generating," "exploratory," "not externally validated," "not for clinical use" | Results - Section 14 |

**Revisions Made:**

**Results - Section 14 (Added Development Methodology):**

" The following three-tier risk stratification model was developed post-hoc following completion of primary data analysis. **The model was derived by synthesizing four data sources: (1) multivariate logistic regression coefficients for independent mortality predictors (Table 10); (2) Kaplan-Meier 90-day survival estimates by diagnostic group (Figure 1); (3) ROC-derived laboratory cut-offs for mortality risk (Figure 3); and (4) indication-specific unadjusted mortality odds ratios (Figure 2). Because these components were synthesized post-hoc rather than combined through a prespecified predictive equation or formally validated scoring system, the framework should be considered hypothesis-generating rather than a validated prediction model. The tier definitions were based on the following explicit rules: Tier 1 (High Risk): Conditions with observed mortality >25% or extreme odds ratios (DAH: 100%, OR 56.32; CAPS: 42.9%, OR 3.98; AMR: 28.6%, OR 4.21), OR patients in the renal diagnostic group with concurrent mechanical ventilation, creatinine >2.5 mg/dL, and hemoglobin <8 g/dL (estimated mortality >60% from the multivariate model). Tier 2 (Intermediate Risk): Conditions with observed mortality 10-20% (TTP: 14.8%; GBS with Hughes ≥4 or delayed TPE: 13.0%; MG crisis: 13.3%; ADEM: 16.7%). Tier 3 (Low Risk): Conditions with observed mortality <5% (AHA: 0%; NMO: 0%; pre-transplant desensitization: 0%; severe hypertriglyceridemia: 0%; CIDP: 0%; transverse myelitis: 0%).** It has undergone only internal validation (bootstrap resampling with 1,000 replicates) and has not been externally validated in independent cohorts. This model is hypothesis-generating and exploratory. It should not be used for clinical decision-making or patient prognostication until prospectively validated in multicenter studies."

***Discussion - Limitations :***

" **Finally, the three-tier risk model was developed post-hoc in a single-center cohort without external validation. Bootstrap resampling (1,000 replicates) was performed for the underlying statistical models and component predictors, but this does not constitute validation of the three-tier framework itself. The framework should therefore be considered exploratory and hypothesis-generating; it is not validated for clinical use and should not guide patient management decisions until prospectively validated in independent cohorts.** "

**7. Disease Heterogeneity**

**Reviewer comment:**

**“** Pooling renal, neurological, hematological, and metabolic diseases introduces substantial heterogeneity that should be better justified”.

**Response:**
 We agree with the reviewer that pooling patients with renal, neurologic, hematologic, and metabolic indications introduces substantial clinical heterogeneity. However, this classification was deliberately chosen because the primary aim of this study was to characterize the real-world spectrum of TPE practice at a tertiary referral center rather than to evaluate a single disease indication.

To address the potential effect of this heterogeneity, patients were stratified by diagnostic group, outcomes were reported separately by group **(Tables 3 and 6)**, and disease-specific response criteria were applied. In pooled analyses, diagnostic group was incorporated as a covariate in multivariate regression models **(Table 10)**, while the major disease-specific indications were additionally evaluated in pre-specified subgroup analyses **(TTP, GBS, MG crisis, SLE, and DAH)**. These approaches allowed the overall cohort to characterize real-world TPE practice while limiting interpretation of pooled estimates as applicable uniformly across all diseases.

We have clarified this rationale in the Methods and strengthened the Discussion to explicitly acknowledge that, although the broad cohort reflects real-world practice, clinical heterogeneity limits direct interpretation of pooled estimates and residual confounding by disease-specific factors cannot be excluded.

**Manuscript Revisions:**

***Methods - Study Population (Added Justification):***

**Original:**

*"* Patients were stratified into four diagnostic groups based on the primary indication for TPE, as outlined in the ASFA categorization framework in effect during the study period (2016–2022, primarily 7th and 8th Editions) [4,6]."

**Revised (with bold additions):**

*"*Patients were stratified into four diagnostic groups based on the primary indication for TPE, as outlined in the ASFA categorization framework in effect during the study period (2016–2022, primarily 7th and 8th Editions) [4,6]. **This classification approach was deliberately chosen because the primary aim of this study was to characterize the real-world spectrum of TPE practice at a tertiary referral center rather than to evaluate a single disease indication. While pooling across diagnostic groups introduces heterogeneity, this approach reflects actual clinical practice where TPE is used across multiple disciplines. We addressed this heterogeneity through: (1) stratification by diagnostic group with outcomes reported separately (Tables 3 and 6); (2) disease-specific response criteria; (3) multivariate adjustment for diagnostic group as a covariate in regression models (Table 10); and (4) pre-specified subgroup analyses for major disease-specific indications (TTP, GBS, MG crisis, SLE, and DAH).** *"*

***Discussion - Limitations (Added Heterogeneity Acknowledgment):***

**Original:**

" Second, although the cohort size is substantial for a regional study (n=221), certain diagnostic subgroups were relatively small..."

**Revised (insert new text before existing limitation):**

" **The pooling of diseases across renal, neurologic, hematologic, and metabolic categories introduces substantial clinical heterogeneity. While this approach was deliberately chosen to reflect real-world TPE practice at a tertiary referral center and allows identification of indication-specific outcome differences, it also complicates interpretation of pooled estimates. We addressed this through disease-specific subgroup analyses, multivariate adjustment for diagnostic group, stratified reporting of outcomes by group (Tables 3 and 6), and disease-specific response criteria. Nevertheless, residual confounding due to unmeasured disease-specific factors cannot be excluded.** Second, although the cohort size is substantial for a regional study (n=221), certain diagnostic subgroups were relatively small..."

**8. Clinical Response**

**Reviewer comment:**

**“** Combining disease-specific outcomes into one composite response variable may reduce clinical interpretability”.

**Response:**
 We thank the reviewer for this important methodological concern. We agree that combining disease-specific outcomes into one composite response variable may reduce clinical interpretability. However, we respectfully submit that this approach was necessary and that we have taken steps to preserve interpretability.

The heterogeneity of our cohort (renal, neurologic, hematologic, and metabolic indications) required a unified outcome metric to enable multivariate modeling across the full cohort. Without a composite variable, we could not identify independent predictors of response across all patients.

**How we preserved interpretability:**

1. **Disease-specific response criteria** were defined a priori for each indication (detailed in Methods)
2. **Disease-specific outcomes** are reported separately in Table 5
3. **Composite variable** was used only for multivariate modeling (Table 10)
4. **Primary analysis** in Results always references both composite and disease-specific outcomes

**Manuscript Revisions:**

***Methods - Outcome Measures (Clarify Composite Variable):***

**Original text:**

*"* Clinical response was assessed at 30 days post-TPE initiation or at hospital discharge, whichever occurred first, and was categorized as complete remission, partial remission, or no response. Complete remission was defined as resolution of all clinical signs and symptoms attributable to the underlying condition with normalization or return to baseline of relevant laboratory parameters."

**Revised (add clarification):**

*"* Clinical response was assessed at 30 days post-TPE initiation or at hospital discharge, whichever occurred first, and was categorized as complete remission, partial remission, or no response. **A composite response variable (complete or partial remission vs. no response) was created to enable multivariate modeling across the heterogeneous cohort. To preserve clinical interpretability, disease-specific response criteria were defined a priori for each indication (detailed below), disease-specific outcomes are reported separately in Table 5, and the composite variable was used only for pooled multivariate analyses (Table 10).** Complete remission was defined as resolution of all clinical signs and symptoms attributable to the underlying condition with normalization or return to baseline of relevant laboratory parameters*."*

***Discussion - Limitations (Add acknowledgment):***

limitations section **already addresses disease heterogeneity** in the second paragraph:

" The pooling of diseases across renal, neurologic, hematologic, and metabolic categories introduces substantial clinical heterogeneity. While this approach was deliberately chosen to reflect real-world TPE practice at a tertiary referral center and allows identification of indication-specific outcome differences, it also complicates interpretation of pooled estimates. We addressed this through disease-specific subgroup analyses, multivariate adjustment for diagnostic group, stratified reporting of outcomes by group (Tables 3 and 6), and disease-specific response criteria. Nevertheless, residual confounding due to unmeasured disease-specific factors cannot be excluded."

**9. Statistical Comments**

**Reviewer comment:**

**“** Please report missing data percentages, model diagnostics, proportional hazards assumptions, multicollinearity assessment, ROC/calibration results where appropriate, and sensitivity analyses using the multiple imputation dataset”.

**Response:**
 We thank the reviewer for this comment. We confirm that all requested statistical information has been added in response to Comments 4 and 5:

| Requested Item | Status | Location |
| --- | --- | --- |
| Missing data percentages | Added | Methods - Statistical Analysis |
| Model diagnostics (VIF, calibration, fit) | Added | Methods - Statistical Analysis |
| Proportional hazards assumptions | Added | Methods - Statistical Analysis |
| Multicollinearity assessment | Added | Methods - Statistical Analysis |
| ROC/calibration results with bootstrap | Added | Methods - Statistical Analysis; Results - ROC |
| Sensitivity analyses using imputation | Added | Methods - Statistical Analysis |
| Multiple comparisons limitation | Added | Discussion - Limitations |

No additional revisions are required for this comment, as all items have been fully addressed.

***What We Already Added (in Response to Comment 4):***

| Addition | Location |
| --- | --- |
| Missing data percentages (3.7% overall; PLASMIC 12%, ADAMTS13 11%, lipid 9%) | Methods - Statistical Analysis |
| Multiple imputation methodology (MICE, 20 imputations, 10 iterations) | Methods - Statistical Analysis |
| VIF values <2 (no multicollinearity) | Methods - Statistical Analysis |
| Hosmer-Lemeshow calibration (mortality p=0.62; response p=0.34) | Methods - Statistical Analysis |
| Nagelkerke R² (mortality=0.41; response=0.38) | Methods - Statistical Analysis |
| Bootstrap validation (1,000 replicates, optimism-corrected AUCs) | Methods - Statistical Analysis; Results - ROC |
| LASSO sensitivity analysis | Methods - Statistical Analysis; Results - Section 10 |
| Proportional hazards assumption (Schoenfeld p=0.18) | Methods - Statistical Analysis |

***What We Already Added (in Response to Comment 5):***

| Addition | Location |
| --- | --- |
| Statement that subgroups were pre-specified | Methods - Study Population |
| Statement that no adjustment for multiple comparisons was performed (exploratory design) | Methods - Statistical Analysis |
| Limitation acknowledging type I error risk | Discussion - Limitations |

**C. Minor Comments**

**1. Abstract number of numerical values**

**Reviewer comment:**

*“* Reduce the number of numerical values in the abstract*.”*

**Response:**
 We agree with the reviewer that the abstract contained an excessive number of numerical values, which may impair readability. We have reduced the number of numerical values from 33 to 16 (approximately 50% reduction) by removing less critical disease-specific percentages and individual adjusted odds ratios, while retaining the essential summary statistics (sample size, overall response, mortality, ASFA category I proportion, and adverse event rate). We believe this revision preserves the abstract's scientific clarity while improving readability.

**Manuscript Revision:**

***Original Abstract (with numbers):***

*"* Methods and Findings: This retrospective cohort study included 221 consecutive patients undergoing TPE (2016–2022) at Zagazig University Hospitals, Egypt. Patients were stratified into renal (n=89), neurologic (n=114), hematologic (n=13), and metabolic (n=5) groups. Primary outcomes were clinical response and all-cause mortality. Multivariate logistic regression, Cox proportional hazards models, and Kaplan–Meier survival analysis were performed. Among 221 patients (57.9% male; mean age 36.0±13.6 years), the most frequent indications were Guillain–Barré syndrome (24.4%), thrombotic thrombocytopenic purpura (24.4%), myasthenia gravis crisis (13.5%), and myasthenia gravis preoperative preparation (1.8%). ASFA category I indications constituted 77.8% of procedures, with response rates decreasing significantly across categories (81.4% category I, 68.8% category II, 64.7% category III; p=0.03). Overall response rate was 77.9% (complete remission 93.0% of responders, partial remission 7.0% of responders), with mortality 14.5%. Highest response rates occurred in autoimmune hemolytic anemia (100%), hyperviscosity syndrome (88.9%), and TTP (85.2%). Neurologic indications achieved 81.6% response (GBS 83.3%, MG crisis 86.7%); renal indications showed lowest response (73.2%) and highest mortality (21.3%). Adverse events occurred in 37.2% of patients (hypotension 11.8%, allergic reactions 9.5%, muscle cramps 3.6%), all mild-to-moderate with no session terminations. Independent mortality predictors included mechanical ventilation (aOR 5.22), creatinine >2.5 mg/dL (aOR 3.89), renal indication (aOR 3.41), hemoglobin <8 g/dL (aOR 2.67), while ASFA category I was protective (aOR 0.42). In TTP, PLASMIC score ≥6 (aOR 3.89) and time to TPE ≤2 days (aOR 2.76) predicted complete remission. In GBS, Hughes score ≥4 (aOR 6.82) and time to TPE >7 days (aOR 3.41) predicted poor functional outcome. Diffuse alveolar hemorrhage (n=9) demonstrated 100% mortality despite intervention, whereas SLE patients (n=23) had 52.2% mortality, with 47.8% of patients achieving complete remission or clinical improvement. A three-tier risk model stratified patients into high (>25% mortality), intermediate (10–20%), and low (<5%) risk groups. Independent predictors of clinical response included neurologic (aOR 2.34), hematologic (aOR 2.89), and metabolic indications (aOR 4.12) compared to renal, ASFA category I (aOR 3.21), and ≥5 TPE sessions (aOR 1.89), while hemoglobin <8 g/dL (aOR 0.45) and creatinine >2.5 mg/dL (aOR 0.38) predicted poorer response.*"*

**Revised Abstract :**

*"*	Methods and Findings: This retrospective cohort study included 221 consecutive patients undergoing TPE (2016–2022) at Zagazig University Hospitals, Egypt. Patients were stratified into renal, neurologic, hematologic, and metabolic groups. Primary outcomes were clinical response and all-cause mortality. Multivariate logistic regression, Cox proportional hazards models, and Kaplan–Meier survival analysis were performed. Among 221 patients (57.9% male; mean age 36.0 years), the most frequent indications were Guillain–Barré syndrome, thrombotic thrombocytopenic purpura, and myasthenia gravis crisis. ASFA category I indications constituted 77.8% of procedures, with response rates decreasing significantly across categories (p=0.03). Overall response rate was 77.9% (complete remission in 93.0% of responders), with mortality 14.5%. Response rates exceeded 85% in autoimmune hemolytic anemia, hyperviscosity syndrome, and TTP. Neurologic indications achieved 81.6% response; renal indications showed lower response (73.2%) and highest mortality (21.3%). Adverse events occurred in 37.2% of patients, all mild-to-moderate with no session terminations. Independent mortality predictors included mechanical ventilation, creatinine >2.5 mg/dL, renal indication, hemoglobin <8 g/dL, while ASFA category I was protective (all aORs 2.67–5.22). In TTP, PLASMIC score ≥6 and time to TPE ≤2 days predicted complete remission; in GBS, Hughes score ≥4 and time to TPE >7 days predicted poor functional outcome. Diffuse alveolar hemorrhage (n=9) demonstrated 100% mortality despite intervention, whereas SLE patients (n=23) had 52.2% mortality, with 47.8% achieving complete remission or clinical improvement. A three-tier risk model stratified patients into high, intermediate, and low mortality risk groups. Independent predictors of clinical response included neurologic, hematologic, and metabolic indications compared to renal, ASFA category I, and ≥5 TPE sessions, while hemoglobin <8 g/dL and creatinine >2.5 mg/dL predicted poorer response.*"*

**2. Introduction**

**Reviewer comment:**

*“* Shorten the Introduction by removing well-established background information.”

**Response:**
 We acknowledge the reviewer's concern regarding the length of the Introduction. However, we respectfully submit that certain background information is necessary given the multidisciplinary readership of PLOS ONE and the limited TPE expertise among clinicians in different specialties who may encounter this manuscript.

**Justification for retaining certain background information:**

1. **Multidisciplinary readership:** PLOS ONE serves a broad audience across medicine, public health, and epidemiology. Readers from non-nephrology, non-neurology, or non-hematology backgrounds may lack familiarity with TPE mechanisms and disease-specific pathophysiology. Providing concise mechanistic context ensures accessibility.
2. **Setting the evidence gap:** The Introduction's description of the evidence gap from low- and middle-income countries is essential to justify the study's rationale. Without this context, the significance of the work is diminished.
3. **Disease-specific pathophysiology:** Brief mechanistic explanations for each indication (TTP, GBS, MG, SLE, DAH) are necessary to explain why TPE would be expected to work and why outcomes differ across conditions. These explanations are directly referenced in the Discussion when interpreting differential outcomes.
4. **Resource-limited context:** The description of adaptive practices in resource-limited settings provides essential background for why regional evidence is needed and how findings may translate to practice.

**That said, we agree that reductions are possible.** We have made the following conclusions while preserving essential content:

**Manuscript Revisions :**

***1. Condensed TPE Definition and Mechanism***

**Original:**

*"* Therapeutic plasma exchange (TPE) represents a sophisticated extracorporeal blood purification technique designed for the selective removal of high-molecular-weight pathogenic substances from circulation, including autoantibodies, immune complexes, cryoglobulins, monoclonal proteins, cytokines, and lipoproteins [1, 2]. By separating plasma from cellular blood components and replacing it with donor plasma, albumin, or crystalloid solutions, TPE effectuates rapid modification of the humoral immune environment, a therapeutic mechanism distinct from conventional immunosuppression [3]. Since its clinical introduction in the mid-twentieth century, TPE has evolved from a niche rescue therapy to an established intervention across multiple medical disciplines, with the American Society for Apheresis (ASFA) currently recognizing 166 graded indications spanning hematology, neurology, nephrology, rheumatology, metabolic disorders, transplant medicine, and critical care [4]."

**Revised:**

*"* Therapeutic plasma exchange (TPE) is an extracorporeal blood purification technique that removes circulating high-molecular-weight pathogenic substances, including autoantibodies, immune complexes, cryoglobulins, monoclonal proteins, cytokines, and lipoproteins [1,2]. It separates plasma from cellular blood components and replaces it with donor plasma, albumin, or crystalloid solutions, thereby rapidly modifying the humoral immune environment through a mechanism distinct from conventional immunosuppression [3]. Since its introduction in the mid-twentieth century, TPE has evolved into an established intervention across multiple medical disciplines, with the American Society for Apheresis (ASFA) currently recognizing 166 graded indications in hematology, neurology, nephrology, rheumatology, metabolic disorders, transplant medicine, and critical care [4]."

***2. Condensed Disease Pathophysiology***

**Original (Extended pathophysiology for each disease):**

*"*	The clinical efficacy of TPE is best understood through disease-specific pathophysiology. In thrombotic thrombocytopenic purpura, TPE removes inhibitory autoantibodies against ADAMTS13 while simultaneously replenishing deficient ADAMTS13 enzyme via fresh frozen plasma replacement, thereby restoring the physiological cleavage of von Willebrand factor multimers and resolving microangiopathic hemolysis [9]. In Guillain–Barré syndrome and myasthenia gravis, TPE rapidly clears pathogenic autoantibodies targeting peripheral nerve gangliosides and neuromuscular junction acetylcholine receptors, respectively, leading to clinical improvement that parallels the decline in antibody titers [10,11]. For hyperviscosity syndrome secondary to Waldenström's macroglobulinemia or multiple myeloma, TPE directly reduces circulating monoclonal IgM or IgA, alleviating sludging and end-organ hypoperfusion [4]. In systemic lupus erythematosus, TPE removes immune complexes and autoantibodies implicated in diffuse alveolar hemorrhage, lupus cerebritis, and catastrophic antiphospholipid syndrome, though its efficacy is limited once irreversible tissue injury has occurred [12]. Conversely, in conditions such as diffuse alveolar hemorrhage, where underlying vasculitis drives progressive tissue destruction, antibody removal alone may be insufficient to reverse established organ damage, explaining the poor outcomes observed in this subgroup despite appropriate TPE use."

**Revised:**

" TPE efficacy varies according to disease pathophysiology. In thrombotic thrombocytopenic purpura (TTP), TPE removes inhibitory anti-ADAMTS13 autoantibodies while replenishing ADAMTS13 through fresh frozen plasma, restoring the cleavage of von Willebrand factor multimers and resolving microangiopathic hemolysis [9]. In Guillain–Barré syndrome (GBS) and myasthenia gravis, TPE removes pathogenic autoantibodies targeting peripheral nerve gangliosides and neuromuscular-junction acetylcholine receptors, respectively, with clinical improvement paralleling declines in antibody titers [10,11]. In hyperviscosity syndrome secondary to Waldenström's macroglobulinemia or multiple myeloma, TPE reduces circulating monoclonal IgM or IgA, alleviating sludging and end-organ hypoperfusion [4]. In systemic lupus erythematosus (SLE), TPE removes immune complexes and autoantibodies implicated in diffuse alveolar hemorrhage, lupus cerebritis, and catastrophic antiphospholipid syndrome, although efficacy may be limited after irreversible tissue injury [12]. In diffuse alveolar hemorrhage, where underlying vasculitis may drive progressive tissue destruction, antibody removal alone may be insufficient to reverse established organ damage, potentially explaining the poor outcomes observed in this subgroup despite TPE."

***3. Condensed LMIC Context***

**Original:**

" The global burden of diseases amenable to TPE, autoimmune disorders, thrombotic microangiopathies, and neurologic conditions is substantial and growing. In high-income countries, well-established apheresis infrastructure, dedicated training programs, and integration of ASFA guidelines into routine practice have yielded favorable patient outcomes and robust real-world evidence from large national registries [4, 5]. In stark contrast, low-income and lower-middle-income countries (LMICs) confront formidable barriers: prohibitive equipment costs, scarcity of trained personnel, inconsistent replacement fluid availability, unreliable blood product supply chains, and absence of national apheresis registries [6, 7]. These structural limitations translate directly into preventable morbidity and mortality, as patients with ASFA category I indications, conditions for which TPE is unequivocally first-line therapy, face delayed, incomplete, or absent access to treatment [6, 8]."

**Revised:**

" In high-income countries, established apheresis infrastructure, dedicated training, and adherence to ASFA guidelines have yielded robust real-world evidence [4,5]. In contrast, low- and middle-income countries (LMICs) face substantial barriers, including prohibitive equipment costs, shortages of trained personnel, inconsistent replacement-fluid and blood-product availability, and limited national registries [6,7]. These limitations may contribute to delayed or absent access to TPE for patients with ASFA category I indications [6,8].”

**4. Condensed Egyptian Context (Reduced from ~150 words to ~100 words)**

**Original:**

*"* Egypt, a lower-middle-income country with a population exceeding 110 million, has witnessed progressive expansion of apheresis services across tertiary institutions. However, published Egyptian data on TPE outcomes remain remarkably scarce. Only one adult multi-system cohort from Tanta University (n=64, 2016) and one renal-focused series from Cairo University (n=308, 2018) have been reported to date [13, 14]. Pediatric experiences, while documented, are not generalizable to adult populations [15, 16]. No large-scale Egyptian study has systematically characterized TPE practice across multiple organ systems in adults, nor has any study identified independent predictors of treatment response and mortality in the Egyptian context. The sole adult multi-system cohort is now nearly a decade old, predating recent ASFA guideline updates and significant advances in apheresis technology [13]."

**Revised:**

*"* Egypt, a lower-middle-income country, has witnessed progressive expansion of apheresis services, yet published data on adult TPE outcomes remain scarce. Only one adult multi-system cohort (n=64, 2016) and one renal-focused series (n=308, 2018) have been reported [13,14]. Pediatric experiences have also been documented but are not directly generalizable to adults [15,16]. No large-scale Egyptian study has systematically characterized TPE practice across multiple organ systems or identified independent predictors of treatment response and mortality. The only previous adult multi-system cohort is nearly a decade old and predates recent ASFA updates and advances in apheresis technology [13]."

**5. Condensed Evidence Gap and Rationale (Reduced from ~300 words to ~200 words)**

**Original:**

*"*This knowledge deficit carries tangible clinical consequences. Without region-specific evidence, Egyptian clinicians must extrapolate from North American, European, or Asian cohorts, populations with different genetic backgrounds, disease spectra, healthcare delivery models, and resource availability. Such extrapolation is precarious. Resource-constrained settings frequently necessitate adaptive practices: modified replacement fluid protocols (e.g., crystalloid substitution when albumin is unavailable), extended inter-session intervals due to equipment shortages, alternative vascular access strategies, and triage decisions that prioritize patients with the highest likelihood of benefit [7, 17]. Whether these adaptations compromise efficacy or safety remains unknown. Furthermore, the absence of baseline epidemiological data precludes benchmarking against international standards, impedes quality improvement initiatives, and weakens advocacy for resource allocation at institutional and ministerial levels [18]."

**Revised:**

" Without region-specific evidence, Egyptian clinicians must extrapolate from North American, European, or Asian cohorts, which differ in disease spectra, healthcare delivery models, and resource availability. Resource-constrained settings may require adaptive practices, including modified replacement-fluid protocols, extended inter-session intervals, alternative vascular access strategies, and triage based on anticipated benefit **[7,17].** Whether such adaptations compromise efficacy or safety remains unknown. The absence of baseline epidemiological data also limits benchmarking against international standards, quality improvement, and evidence-based resource allocation **[18]**.”

**3. Methodology**

**Reviewer comment:**

*“*Merge repetitive methodological descriptions*”.*

**Response:**
 We thank the reviewer for this suggestion. We have carefully reviewed the Methods section for potential redundancies. Upon examination, we believe that the methodological descriptions we provide are **complementary and essential** rather than repetitive, as each serves a distinct purpose:

| Methodological Component | Purpose | Distinctness |
| --- | --- | --- |
| Study design and population description | Defines the cohort, setting, and eligibility criteria | Essential for understanding the study scope |
| Clinical and anthropometric assessment | Describes how patients were evaluated clinically | Distinct from laboratory methods |
| Laboratory investigations | Describes specific assays and laboratory procedures | Distinct from clinical assessment |
| TPE procedure and devices | Describes the technical aspects of the intervention | Essential for reproducibility |
| Outcome measures | Defines primary, secondary, and exploratory outcomes | Distinct from procedural methods |
| Disease-specific response criteria | Provides condition-specific outcome definitions | Essential for interpreting disease-specific results |
| Statistical analysis | Describes analytical approaches | Distinct from clinical and procedural methods |

**Why these are complementary rather than repetitive:**

1. **Clinical vs. laboratory vs. procedural methods** describe fundamentally different aspects of the study, patient evaluation, laboratory testing, and treatment delivery. Each requires separate description for reproducibility.
2. **Outcome definitions and statistical methods** serve different purposes—one defines what was measured, the other describes how it was analyzed.
3. **Disease-specific response criteria** are provided separately for each condition (TTP, GBS, MG, SLE, etc.), which is necessary given the heterogeneous nature of our cohort. Combining these into a single generic description would reduce clinical interpretability.

**However, we have identified and merged one area where descriptions were partially duplicated:**

- **ADAMTS13, NGAL, and cystatin C laboratory protocols** originally appeared in two separate paragraphs with similar centrifugation, storage, and assay procedures. These have been consolidated into a single unified description in the Laboratory Investigations subsection.

**Manuscript Revisions :**

***Merged Laboratory Protocol Descriptions:***

**Original (two separate descriptions):**

*"* For patients with suspected thrombotic thrombocytopenic purpura, ADAMTS13 activity and inhibitor titers were measured when clinically available. Samples were collected in 3.2% sodium citrate tubes, centrifuged at 2500 ×g for 15 minutes at 4°C, and plasma was stored at -80°C until batch analysis. ADAMTS13 activity was determined using a fluorescence resonance energy transfer assay (Technozym ADAMTS-13 Activity ELISA, Technoclone, Vienna, Austria). Severe deficiency was defined as activity <10%."

" For biomarker substudies (n=34 consenting patients with thrombotic microangiopathies), serum neutrophil gelatinase-associated lipocalin and cystatin C were measured within 24 hours of TPE initiation. Samples were processed within 30 minutes of collection by centrifugation at 2500 rpm for 15 minutes at 4°C (Eppendorf 5702R, Hamburg, Germany). Aliquots were stored at -80°C (Thermo Scientific Forma 900 series, Waltham, MA, USA) until batch analysis. Serum NGAL and cystatin C concentrations were determined using commercially available enzyme-linked immunosorbent assay kits (Sunred Biological Technology Co., Ltd, Shanghai, China), following manufacturer protocols validated per Clinical and Laboratory Standards Institute EP15-A3 guidelines."

**Revised (merged into a single description):**

*"* For patients with suspected thrombotic thrombocytopenic purpura, ADAMTS13 activity and inhibitor titers were measured when available. Plasma was collected in 3.2% sodium citrate tubes, centrifuged at 2500 ×g for 15 minutes at 4°C, stored at −80°C, and analyzed in batches using a fluorescence resonance energy transfer assay (Technozym ADAMTS-13 Activity ELISA, Technoclone, Vienna, Austria). Severe ADAMTS13 deficiency was defined as activity <10%. In the metabolic group, fasting triglycerides, total cholesterol, LDL cholesterol, and HDL cholesterol were measured by enzymatic colorimetric methods on the Cobas 8000 analyzer. In the biomarker substudy, serum NGAL and cystatin C were measured within 24 hours of TPE initiation in 34 consenting patients with thrombotic microangiopathies. Samples were centrifuged at 2500 rpm for 15 minutes at 4°C, aliquoted, and stored at −80°C until batch analysis. NGAL and cystatin C were measured using commercial ELISA kits (Sunred Biological Technology Co., Ltd., Shanghai, China) according to manufacturer protocols validated per CLSI EP15-A3 guidelines. Absorbance was measured using a BioTek ELx808 microplate reader (Agilent Technologies, Santa Clara, CA, USA). Intra- and inter-assay coefficients of variation were <10% for both assays."

**4. Subgroup analyses:**

**Reviewer comment:**

*"* Move extensive subgroup analyses to Supplementary Material*."*

**Response:**

We respectfully disagree with the reviewer's suggestion to move subgroup analyses to Supplementary Material. We have considered this request carefully and believe the subgroup data should remain in the main text for the following reasons:

1. **PLOS ONE has no length restrictions.** The journal's guidelines state "Manuscripts can be any length. There are no restrictions on word count, number of figures, or amount of supporting information." Therefore, there is no requirement to move content to supplementary materials for space reasons.
2. **The subgroup analyses are not "extensive"** they are presented as focused, disease-specific analyses that directly address the study's primary and secondary objectives. The data occupy only a small proportion of the manuscript (Tables 5, 7, 8, 9, 12 and associated text).
3. **These findings are clinically important and central to the manuscript's contribution:**
   - **TTP subgroup:** PLASMIC score and treatment urgency predictors (Table 9) provide actionable guidance for clinicians
   - **GBS subgroup:** Hughes score and timing predictors (Table 12) directly inform clinical decision-making
   - **SLE subgroup:** Phenotype-dependent outcomes (Table 7) are clinically relevant for patient counseling
   - **DAH subgroup:** The 100% mortality finding (Table 8) is a critical safety signal.
4. **The reviewer has already addressed related concerns** in Major Comments 4–7 (statistical modeling, multiple comparisons, risk model, and disease heterogeneity). We have responded to each of these by:
   - Adding model diagnostics and internal validation (Comment 4)
   - Acknowledging the exploratory nature of subgroup findings (Comment 5)
   - Labeling the three-tier model as hypothesis-generating (Comment 6)
   - Justifying disease heterogeneity and adding stratified reporting (Comment 7)

Moving subgroup analyses to supplementary would contradict these responses by hiding the very evidence that supports the exploratory findings.

5. **Supplementary material is typically used for** large datasets, additional methodological details, or secondary analyses that are not central to the main findings. Our subgroup analyses are central to the study's contribution in a setting where no prior data exist.

**5. Long sentences :**

**Reviewer comment:**

*"* Shorten several overly long sentences for readability*."*

**Response:**

We agree with the reviewer that readability is improved by shortening overly long sentences. In the course of addressing the reviewer's previous comments, particularly the major request to reduce manuscript length by approximately 30% (Comment 1) and the minor request to shorten the Introduction (Minor Comment 2), we have systematically identified and shortened overly long sentences throughout the manuscript.

Specifically, we have:

1. **Broken down compound sentences** exceeding 40 words into shorter, clearer statements
2. **Removed redundant clauses** that added length without adding meaning
3. **Restructured complex sentences** into direct subject-verb-object constructions
4. **Replaced lengthy descriptive phrases** with concise alternatives

We have reviewed the revised manuscript and verified that all sentences now fall within a readable length. We believe the current text balances scientific rigor with readability.

**Response to Reviewer #2**

.

**1. Overinterpretation of Underpowered Subgroup Analyses**

**Reviewer comment:**

*“*Several disease-specific multivariate models have events-per-variable (EPV) ratios well below the conventional threshold of 10:

- TTP complete remission model (Table 9): EPV = 3.9 (27 events, 7 variables in complete-case analysis)
- GBS poor functional outcome model (Table 12): EPV ≈ 2.2 (13 events, 6 variables)
- SLE subgroup (Table 7): n=23 with 12 deaths

The authors acknowledge this limitation in the text but still present the findings with relatively strong language ("independent predictors," "confirming," "validating"). The abstract also states these findings without sufficient caveats.

**Action Required:**

1. Move the TTP and GBS multivariate models to supplementary material or clearly label them as "exploratory" in the main text with prominent caveats
2. Revise the abstract to state: "In exploratory analyses, PLASMIC score ≥6 and time to TPE ≤2 days were associated with complete remission in TTP, and Hughes score ≥4 and time to TPE >7 days were associated with poor functional outcome in GBS. These findings require external validation before clinical application."
3. Add a dedicated "Exploratory Subgroup Analyses" subsection in Results with a clear statement that these findings are hypothesis-generating
4. Consider whether to present the SLE subgroup (n=23) as a separate table or merge it into the main results with appropriate caveats”

*”***Response:**
 We thank the reviewer for this important methodological critique. We agree that the limited number of events in the TTP and GBS subgroup models results in low events-per-variable ratios and limits the reliability and generalizability of these estimates. We have therefore revised the manuscript to explicitly classify these analyses as exploratory and hypothesis-generating, removed stronger confirmatory language, and emphasized the need for external validation.

***Specifically***:

1. The TTP and GBS models remain in the main text but are now clearly identified as exploratory in the Results, with their EPV ratios and limitations explicitly reported. The language has been downgraded from "supports robustness" to cautious statements about a similar direction of associations.
2. The Abstract has been revised to describe these associations as exploratory and to state that they require external validation before clinical application.
3. A dedicated "Exploratory Subgroup Analyses" subsection has been added to the Results.
4. The SLE subgroup (n=23) has been retained in the main text but is now explicitly identified as exploratory because of its limited sample size.
5. The Discussion has been revised to emphasize that these subgroup findings should not be interpreted as confirmatory or directly translated into clinical practice.

**Manuscript Revisions:**

***1. Abstract***

**Original:**

" In TTP, PLASMIC score ≥6 and time to TPE ≤2 days predicted complete remission; in GBS, Hughes score ≥4 and time to TPE >7 days predicted poor functional outcome."

**Revised: (Add in Bold)**

"**In exploratory analyses,** PLASMIC score ≥6 and time to TPE ≤2 days **were associated with** complete remission in TTP, while Hughes score ≥4 and time to TPE >7 days **were associated *with*** poor functional outcome in GBS. **These findings require external validation before clinical application.**. "

***2. Results - Exploratory Subgroup Analyses Subsection (New)***

**Added:**

A dedicated “Exploratory Subgroup Analyses” subsection has been added to the Results section, immediately before the disease-specific subgroup analyses. The following statement was added:

**“The following disease-specific subgroup analyses were considered exploratory because of limited subgroup sample sizes and, where applicable, low events-per-variable ratios. These findings are hypothesis-generating and require validation in independent cohorts.”**

***3. TTP Section (Table 9) - Revised with Downgraded Language***

**Original:**

"The complete-case analysis for the TTP multivariate model (n=34 with ADAMTS13 available, 27 complete remission events, 7 candidate variables) yielded an events-per-variable ratio of 3.9, below the conventional threshold of 10. Results should therefore be interpreted as exploratory and hypothesis-generating, and the model requires external validation in larger TTP cohorts. However, the consistency of findings across three analytical approaches (complete-case, multiple imputation, and models excluding ADAMTS13) supports the robustness of the primary predictors."

**Revised:(added in bold)**

"The complete-case TTP multivariate model included 27 complete-remission events and 7 candidate variables **(EPV=3.9),** **below the conventional threshold of 10. These findings are therefore considered exploratory and hypothesis-generating and require validation in larger independent TTP cohorts. Similar directions of association were observed across the sensitivity analyses, but this consistency does not overcome the limitations imposed by the small number of events*.***. "

***4. GBS Section (Table 12) - Revised with Downgraded Language***

**Original:**

"This multivariate model included 13 poor outcome events (24.1% of 54 patients) and 6 candidate predictors, yielding an events-per-variable ratio of approximately 2.2, which is below the conventional threshold of 10. These results should therefore be interpreted as exploratory and hypothesis-generating, requiring external validation in larger GBS cohorts. A sensitivity analysis using the original threshold of ≥3 yielded consistent results (data not shown), confirming robustness of the direction of effect despite the limited sample size."

**Revised:**

"The GBS multivariate model included 13 poor functional outcome events and 6 candidate predictors **(EPV approximately 2.2), substantially below the conventional threshold of 10. These findings are therefore considered exploratory and hypothesis-generating and require validation in larger independent GBS cohorts.** **A sensitivity analysis using the original threshold of ≥3 showed a similar direction of association, although the limited number of events warrants cautious interpretation*.*** "

***5. Discussion - TTP/GBS Subgroup Limitations***

**Original:**

"Importantly, the TTP complete remission model (EPV 3.9) and GBS poor outcome model (EPV ≈2.2) had events-per-variable ratios below conventional thresholds. These findings should therefore be considered hypothesis-generating. Confirmatory studies with larger sample sizes are needed before these results can be translated into clinical practice guidelines."

**Revised: (added in bold)**

"Importantly, the TTP complete remission model (EPV 3.9) and GBS poor outcome model (EPV ≈2.2) had events-per-variable ratios substantially below conventional thresholds. **These findings are therefore considered exploratory and hypothesis-generating. They should not be interpreted as confirmatory or directly translated into clinical practice without validation in larger independent cohorts.**.

***7. Table 9 and Table 12 - Footnote Additions***

**Table 9 (TTP) - Added to footnote:**

"**These findings are exploratory and hypothesis-generating (EPV=3.9); external validation required**. "

**Table 12 (GBS) - Added to footnote:**

"**These findings are exploratory and hypothesis-generating (EPV≈2.2); external validation required**. "

**2. Three-Tier Risk Model**

**Reviewer comment:**

*“*The authors propose a three-tier risk stratification model (Section 14) based on 'synthesizing the multivariate regression, ROC curve analysis, Kaplan-Meier survival estimates, and indication-specific mortality data.' However:

- The model is described as 'developed post-hoc following completion of primary data analysis'
- It has 'undergone only internal validation (bootstrap resampling)' and 'has not been externally validated'
- The development methodology is not sufficiently detailed to be reproducible
- No formal model performance metrics (calibration, discrimination in the validation sample) are provided
- The model combines multiple data sources in a way that may lead to overfitting

**Action Required:**

Either:

1. Move the three-tier model to supplementary material with a clear statement that it is hypothesis-generating, OR
2. Provide a much more detailed development methodology including:
   - How the tiers were derived (exact algorithm or rules)
   - Performance metrics (calibration, discrimination) in the derivation cohort
   - Internal validation results (bootstrap-corrected performance).”

**Response:**
 We thank the reviewer for this important methodological critique. We agree that the three-tier risk stratification model was developed post-hoc and that its derivation and validation require clearer description. We have therefore retained the model in the main text as an exploratory, hypothesis-generating **risk stratification framework**, while clarifying its derivation, reporting the relevant performance measures of the underlying models, and explicitly stating that it is not validated for clinical use.

Specifically:

- **Derivation methodology:** We have clarified the four sources of information used to construct the framework—multivariate mortality predictors, Kaplan–Meier survival estimates, ROC-derived thresholds, and indication-specific mortality estimates—and have explicitly described the criteria used to define each risk tier.
- **Performance assessment:** We have reported the goodness-of-fit and discrimination measures available for the underlying mortality models and ROC-derived component predictors, including Nagelkerke R², Hosmer–Lemeshow testing, and optimism-corrected AUCs following bootstrap resampling.
- **Internal validation:** We have clarified that bootstrap resampling with 1,000 replicates represents internal validation of the underlying models and component predictors and does not constitute external validation of the three-tier framework.
- **Exploratory status:** The three-tier framework is now explicitly described as post-hoc, exploratory, and hypothesis-generating throughout the Results and Discussion.
- **Clinical applicability:** We have explicitly stated that the framework has not undergone external validation and should not be used for clinical decision-making or patient prognostication until prospectively validated in independent cohorts.

**Manuscript Revisions:**

***1. Section 14 - Title and Opening Revised***

**Original Title:**

"A Three-Tier Clinical Risk Stratification Model"

**Revised Title:**

"**Exploratory Three-Tier Risk Stratification Framework**"

**Original Opening:**

"The following three-tier risk stratification model was developed post-hoc following completion of primary data analysis."

**Revised Opening:**

"The following **exploratory** three-tier risk stratification framework was developed post-hoc following completion of the primary analyses**.** "

***2. Section 14 - Derivation Methodology Revised (Explicit Rules Added)***

**Original:**

"The model was derived by synthesizing four data sources: (1) multivariate logistic regression coefficients for independent mortality predictors (Table 10); (2) Kaplan-Meier 90-day survival estimates by diagnostic group (Figure 1); (3) ROC-derived laboratory cut-offs for mortality risk (Figure 3); and (4) indication-specific unadjusted mortality odds ratios (Figure 2). Tiers were defined based on the convergence of these data sources, with Tier 1 representing conditions with observed mortality >25% or extreme odds ratios, Tier 2 representing observed mortality of 10-20%, and Tier 3 representing observed mortality <5%."

**Revised** **(with bold additions):**

"The framework was constructed by synthesizing four sources of evidence: (1) multivariate logistic regression findings for independent mortality predictors (Table 10); (2) Kaplan-Meier estimates of 90-day survival by diagnostic group (Figure 1); (3) ROC-derived cut-off values for selected laboratory predictors (Figure 3); and (4) indication-specific mortality estimates (Figure 2)**.** **Because these components were synthesized post-hoc rather than combined through a prespecified predictive equation or formally validated scoring system, the framework should be considered hypothesis-generating rather than a validated prediction model.** **The tier definitions were based on the following explicit rules:** **Tier 1 (High Risk):** Conditions with observed mortality >25% or extreme odds ratios (DAH: 100%, OR 56.32; CAPS: 42.9%, OR 3.98; AMR: 28.6%, OR 4.21), OR patients in the renal diagnostic group with concurrent mechanical ventilation, creatinine >2.5 mg/dL, and hemoglobin <8 g/dL (estimated mortality >60% from the multivariate model). **Tier 2 (Intermediate Risk):** Conditions with observed mortality 10-20% (TTP: 14.8%; GBS with Hughes ≥4 or delayed TPE: 13.0%; MG crisis: 13.3%; ADEM: 16.7%). **Tier 3 (Low Risk):** Conditions with observed mortality <5% (AHA: 0%; NMO: 0%; pre-transplant desensitization: 0%; severe hypertriglyceridemia: 0%; CIDP: 0%; transverse myelitis: 0%). …. "

***3. Section 14 - Internal Validation Clarified***

**Original:**

"It has undergone only internal validation (bootstrap resampling with 1,000 replicates) and has not been externally validated in independent cohorts."

**Revised: (with bold additions):**

" **Internal bootstrap validation was performed for the underlying statistical models and ROC-derived component predictors; optimism-corrected AUCs were reported where applicable (creatinine: 0.79; hemoglobin: 0.71; platelet count: 0.65). This does not constitute validation of the three-tier framework itself. The framework has not been externally validated in independent cohorts.** "

***4. Discussion - Limitations (Risk Model Limitation Revised)***

**Original:**

"The three-tier risk model was developed post-hoc in a single-center cohort without external validation. While internal validation using bootstrap resampling (1,000 replicates) supported the stability of the model, this does not substitute for external validation in independent cohorts. The model should therefore be considered exploratory and hypothesis-generating; it is not validated for clinical use and should not guide patient management decisions until prospectively validated."

**Revised:(with bold additions):**

"The three-tier risk framework was developed post-hoc in a single-center cohort without external validation**.** **Bootstrap resampling (1,000 replicates) was performed for the underlying statistical models and component predictors, but this does not constitute validation of the three-tier framework itself. The framework should therefore be considered exploratory and hypothesis-generating; it is not validated for clinical use and should not guide patient management decisions until prospectively validated in independent cohorts.** "

***5. Methods - Statistical Analysis (Clarified)***

**Revised:**

"The three-tier mortality risk stratification framework was developed post-hoc following completion of primary data analysis. **This framework represents a hypothesis-generating synthesis of findings from multivariate logistic regression (independent predictors), Kaplan-Meier survival analysis (90-day survival probabilities), receiver operating characteristic curve analysis (optimal cut-off values), and the indication-specific forest plot (unadjusted odds ratios). The framework is presented as an exploratory synthesis requiring prospective validation and should not be interpreted as a validated prediction tool.** "

**3. A clear statement that the model is not validated for clinical use**

**Reviewer comment:**

“Add a prominent limitation: "The three-tier model was developed in a single-center cohort without external validation. It should be considered exploratory and hypothesis-generating until validated in independent cohorts.".

**Response:**
 We thank the reviewer for this important clarification. We agree that a prominent limitation statement regarding the three-tier model's lack of clinical validation is essential. This has been addressed in our response to Comment 2, and the limitation is now prominently stated in the Discussion - Limitations section exactly as the reviewer recommended.

**Confirmation of Existing Revisions:**

| Required Addition | Status | Location |
| --- | --- | --- |
| "The three-tier model was developed in a single-center cohort without external validation" | Already present | Discussion - Limitations |
| "It should be considered exploratory and hypothesis-generating until validated in independent cohorts" | Already present | Results - Section 14; Discussion - Limitations |
| Statement that model is not validated for clinical use | Already present | Results - Section 14; Discussion - Limitations |
| Statement that model should not guide patient management | Already present | Results - Section 14; Discussion - Limitations |

**Manuscript Revisions**

***Discussion - Limitations (Revised with Bold Additions):***

*"* **Sixth, the three-tier risk framework was developed post-hoc in a single-center cohort without external validation. It should be considered exploratory and hypothesis-generating until validated in independent cohorts.** While bootstrap resampling (1,000 replicates) was performed for the underlying statistical models and component predictors, this does not constitute validation of the three-tier framework itself. The framework should therefore be considered exploratory and hypothesis-generating; it is not validated for clinical use and should not guide patient management decisions until prospectively validated in independent cohorts……….. "*

***Results - Section 14 (Exploratory Status Strengthened):***

" **This framework is presented for hypothesis generation only. It has not been validated for clinical use and should not guide patient management decisions. Prospective validation in independent cohorts is required before any clinical application.** "

**4. Patient Classification: TTP and SLE Assignment**

**Reviewer comment:**

“Current Issue: The authors classify TTP patients with predominant renal involvement under renal indications (n=41) and those with predominant hematologic manifestations under hematologic indications (n=13). This is a reasonable approach but requires more justification.

**Action Required:**

1. Add a brief explanation of why this classification was chosen: "This classification preserves the integrity of between-group comparisons (renal vs. neurologic vs. hematologic) based on the primary management pathway, while allowing disease-specific subgroup analyses (TTP, SLE, GBS, MG) to be performed on unified cohorts across groups."
2. Acknowledge the limitation: "This classification may introduce some misclassification bias, as TTP is a multi-system disease, but it reflects real-world practice where patients are managed primarily by the specialty responsible for their predominant manifestation."

**Response:**
We thank the reviewer for this important methodological clarification. We agree that the classification of TTP and SLE patients based on predominant clinical presentation requires clear justification and acknowledgment of its limitations.

**We confirm that the justification/explanation of why this classification was chosen is already present in the Methods section.** However, the **acknowledgment of misclassification bias as a limitation** has not been explicitly stated. We have now added this acknowledgment to the Methods section immediately following the existing classification explanation.

**Manuscript Revisions:**

***Methods - Classification of Patients with Multi-system Diseases (Revised with Bold Addition):***

**Original:**

"This classification approach preserves the integrity of between-group comparisons (renal vs. neurologic vs. hematologic) while allowing disease-specific subgroup analyses (TTP, SLE, GBS, MG) to be performed on unified cohorts across groups. Table 2 provides the complete breakdown of specific indications within each diagnostic group."

**Revised:**

" This classification approach preserves the integrity of between-group comparisons (renal vs. neurologic vs. hematologic) while allowing disease-specific subgroup analyses (TTP, SLE, GBS, MG) to be performed on unified cohorts across groups. **We acknowledge that this classification may introduce some misclassification bias, as both TTP and SLE are multi-system diseases. However, this approach reflects real-world practice where patients are managed primarily by the specialty responsible for their predominant manifestation, and it preserves the integrity of between-group comparisons while allowing disease-specific subgroup analyses on unified cohorts.** Table 2 provides the complete breakdown of specific indications within each diagnostic group."

**Confirmation of Existing Revisions:**

| Requirement | Status | Location |
| --- | --- | --- |
| Justification of classification approach | Already present | Methods - Classification of Patients |
| "Preserves integrity of between-group comparisons" | Already present | Methods - Classification of Patients |
| "Allows disease-specific subgroup analyses on unified cohorts" | Already present | Methods - Classification of Patients |
| Acknowledgment of misclassification bias limitation | **Added** | Methods - Classification of Patients |

**5. Propensity Score Matching: Limited Overlap and Generalizability**

**Reviewer comment:**

*“*Current Issue: The FFP vs. albumin propensity score matching achieved only 50% match rate for FFP recipients, raising questions about generalizability. The authors explain this appropriately, but could strengthen the discussion.

**Action Required:**

1. Add a clearer statement: 'The limited overlap between FFP and albumin recipients confirms that these populations were fundamentally different by design, with FFP reserved for critically ill patients requiring factor replacement. The matched sample therefore represents the subset of patients where clinical equipoise existed, allowing unbiased estimation of the treatment effect.'
2. Acknowledge that the results may not generalize to patients who could not be matched (the sickest FFP patients).”

**Response:**
We thank the reviewer for this important methodological consideration. We agree that the limited overlap in propensity score matching and its implications for generalizability warrant clearer acknowledgment.

**We confirm that the first requested statement is already present in the manuscript** (Results - Section 16 and Discussion). However, the **acknowledgment that results may not generalize to unmatched FFP patients** has been added to the Discussion.

**Manuscript Revisions:**

***Discussion - Replacement Fluid (Revised with Bold Addition):***

**Original:**

" The comparison of fresh frozen plasma versus albumin as replacement fluid warrants specific comment regarding methodological rigor. Propensity score matching achieved balance on all measured covariates, but the 50% match rate for FFP recipients initially raises questions about generalizability. ………. The matched sample therefore represents the subset of patients with sufficient covariate overlap for adjusted comparison; however, residual confounding cannot be excluded."

**Revised (with bold addition):**

"The comparison of fresh frozen plasma versus albumin as replacement fluid warrants specific comment regarding methodological rigor. Propensity score matching achieved balance on all measured covariates, but the 50% match rate for FFP recipients initially raises questions about generalizability. ……….. The matched sample therefore represents the subset of patients with sufficient covariate overlap for adjusted comparison; however, residual confounding cannot be excluded. **We further acknowledge that the findings may not generalize to the 50% of FFP recipients who could not be matched, the sickest patients requiring FFP for life-saving factor replacement. The observed outcomes in this unmatched subgroup may differ from those in the matched sample, and our conclusions should therefore be interpreted as applicable to the subset of patients where clinical equipoise existed, rather than to all FFP recipients.** "

**Confirmation of Existing Revisions:**

| Requirement | Status | Location |
| --- | --- | --- |
| "Limited overlap confirms populations fundamentally different by design" | Already present | Results - Section 16; Discussion |
| "FFP reserved for critically ill patients requiring factor replacement" | Already present | Results - Section 16; Discussion |
| "Matched sample represents subset where clinical equipoise existed" | Already present | Results - Section 16; Discussion |
| Acknowledgment results may not generalize to unmatched (sickest FFP patients) | **Added** | Discussion |

**6. Adverse Event Reporting: Patient vs. Session Level**

**Reviewer comment:**

“ Current Issue: The authors report adverse events at both patient level and session level, which is appropriate. However, the infection rate (11.7% of patients) appears high compared to some international series.

**Action Required:**

1. Add a brief discussion: 'The infection rate of 11.7% in our cohort is higher than some international reports (5-8%) but reflects the cumulative immunosuppressive effect of repeated plasma exchange, particularly immunoglobulin depletion, in the context of severe underlying disease states in a resource-limited setting.'
2. Consider adding the infection rate per session for comparison with international data.”

**Response:**
We thank the reviewer for this important observation regarding the infection rate in our cohort. We agree that the 11.7% patient-level infection rate warrants contextualization. We have made the following revisions:

1. **Added the infection rate per session** (2.4%) to the Results section to allow comparison with international data
2. **Added explicit acknowledgment of the resource-limited setting** as a contributing factor to the Discussion

**Manuscript Revisions:**

***1. Results - Section 4 (Added Session-Level Infection Rate):***

**Original:**

" Between treatment sessions, infections occurred in 11.7% of patients (26 of 221), while bleeding from the dialysis catheter site was rare (0.4%, 1 patient). This infection rate, while higher than some international reports, reflects the cumulative immunosuppressive effect of repeated plasma exchange, particularly immunoglobulin depletion, in the context of severe underlying disease states."

**Revised (with bold addition):**

"Between treatment sessions, infections occurred in 11.7% of patients (26 of 221)**; at the session level, infections occurred in 2.4% of TPE sessions (26 of 1,105 sessions)**, while bleeding from the dialysis catheter site was rare (0.4%, 1 patient). This infection rate, while higher than some international reports, reflects the cumulative immunosuppressive effect of repeated plasma exchange, particularly immunoglobulin depletion, in the context of severe underlying disease states."

***2. Discussion - Adverse Events (Added Resource-Limited Context):***

**Original:**

" The higher rate of inter-session infections (11.7% of patients) reflects the cumulative immunosuppressive effect of repeated plasma exchange, particularly immunoglobulin depletion, in the context of severe underlying disease states, underscoring the importance of infection surveillance and prophylactic measures as emphasized in published consensus guidelines [44]."

**Revised (with bold additions):**

" The higher rate of inter-session infections (11.7% of patients; **2.4% per session**) reflects the cumulative immunosuppressive effect of repeated plasma exchange, particularly immunoglobulin depletion, in the context of severe underlying disease states **in a resource-limited setting**, underscoring the importance of infection surveillance and prophylactic measures as emphasized in published consensus guidelines [44]. **This rate is higher than some international reports (5-8% at patient level), which may reflect differences in patient acuity, infection prevention protocols, and healthcare infrastructure.** "

**Confirmation of Existing Revisions:**

| Requirement | Status | Location |
| --- | --- | --- |
| Infection rate 11.7% higher than international reports (5-8%) | Added | Discussion |
| Reflects cumulative immunosuppressive effect | Already present | Results - Section 4; Discussion |
| Immunoglobulin depletion context | Already present | Results - Section 4; Discussion |
| Resource-limited setting context | **Added** | Discussion |
| Infection rate per session for comparison | **Added** | Results - Section 4 |

**7. Missing Data and Imputation**

**Reviewer comment:**

“Current Issue: The authors report 3.7% overall missingness, with some variables (PLASMIC score components, ADAMTS13, lipid profile) having up to 12% missingness. They performed multiple imputation but present complete-case analysis as primary.

**Action Required:**

1. Briefly state that complete-case and imputed results were consistent
2. Acknowledge that missing data for ADAMTS13 (37% of TTP patients) is a limitation.”

**Response:**
We thank the reviewer for this important methodological consideration. We confirm that both requested actions have been addressed:

1. **Complete-case and imputed results consistency** is already explicitly stated in the Methods - Statistical Analysis section.
2. **ADAMTS13 missing data (37%)** is already acknowledged in the Discussion - Limitations, and we have strengthened this statement to explicitly mention the 37% missing rate.

**Manuscript Revisions:**

***1. Methods - Statistical Analysis (Already Present):***

" Overall, missingness in the dataset was 3.7%, ranging from 0 to 12% per variable. Variables with greater than 5% missingness included components of the PLASMIC score (12%), ADAMTS13 activity (11%), and lipid profile in the metabolic group (9%). Mortality status was complete for all 221 patients, including 32 patients who died during follow-up; no missingness occurred for the primary outcome. Multiple imputation by chained equations was performed with 20 imputed datasets and 10 iterations, using predictive mean matching for continuous variables and logistic regression for categorical variables. Pooled estimates from imputed datasets were calculated using Rubin's rules. **Complete-case analysis is presented as the primary analysis; sensitivity analyses confirmed robustness of findings across both approaches.** "

***2. Discussion - Limitations (Strengthened with Bold Addition):***

**Original:**

*"* The retrospective design also limited the availability of certain laboratory parameters (e.g., ADAMTS13 testing in only 63% of TTP patients), though sensitivity analyses confirmed the robustness of the PLASMIC score and treatment urgency as predictors*."*

**Revised:**

*"*The retrospective design also limited the availability of certain laboratory parameters **, including ADAMTS13 testing which was available in only 63% of TTP patients (37% missing)**. Though sensitivity analyses confirmed the robustness of the PLASMIC score and treatment urgency as predictors, **this missing data represents a limitation and may affect the generalizability of the TTP subgroup findings.** *"*

**Confirmation of Existing Revisions:**

| Requirement | Status | Location |
| --- | --- | --- |
| Complete-case and imputed results consistent | Already present | Methods - Statistical Analysis |
| ADAMTS13 missing data (37%) acknowledged as limitation | Strengthened | Discussion - Limitations |

**Finally**, we sincerely thank the Editor and both reviewers for their careful reading, insightful feedback, and constructive suggestions. Their comments greatly improved the clarity, balance, and scientific rigor of our manuscript. We carefully addressed each point, clarified methodological details, expanded on pharmacological and clinical interpretations, improved figures and tables, and refined the language.

We deeply respect the reviewers’ expertise, and we have benefited significantly from their input, which not only strengthened this work but will also inform our future research directions. We believe the revised version now provides a clearer, more comprehensive, and clinically meaningful contribution to science.

We hope the manuscript in its current form will be considered suitable for publication, and we kindly request your favorable consideration for acceptance.

With our best regards and gratitude,

*** Corresponding author:**

**Amira A. A. Othman**

Department: Internal Medicine.

Institute: Faculty of Medicine, Suez University.

City, State, Country: Suez, Suez, Egypt.

Postal code: 43511 | Tel: 00201003258097 |

E-mail: Amira.Othman@med.suezuni.edu.eg |

ORCID number: <https://orcid.org/0000-0002-8191-0035>
