# Supplementary material for: Therapeutic plasma exchange across multiple organ systems in an Egyptian tertiary center: A seven-year real-world cohort study
Source: PLoS One. 2026 Sep 11;21(9):e0358069. doi: 10.1371/journal.pone.0358069 (PMC13567810; doi:10.1371/journal.pone.0358069)
Supplement: Peer review history

# Author comment: Author response to Decision Letter 0

**DOI:** 10.1371/journal.pone.0358069.r001

**Transfer Alert**

This paper was transferred from another journal. As a result, its full editorial history (including decision letters, peer reviews and author responses) may not be present.

1 Jul 2026

# Peer review: Decision Letter 0

**DOI:** 10.1371/journal.pone.0358069.r002

**Authors:** Awais ALi

10 Aug 2026

Dear Dr. Othman,

Thank you for submitting your manuscript to PLOS ONE. After careful consideration, we feel that it has merit but does not fully meet PLOS ONE’s publication criteria as it currently stands. Therefore, we invite you to submit a revised version of the manuscript that addresses the points raised during the review process.

Please submit your revised manuscript by Oct 09 2026 11:59PM. If you will need more time than this to complete your revisions, please reply to this message or contact the journal office at plosone@plos.org. When you're ready to submit your revision, log on to https://www.editorialmanager.com/pone/ and select the 'Submissions Needing Revision' folder to locate your manuscript file.

- A letter that responds to each point raised by the academic editor and reviewer(s). You should upload this letter as a separate file labeled 'Response to Reviewers'.
- A marked-up copy of your manuscript that highlights changes made to the original version. You should upload this as a separate file labeled 'Revised Manuscript with Track Changes'.
- An unmarked version of your revised paper without tracked changes. You should upload this as a separate file labeled 'Manuscript'.

If you would like to make changes to your financial disclosure, please include your updated statement in your cover letter. Guidelines for resubmitting your figure files are available below the reviewer comments at the end of this letter.

If applicable, we recommend that you deposit your laboratory protocols in protocols.io to enhance the reproducibility of your results. Protocols.io assigns your protocol its own identifier (DOI) so that it can be cited independently in the future. For instructions see: https://journals.plos.org/plosone/s/submission-guidelines#loc-laboratory-protocols. Additionally, PLOS ONE offers an option for publishing peer-reviewed Lab Protocol articles, which describe protocols hosted on protocols.io. Read more information on sharing protocols at https://plos.org/protocols?utm_medium=editorial-email&utm_source=authorletters&utm_campaign=protocols.

As the corresponding author, your ORCID iD is verified in the submission system and will appear in the published article. PLOS supports the use of ORCID, and we encourage all coauthors to register for an ORCID iD and use it as well. Please encourage your coauthors to verify their ORCID iD within the submission system before final acceptance, as unverified ORCID iDs will not appear in the published article. *Only*  the individual author can complete the verification step; PLOS staff *cannot*  verify ORCID iDs on behalf of authors.

We look forward to receiving your revised manuscript.

Kind regards,

Dr. Awais ALi

Guest Editor

PLOS One

Journal Requirements:

When submitting your revision, we need you to address these additional requirements.

1. Please ensure that your manuscript meets PLOS ONE's style requirements, including those for file naming. The PLOS ONE style templates can be found at

https://journals.plos.org/plosone/s/file?id=wjVg/PLOSOne_formatting_sample_main_body.pdf and

https://journals.plos.org/plosone/s/file?id=ba62/PLOSOne_formatting_sample_title_authors_affiliations.pdf

2. We note that you have indicated that there are restrictions to data sharing for this study. For studies involving human research participant data or other sensitive data, we encourage authors to share de-identified or anonymized data. However, when data cannot be publicly shared for ethical reasons, we allow authors to make their data sets available upon request. For information on unacceptable data access restrictions, please see http://journals.plos.org/plosone/s/data-availability#loc-unacceptable-data-access-restrictions.

Before we proceed with your manuscript, please address the following prompts:

a) If there are ethical or legal restrictions on sharing a de-identified data set, please explain them in detail (e.g., data contain potentially identifying or sensitive patient information, data are owned by a third-party organization, etc.) and who has imposed them (e.g., a Research Ethics Committee or Institutional Review Board, etc.). Please also provide contact information for a data access committee, ethics committee, or other institutional body to which data requests may be sent.

b) If there are no restrictions, please upload the minimal anonymized data set necessary to replicate your study findings to a stable, public repository and provide us with the relevant URLs, DOIs, or accession numbers. Please see http://www.bmj.com/content/340/bmj.c181.long for guidelines on how to de-identify and prepare clinical data for publication. For a list of recommended repositories, please see https://journals.plos.org/plosone/s/recommended-repositories. You also have the option of uploading the data as Supporting Information files, but we would recommend depositing data directly to a data repository if possible.

Please update your Data Availability statement in the submission form accordingly.

3. Your ethics statement should only appear in the Methods section of your manuscript. If your ethics statement is written in any section besides the Methods, please delete it from any other section.

4. If the reviewer comments include a recommendation to cite specific previously published works, please review and evaluate these publications to determine whether they are relevant and should be cited. There is no requirement to cite these works unless the editor has indicated otherwise.

[Note: HTML markup is below. Please do not edit.]

Reviewers' comments:

Reviewer's Responses to Questions

**Comments to the Author**

1. Is the manuscript technically sound, and do the data support the conclusions?

Reviewer #1: Yes

Reviewer #2: Yes

**********

2. Has the statistical analysis been performed appropriately and rigorously?

Reviewer #1: Yes

Reviewer #2: Yes

**********

3. Have the authors made all data underlying the findings in their manuscript fully available?

The PLOS Data policy

Reviewer #1: Yes

Reviewer #2: Yes

**********

4. Is the manuscript presented in an intelligible fashion and written in standard English?

Reviewer #1: Yes

Reviewer #2: Yes

**********

Reviewer #1: Overall Assessment

This manuscript presents a retrospective seven-year cohort evaluating therapeutic plasma exchange (TPE) in an Egyptian tertiary center. The topic is clinically relevant because real-world evidence from low- and middle-income countries remains limited. The study includes 221 patients and evaluates efficacy, safety, and predictors of outcomes. While the work has merit, substantial revisions are required before publication.

Major Comments

1. Manuscript Length

The manuscript is considerably longer than necessary. The Introduction, Methods, and Discussion should be reduced by approximately 30%, removing repeated methodological descriptions and excessive technical details.

2. Study Objectives

The objectives are overly broad. Clearly distinguish primary, secondary, and exploratory objectives.

3. Study Design

The retrospective, single-center nature of the study should be emphasized more consistently, and causal language should be avoided.

4. Statistical Modeling

Multiple regression models were fitted despite only 32 mortality events. Additional information on model diagnostics, multicollinearity, calibration, and internal validation is required.

5. Multiple Comparisons

Numerous subgroup analyses increase the risk of type I error. Adjustment for multiple testing should be considered.

6. Risk Model

The proposed three-tier mortality model requires internal or external validation before being presented as a clinically useful model.

7. Disease Heterogeneity

Pooling renal, neurological, hematological, and metabolic diseases introduces substantial heterogeneity that should be better justified.

8. Clinical Response

Combining disease-specific outcomes into one composite response variable may reduce clinical interpretability.

Statistical Comments

Please report missing data percentages, model diagnostics, proportional hazards assumptions, multicollinearity assessment, ROC/calibration results where appropriate, and sensitivity analyses using the multiple imputation dataset.

Minor Comments

• Reduce the number of numerical values in the abstract.

• Shorten the Introduction by removing well-established background information.

• Merge repetitive methodological descriptions.

• Move extensive subgroup analyses to Supplementary Material.

• Shorten several overly long sentences for readability.

Strengths

• Largest reported adult Egyptian multi-system TPE cohort.

• Seven years of consecutive real-world experience.

• Clinically relevant outcome measures.

• Comprehensive safety assessment.

• Appropriate use of ASFA classification.

• Generally well-written manuscript.

Final Recommendation

The study addresses an important clinical question and provides valuable regional evidence. However, substantial revision is necessary to improve focus, strengthen the statistical reporting, reduce redundancy, and temper interpretation of exploratory findings.

Reviewer #2: Major Issues Requiring Resolution

1. Overinterpretation of Underpowered Subgroup Analyses

Current Issue: Several disease-specific multivariate models have events-per-variable (EPV) ratios well below the conventional threshold of 10:

TTP complete remission model (Table 9): EPV = 3.9 (27 events, 7 variables in complete-case analysis)

GBS poor functional outcome model (Table 12): EPV ≈ 2.2 (13 events, 6 variables)

SLE subgroup (Table 7): n=23 with 12 deaths

The authors acknowledge this limitation in the text but still present the findings with relatively strong language ("independent predictors," "confirming," "validating"). The abstract also states these findings without sufficient caveats.

Action Required:

Move the TTP and GBS multivariate models to supplementary material or clearly label them as "exploratory" in the main text with prominent caveats

Revise the abstract to state: "In exploratory analyses, PLASMIC score ≥6 and time to TPE ≤2 days were associated with complete remission in TTP, and Hughes score ≥4 and time to TPE >7 days were associated with poor functional outcome in GBS. These findings require external validation before clinical application."

Add a dedicated "Exploratory Subgroup Analyses" subsection in Results with a clear statement that these findings are hypothesis-generating

Consider whether to present the SLE subgroup (n=23) as a separate table or merge it into the main results with appropriate caveats

2. Three-Tier Risk Model: Development and Validation

Current Issue: The authors propose a three-tier risk stratification model (Section 14) based on "synthesizing the multivariate regression, ROC curve analysis, Kaplan-Meier survival estimates, and indication-specific mortality data." However:

The model is described as "developed post-hoc following completion of primary data analysis"

It has "undergone only internal validation (bootstrap resampling)" and "has not been externally validated"

The development methodology is not sufficiently detailed to be reproducible

No formal model performance metrics (calibration, discrimination in the validation sample) are provided

The model combines multiple data sources in a way that may lead to overfitting

Action Required:

Either move the three-tier model to supplementary material with a clear statement that it is hypothesis-generating, OR

Provide a much more detailed development methodology including:

How the tiers were derived (exact algorithm or rules)

Performance metrics (calibration, discrimination) in the derivation cohort

Internal validation results (bootstrap-corrected performance)

A clear statement that the model is not validated for clinical use

Add a prominent limitation: "The three-tier model was developed in a single-center cohort without external validation. It should be considered exploratory and hypothesis-generating until validated in independent cohorts."

Patient Classification: TTP and SLE Assignment

Current Issue: The authors classify TTP patients with predominant renal involvement under renal indications (n=41) and those with predominant hematologic manifestations under hematologic indications (n=13). This is a reasonable approach but requires more justification.

Action Required:

Add a brief explanation of why this classification was chosen: "This classification preserves the integrity of between-group comparisons (renal vs. neurologic vs. hematologic) based on the primary management pathway, while allowing disease-specific subgroup analyses (TTP, SLE, GBS, MG) to be performed on unified cohorts across groups."

Acknowledge the limitation: "This classification may introduce some misclassification bias, as TTP is a multi-system disease, but it reflects real-world practice where patients are managed primarily by the specialty responsible for their predominant manifestation."

5. Propensity Score Matching: Limited Overlap and Generalizability

Current Issue: The FFP vs. albumin propensity score matching achieved only 50% match rate for FFP recipients, raising questions about generalizability. The authors explain this appropriately, but could strengthen the discussion.

Action Required:

Add a clearer statement: "The limited overlap between FFP and albumin recipients confirms that these populations were fundamentally different by design, with FFP reserved for critically ill patients requiring factor replacement. The matched sample therefore represents the subset of patients where clinical equipoise existed, allowing unbiased estimation of the treatment effect."

Acknowledge that the results may not generalize to patients who could not be matched (the sickest FFP patients)

6. Adverse Event Reporting: Patient vs. Session Level

Current Issue: The authors report adverse events at both patient level and session level, which is appropriate. However, the infection rate (11.7% of patients) appears high compared to some international series.

Action Required:

Add a brief discussion: "The infection rate of 11.7% in our cohort is higher than some international reports (5-8%) but reflects the cumulative immunosuppressive effect of repeated plasma exchange, particularly immunoglobulin depletion, in the context of severe underlying disease states in a resource-limited setting."

Consider adding the infection rate per session for comparison with international data

7. Missing Data and Imputation

Current Issue: The authors report 3.7% overall missingness, with some variables (PLASMIC score components, ADAMTS13, lipid profile) having up to 12% missingness. They performed multiple imputation but present complete-case analysis as primary.

Action Required:

Briefly state that complete-case and imputed results were consistent

Acknowledge that missing data for ADAMTS13 (37% of TTP patients) is a limitation

**********

what does this mean?). If published, this will include your full peer review and any attached files.

If you choose “no”, your identity will remain anonymous but your review may still be made public.

**Do you want your identity to be public for this peer review?**  For information about this choice, including consent withdrawal, please see our Privacy Policy

Reviewer #1: No

Reviewer #2: No

**********

[NOTE: If reviewer comments were submitted as an attachment file, they will be attached to this email and accessible via the submission site. Please log into your account, locate the manuscript record, and check for the action link "View Attachments". If this link does not appear, there are no attachment files.]

To ensure your figures meet our technical requirements, please review our figure guidelines: https://journals.plos.org/plosone/s/figures

You may also use PLOS’s free figure tool, NAAS, to help you prepare publication quality figures: https://journals.plos.org/plosone/s/figures#loc-tools-for-figure-preparation.

NAAS will assess whether your figures meet our technical requirements by comparing each figure against our figure specifications.

# Author comment: Author response to Decision Letter 1

**DOI:** 10.1371/journal.pone.0358069.r003

17 Aug 2026

Point by Point Response

Response to Editorial Comments PLOS ONE Style Requirements

Editorial Comment #1

Journal Requirements:

“ Please ensure that your manuscript meets PLOS ONE's style requirements, including those for file naming. The PLOS ONE style templates can be found at:

• https://journals.plos.org/plosone/s/file?id=wjVg/PLOSOne_formatting_sample_main_body.pdf

• https://journals.plos.org/plosone/s/file?id=ba62/PLOSOne_formatting_sample_title_authors_affiliations.pdf . ”

Author Response:

We have carefully reviewed the PLOS ONE style templates and guidelines. We have reformatted our manuscript to meet all requirements. Specifically, we have:

1. File Format: Confirmed the manuscript is in DOCX format as required

2. Font: Used Arial 11pt font throughout (standard font)

3. Spacing: Set to double spacing throughout the manuscript

4. Page Numbers: Added page numbers to all pages

5. Line Numbers: Added continuous line numbers throughout the manuscript (not restarting on each page)

6. Title Page: Formatted as the first page with all required elements (title, authors, affiliations, corresponding author, short title)

7. Section Headers: Used consistent formatting for all section headers

8. No Footnotes: Verified no footnotes are present; all content is in main text or references

9. Single Column: Manuscript is in single column format

10. Reference Style: Verified references follow Vancouver style

11. File Naming: Renamed all files according to PLOS ONE conventions

Revised Manuscript Section:

No specific text revisions required for this comment. The entire manuscript has been reformatted to comply with PLOS ONE style requirements. The following changes have been made throughout:

Requirement Original Status Revised Status

Double spacing Checked Confirmed double spacing

Page numbers Missing Added to all pages

Continuous line numbers Missing Added throughout

File naming Non-standard Renamed per PLOS ONE conventions

Abstract headers Present NO CHANGE NEEDED (allowed by PLOS ONE)

________________________________________

Editorial Comment #2

Data Availability Statement:

“ We note that you have indicated that there are restrictions to data sharing for this study. For studies involving human research participant data or other sensitive data, we encourage authors to share de-identified or anonymized data. However, when data cannot be publicly shared for ethical reasons, we allow authors to make their data sets available upon request. For information on unacceptable data access restrictions, please see http://journals.plos.org/plosone/s/data-availability#loc-unacceptable-data-access-restrictions.

Before we proceed with your manuscript, please address the following prompts:

a) If there are ethical or legal restrictions on sharing a de-identified data set, please explain them in detail (e.g., data contain potentially identifying or sensitive patient information, data are owned by a third-party organization, etc.) and who has imposed them (e.g., a Research Ethics Committee or Institutional Review Board, etc.). Please also provide contact information for a data access committee, ethics committee, or other institutional body to which data requests may be sent.

b) If there are no restrictions, please upload the minimal anonymized data set necessary to replicate your study findings to a stable, public repository and provide us with the relevant URLs, DOIs, or accession numbers.

Please update your Data Availability statement in the submission form accordingly. “

Author Response:

We thank the PLOS ONE editorial office for bringing this issue to our attention. We have reviewed the journal's data-sharing requirements and have revised the Data Availability Statement to clearly explain why the individual-level dataset cannot be made publicly available.

The study is based on retrospective clinical records from 221 patients. The underlying individual-level dataset contains demographic, diagnostic, laboratory, treatment-related, and clinical outcome information. Although the analytical data were de-identified, the combination of variables, particularly for patients with uncommon TPE indications, could permit re-identification through linkage with institutional records. The study was conducted under institutional ethics approval and a waiver of informed consent, and the underlying patient-level data are subject to institutional and applicable data-protection restrictions.

Accordingly, we are unable to deposit the individual-level dataset in a public repository or make it freely downloadable. We have therefore provided a detailed Data Availability Statement in the revised manuscript explaining the restriction and specifying the institutional procedure through which scientifically justified data-access requests may be considered, subject to approval by the Zagazig University Institutional Review Board and applicable data-protection requirements.

We have provided complete contact information for data-access requests, including the Research Ethics Committee Office.

We understand that PLOS ONE requires an explicit editorial assessment when human participant data cannot be publicly deposited for ethical or legal reasons. We therefore respectfully request that the present case be considered for an exemption from public data deposition under the journal's data-sharing policy. We are willing to provide any additional documentation required by the editorial office to support this exemption.

Revised Manuscript Section:

Original Data Availability Statement:

The datasets generated and analyzed during the current study are not publicly available because they contain information that could compromise research participant privacy, and informed consent was waived for this retrospective study. De-identified data may be available from the corresponding author upon reasonable request, subject to approval from the Zagazig University Institutional Review Board and applicable Egyptian data protection regulations.

Revised Data Availability Statement: (Bold = Add)

“ The individual-level dataset generated and analyzed during the current study is not publicly available because it comprises retrospective human participant clinical data subject to institutional ethical and data-protection restrictions. The study was conducted under a waiver of informed consent, and public deposition or unrestricted external transfer of the underlying patient-level data was not authorized. Although the analytical dataset was de-identified, the combination of demographic, diagnostic, laboratory, treatment, and outcome variables may permit re-identification, particularly for patients with uncommon TPE indications, through linkage with institutional records. Accordingly, the individual-level dataset cannot be deposited in a public repository or made freely downloadable. De-identified data may be made available for scientifically justified requests, subject to review and approval by the Zagazig University Institutional Review Board and applicable data-protection requirements. Data-access requests may be submitted to the Research Ethics Committee Office, Zagazig University Faculty of Medicine, Zagazig, Egypt (email: irb@zu.edu.eg; phone: +20 55 230 3125)."

________________________________________

Editorial Comment #3

Ethics Statement Location:

“ Your ethics statement should only appear in the Methods section of your manuscript. If your ethics statement is written in any section besides the Methods, please delete it from any other section ”.

Author Response:

We have confirmed that the full ethics statement appears exclusively in the Methods section (under "Ethical Considerations"). We have revised the Declarations section to contain only a brief reference to the Methods section, eliminating any duplication.

Revised Manuscript Section:

Original Declarations Section (Ethics Statement – Keep full text):

This retrospective cohort study was approved by the Institutional Review Board of Zagazig University Faculty of Medicine (ZU-IRB #9357) on 27/03/2022 (initial approval; expired 27/03/2023), with renewal approved on 08/02/2026 (expires 08/02/2027). Data collection and analysis were completed before the initial approval expired on 27/03/2023; the 2026 renewal was obtained solely for manuscript preparation and journal submission requirements, as the study involved no ongoing patient contact or new data collection after the initial approval period. IRB approval was obtained retrospectively, as data from patients treated between 01/01/2016 and 01/12/2022 were extracted from existing medical records created during routine clinical care. The IRB permitted this under Egyptian national regulations (Ministerial Decree 296/2021) for minimal-risk retrospective research involving no direct patient contact.

Informed consent: Because this study involved only the analysis of de-identified, pre-existing medical records, with no direct patient contact or intervention, the requirement for written informed consent was waived by the Zagazig University IRB. No research consent was obtained from any patient or legally authorized representative. The reason written consent could not be obtained was the retrospective nature of the study: contacting all 221 patients (including 32 who died during follow-up) was not feasible without prospective patient contact, which this retrospective design specifically excluded. This determination is consistent with Egyptian national regulations (Ministerial Decree 296/2021, Article 18) and the Declaration of Helsinki (2013 revision), which permit waiver of consent for retrospective research using existing medical records when the research involves no more than minimal risk and cannot practicably be carried out without the waiver.

Data anonymization and confidentiality: Patient data were extracted from electronic medical records and paper-based apheresis logs. Immediately after extraction, data were pseudonymized using unique study identification numbers. Direct identifiers (names, national identification numbers, exact dates of birth) were removed before analysis. The linking file connecting identifiers to study numbers was stored on a password-protected, encrypted hospital server accessible only to the principal investigator.

Confirmation of ethical compliance: The authors confirm that all research was performed in accordance with the ethical principles of the Belmont Report, the Declaration of Helsinki (2013 revision), and Egyptian national regulations for biomedical research.

Human Participants Checklist: The completed PLOS Human Participants Research Checklist is submitted as Supporting Information (S1 Checklist).

Revised Declarations Section (Ethics Statement - condensed):

Ethics approval and consent to participate: This study was approved by the Institutional Review Board of Zagazig University Faculty of Medicine (ZU-IRB #9357) as described in the Methods section (Ethical Considerations). The IRB waived the requirement for written informed consent as detailed in the Methods section. All methods were performed in accordance with relevant guidelines and regulations, including Egyptian national regulations (Ministerial Decree 296/2021) and the Declaration of Helsinki (2013).

________________________________________

Editorial Comment #4

Reviewer-Recommended Citations:

“ If the reviewer comments include a recommendation to cite specific previously published works, please review and evaluate these publications to determine whether they are relevant and should be cited. There is no requirement to cite these works unless the editor has indicated otherwise. “

Author Response:

We have carefully reviewed all reviewer comments to identify any recommendations to cite specific previously published works. No reviewer recommended any specific citations.

Reviewer #1's comments focused on manuscript length, study design, statistical modeling, and presentation issues. Reviewer #2's comments focused on model interpretation, patient classification, propensity score matching, and adverse event reporting. Neither reviewer requested the addition of any particular references.

Therefore, we have not added any new citations in response to this comment. We have verified that our existing reference list is comprehensive and appropriately supports the manuscript content. No additional citations were identified as necessary based on reviewer feedback.

Revised Manuscript Section:

No revisions required. The existing reference list remains unchanged as no reviewer recommended any specific citations. The reference list in the manuscript is complete and contains all necessary citations to support the content.

________________________________________

Response to Reviewer #1

A. Overall Assessment

Reviewer comment:

“This manuscript presents a retrospective seven-year cohort evaluating therapeutic plasma exchange (TPE) in an Egyptian tertiary center. The topic is clinically relevant because real-world evidence from low- and middle-income countries remains limited. The study includes 221 patients and evaluates efficacy, safety, and predictors of outcomes. While the work has merit, substantial revisions are required before publication.”

Response:

We thank the reviewer for the positive recognition of our work's clinical relevance and the acknowledgment that real-world evidence from low- and middle-income countries remains limited. We appreciate the reviewer's constructive assessment and have carefully addressed all substantial revisions requested in the subsequent comments.

B. Major Comments

1. Manuscript Length:

Reviewer comment:

“The manuscript is considerably longer than necessary. The Introduction, Methods, and Discussion should be reduced by approximately 30%, removing repeated methodological descriptions and excessive technical details.”

Author Response:

We respectfully acknowledge the reviewer's concern regarding manuscript length, but we would like to provide a rationale for the current structure and explain why many of the details the reviewer may perceive as "excessive" or "repeated" are, in our view, essential to the scientific integrity, reproducibility, and interpretability of this work.

Our Approach: Comprehensive Rather Than Redundant

We have carefully reviewed the manuscript for redundancy and have made targeted reductions of approximately 15–18% (rather than the full 30% requested) because we believe the current length is justified by several factors:

1. The Heterogeneity of the Cohort Necessitates Detailed Description

Unlike disease-specific TPE studies that focus on a single condition (e.g., TTP alone or GBS alone), our cohort spans renal, neurologic, hematologic, and metabolic indications—each with distinct pathophysiology, procedural requirements, and outcome measures. This heterogeneity demands:

Component Why It Is Necessary

Detailed disease-specific response criteria Without these, readers cannot interpret whether "response" means the same thing across GBS, TTP, SLE, and hypertriglyceridemia

Stratified baseline characteristics (Table 3) The profound differences in hemoglobin, platelets, and creatinine across groups are the foundation of our risk model—readers need to see these to understand why outcomes differ

Disease-specific outcome tables (Tables 5–8) Pooling all diseases into a single outcome table would mask the critical finding that TTP and GBS respond very differently from DAH and AMR

The heterogeneity is not a flaw; it is the central finding of the study. The length reflects the complexity of the dataset, not redundancy.

2. Reproducibility Requires Technical Detail

We fully agree that manuscripts should avoid unnecessary technical detail. However, we believe that transparency in methods is essential for reproducibility, particularly in resource-limited settings where protocols may need to be adapted.

The following technical details are not "excessive"; they are necessary for repli

Attachment Submitted filename: Response to Editorial & Reviewers Comments.docx

# Editor report: Decision Letter 1

**DOI:** 10.1371/journal.pone.0358069.r004

**Authors:** Awais ALi

26 Aug 2026

Therapeutic Plasma Exchange Across Multiple Organ Systems in an Egyptian Tertiary Center: A Seven-Year Real-World Cohort Study

PONE-D-26-30667R1

Dear Dr. Othman,

We’re pleased to inform you that your manuscript has been judged scientifically suitable for publication and will be formally accepted for publication once it meets all outstanding technical requirements.

Within one week, you’ll receive an e-mail detailing the required amendments. When these have been addressed, you’ll receive a formal acceptance letter and your manuscript will be scheduled for publication.

An invoice will be generated when your article is formally accepted. Please note, if your institution has a publishing partnership with PLOS and your article meets the relevant criteria, all or part of your publication costs will be covered. Please make sure your user information is up-to-date by logging into Editorial Manager at Editorial Manager® and clicking the ‘Update My Information' link at the top of the page. For questions related to billing, please contact billing support.

If your institution or institutions have a press office, please notify them about your upcoming paper to help maximize its impact. If they’ll be preparing press materials, please inform our press team as soon as possible -- no later than 48 hours after receiving the formal acceptance. Your manuscript will remain under strict press embargo until 2 pm Eastern Time on the date of publication. For more information, please contact onepress@plos.org.

Kind regards,

Dr. Awais ALi

Guest Editor

PLOS One

<img alt=" " aria-label=" " class="an1" data-emoji=" " draggable="false" loading="lazy" src="https://fonts.gstatic.com/s/e/notoemoji/17.0/1f517/72.png" style="height: 1.2em; width: 1.2em; vertical-align: middle;" /> ORCID: 0000-0002-4514-9509

**Loop Profile :**  2395035

<img alt=" " aria-label=" " class="an1" data-emoji=" " draggable="false" loading="lazy" src="https://fonts.gstatic.com/s/e/notoemoji/17.0/1f7e3/72.png" style="height: 1.2em; width: 1.2em; vertical-align: middle;" /> **SciProfiles:**  3367557

<img alt=" " aria-label=" " class="an1" data-emoji=" " draggable="false" loading="lazy" src="https://fonts.gstatic.com/s/e/notoemoji/17.0/1f7e0/72.png" style="height: 1.2em; width: 1.2em; vertical-align: middle;" /> **Scopus Author ID:**

Additional Editor Comments (optional):

Reviewers' comments:

# Editor report: Acceptance letter

**DOI:** 10.1371/journal.pone.0358069.r005

**Authors:** Awais ALi

PONE-D-26-30667R1

PLOS One

Dear Dr. Othman,

I'm pleased to inform you that your manuscript has been deemed suitable for publication in PLOS One. Congratulations! Your manuscript is now being handed over to our production team.

At this stage, our production department will prepare your paper for publication. This includes ensuring the following:

* All references, tables, and figures are properly cited

* All relevant supporting information is included in the manuscript submission,

* There are no issues that prevent the paper from being properly typeset

You will receive further instructions from the production team, including instructions on how to review your proof when it is ready. Please keep in mind that we are working through a large volume of accepted articles, so please give us a few days to review your paper and let you know the next and final steps.

Lastly, if your institution or institutions have a press office, please let them know about your upcoming paper now to help maximize its impact. If they'll be preparing press materials, please inform our press team within the next 48 hours. Your manuscript will remain under strict press embargo until 2 pm Eastern Time on the date of publication. For more information, please contact onepress@plos.org.

You will receive an invoice from PLOS for your publication fee after your manuscript has reached the completed accept phase. If you receive an email requesting payment before acceptance or for any other service, this may be a phishing scheme. Learn how to identify phishing emails and protect your accounts at https://explore.plos.org/phishing.

If we can help with anything else, please email us at customercare@plos.org.

Thank you for submitting your work to PLOS One and supporting open access.

Kind regards,

PLOS One Editorial Office Staff

on behalf of

Dr. Awais ALi

Guest Editor

PLOS One
